# Effects of an *EPSPS*-transgenic soybean line ZUTS31 on root-associated bacterial communities during field growth

**DOI:** 10.1371/journal.pone.0192008

**Published:** 2018-02-06

**Authors:** Gui-Hua Lu, Cheng-Yi Tang, Xiao-Mei Hua, Jing Cheng, Gu-Hao Wang, Yin-Ling Zhu, Li-Ya Zhang, Hui-Xia Shou, Jin-Liang Qi, Yong-Hua Yang

**Affiliations:** 1 NJU–NJFU Joint Institute for Plant Molecular Biology, State Key Laboratory of Pharmaceutical Biotechnology, School of Life Sciences, Nanjing University, Nanjing, China; 2 Jiangsu Collaborative Innovation Center for Modern Crop Production, Nanjing Agricultural University, Nanjing, China; 3 Research Center for Soil Pollution Prevention and Control, Nanjing Institute of Environmental Sciences, MEP, Nanjing, China; 4 Crop Research Institute, Anhui Academy of Agricultural Sciences, Hefei, China; 5 State Key Laboratory of Plant Physiology and Biochemistry, College of Life Sciences, Zhejiang University, Hangzhou, China; Estacion Experimental del Zaidin, SPAIN

## Abstract

The increased worldwide commercial cultivation of transgenic crops during the past 20 years is accompanied with potential effects on the soil microbial communities, because many rhizosphere and endosphere bacteria play important roles in promoting plant health and growth. Previous studies reported that transgenic plants exert differential effects on soil microbial communities, especially rhizobacteria. Thus, this study compared the soybean root-associated bacterial communities between a *5-enolpyruvylshikimate-3-phosphate synthase* -transgenic soybean line (ZUTS31 or simply Z31) and its recipient cultivar (Huachun3 or simply HC3) at the vegetative, flowering, and seed-filling stages. High-throughput sequencing of 16S rRNA gene (16S rDNA) V4 hypervariable region amplicons via Illumina MiSeq and real-time quantitative PCR (qPCR) were performed. Our results revealed no significant differences in the overall alpha diversity of root-associated bacterial communities at the three developmental stages and in the beta diversity of root-associated bacterial communities at the flowering stage between Z31 and HC3 under field growth. However, significant differences in the beta diversity of rhizosphere bacterial communities were found at the vegetative and seed-filling stages between the two groups. Furthermore, the results of next generation sequencing and qPCR showed that the relative abundances of root-associated main nitrogen-fixing bacterial genera, especially *Bradyrhizobium* in the roots, evidently changed from the flowering stage to the seed-filling stage. In conclusion, Z31 exerts transitory effects on the taxonomic diversity of rhizosphere bacterial communities at the vegetative and seed-filling stages compared to the control under field conditions. In addition, soybean developmental change evidently influences the main symbiotic nitrogen-fixing bacterial genera in the roots from the flowering stage to the seed-filling stage.

## Introduction

The global commercial cultivation of genetically modified / transgenic crops has increased from 1.7 million hectares in 1996 to 179.7 million hectares in 2015, and has accumulated 2 billion hectares during the past 20 years [1]. Up to 1 billion hectares of arable land are used for the global commercial cultivation of transgenic soybeans, especially glyphosate-tolerant (GT) soybeans [1,2].

Although these commercial transgenic crop varieties have brought massive economic benefits [1], their potential impact on soil rhizosphere bacterial communities (reviewed in [3–5]) has become a cause of concern because rhizosphere microbiota affects plant health and growth [6–9], in turn plants determine the composition, structure, and activity of rhizosphere microbiota via root exudates or rhizodeposits [8,10–15]. Recently, Edwards et al. have reported that rice genotypes affect microbial communities not only in the rhizosphere, but also in the rhizoplane and endosphere [16].

The release of numerous transgenic plants, including some transgenic soybean lines, exerts no significant effects or only minor and transitory effects on soil microbial communities [17–20] (also reviewed in [3–5]). In other cases, the release of transgenic plants has significant effects on soil microbial communities. In particular, the transgenic GT *Brassica napus* variety Quest significantly affects root-endophytic bacterial communities [21] or rhizosphere microbial communities throughout the growing season [22] compared with the conventional *B*. *napus* variety Excel. The composition of rhizosphere microbial communities of the herbicide-tolerant transgenic Zoysia grass differs from that of its nontransgenic Zoysia grass [23].The glyphosate-resistant transgenic soybean line BRS 245 RR affects soil microbial taxonomic and functional abundances compared with its parental conventional soybean cultivar BRS 133 [24]. The glyphosate-tolerant transgenic soybean line NZL06-968 significantly impacts the phylogenetic diversity of rhizosphere microbial communities compared with its control cultivar Mengdou12 [25]. The transgenic Roundup-Ready soybean cultivar negatively affects some parameters of biological nitrogen fixation [26], soil enzyme activities, or the structure of soil microbial communities (also reviewed in [4,5]) compared with nontransgenic soybean cultivar.

Previous studies reported differential results about the effect of transgenic plants on soil microbial communities, especially on rhizobacterial communities. Moreover, the highly complicated interaction between plants and rhizosphere microbial communities [27] and the high complexity of soil ecosystems [28,29] render further research on a case-by-case basis necessary to evaluate the effects of new transgenic plants on rhizobacterial communities.

High-throughput next-generation sequencing (NGS) technologies, such as 454 GS FLX pyrosequencing and Illumina HiSeq / MiSeq platform with corresponding bioinformatics tools, have facilitated microbial community research [8].

Rhizosphere bacterial communities change during soybean grown in the field [30], and the assemblage of rhizosphere microbiota is affected by *Arabidopsis* development [31]. In addition, bacterial endophytes play important roles in promoting plant health and growth [32,33]. Thus, in the present study, we compared the rhizosphere and root interior (endosphere) bacterial communities of the *5-enolpyruvylshikimate-3-phosphate synthase* (*EPSPS*)-transgenic soybean line ZUTS31 (Z31) versus its recipient cultivar Huachun3 (HC3) at the vegetative, flowering, and seed-filling stages under field growth conditions. In this analysis, the 16S rDNA-based Illumina MiSeq high-throughput NGS platform and quantitative PCR (qPCR) were used to clarify whether or not the release of Z31 affects root-associated bacterial communities.

## Materials and methods

### Plant materials

The recipient soybean cultivar was HC3, which usually grows in the Yangtze River basin and Southern China. The GT transgenic soybean line Z31, which is the offspring of the transgenic soybean homozygous line L1 prepared by Lu et al. [34], was integrated with a single copy of the *g10-epsps* gene that was cloned from GT *Deinococcus radiodurans* R1and also encodes the GT EPSPS. The insertion site of T-DNA and *g10-epsps* transgenes in ZUTS31 at the chromosome No. 5 of soybean was identified by TAIL-PCR.

### Field design and sampling

The experimental field (576 m^2^; N 31° 53′ 28′′–29′′, E 117° 14 ′ 22′′–23′′) is located in the Anhui Academy of Agricultural Sciences, Hefei City, Anhui Province, China. This field was divided into 48 plots (6 m × 2 m per plot) for national joint experiments in June 2014. Three replicate plots were used to each treatment, and randomly distributed over the field. Emerging weeds in those six plots for Z31 and HC3 plant growth were manually removed. The local soil type was clay pan yellow-cinnamon soils containing approximately 16.5% water content, 15.0 g/kg organic matter, 1.1 g/kg total nitrogen, 80.0 mg/kg available NH4^+^, 15.0 mg/kg available phosphorus, and 100.0 mg/kg available potassium, with a pH 5.58–5.75.

Samples of rhizospheric soil were collected as previously described by Inceoglu et al. [35]. In brief, two sampling points were determined in each of three plots. Every two samples of bulk soil were collected from each plot before sowing on June 18, 2014. Three plants at the late vegetative stage (V6–V7) and two plants at the flowering stage (R1–R2) or seed-filling stage (R5) with the surrounding soil were dug out from each sampling point and collected as one sample on July 20, July 31, and September 7, 2014, respectively. These plants were placed in a plastic bag with several chemical ice packs and immediately taken to the laboratory. Soil loosely adhering to the roots was shaken off from soybean plant as surrounding soil samples and then stored at 4°C for enzyme activity analysis or at −80°C in a freezer for DNA extraction. Afterwards, rhizospheric soil samples were collected by brushing off the soil that was tightly adhering to the root surface and then were stored at −80°C in a freezer for DNA extraction. Finally, the remaining rhizospheric soil was washed off from the roots with phosphate-buffered saline (PBS) and precipitated. Both rhizospheric soil and soybean roots were stored at −80°C in a freezer for DNA extraction.

### DNA extraction from soil and root samples

Total metagenomic DNA was extracted from approximately 2 × 0.60 g of soil of every biological replicate by using the PowerSoil DNA Isolation Kit (MoBio Laboratories Inc., Carlsbad, CA, USA) in accordance with the manufacturer’s instructions with minor modifications [25]. In addition, approximately 2 × 0.7 g of soybean root segments of every biological replicate was first carefully homogenized using a mortar and pestle under liquid nitrogen. Subsequently, total metagenomic DNA was extracted from homogenized root samples using the above method.

The metagenomic DNA concentration of each biological replicate was more than 10 ng /μL as measured by a Qubit Fluorometer (Qubit 2.0, Invitrogen, Carlsbad, USA), thereby minimizing the variability in surveys of microbial communities [36]. DNA integrity was examined by using 1% agarose gel electrophoresis before the DNA samples were stored at −20°C in a freezer.

### 16S rDNA amplicon sequencing via Illumina MiSeq platform

We used an improved dual-index high-throughput sequencing approach with paired-end 250 nt [37]. In brief, the fusion primers included the appropriate P5 or P7 Illumina adapter sequences, an 8 nt index sequence, and gene-specific primers for amplifying the V4 region of 16S rDNA, namely, 515F (5 ″- GTGCCAGCMGCCGCGGTAA-3″) and 806R (5 ″-GGACTACHVGGGTWTCTAAT-3″)[14,38]. PCR amplification, PCR product purification, library quality determination, and library quantification were performed as previously described by Lu et al. [25]. High-throughput sequencing of the qualified libraries was performed on the Illumina MiSeq platform (Illumina, CA, USA) with the MiSeq Reagent Kit by BGI Tech Solutions Co., Ltd. (Wuhan, China). Sequencing clean data of 100 samples have been submitted to the Sequence Read Archive (SRA) of NIH. The SRA study ID is SRP121636 (https://trace.ncbi.nlm.nih.gov/Traces/sra/sra.cgi?study=SRP121636).

### Analysis of 16S rDNA amplicon sequencing data

Operational taxonomic unit (OTU) was selected as previously described by Lu et al. [25] with minor modifications, that is, chimeras were filtered out by using UCHIME (v4.2.40) [39], and connected tags were filtered to eliminate low quality and short sequences using QIIME (v1.7.0) [40] before clean tags were obtained. After species annotation and phylogenetic relation construction was performed, we normalized the OTU counts in each sample’s library by using rarefaction corresponding to the sample with the least absolute OTU abundance (taxonomic tags) in the group. Then, alpha and beta diversity analyses were subsequently analyzed as described by Lu, G. et al. [25] with minor modifications. Principal coordinate analysis (PCoA) based on the weighted UniFrac (WUF) distance was performed with QIIME (v1.7.0), and PCoA based on the Bray–Curtis distance was carried out with the WGCAN package of R software. The unweighted pair-group method with arithmetic mean (UPGMA) clustering was conducted with QIIME (Version 1.7.0) as a type of hierarchical clustering method to interpret the distance matrix using average linkage.

### Quantification of *nifH* by real-time PCR

The relative abundance of the *nifH* gene was quantified by real-time qPCR with a CFX connect real-time PCR System (Bio-Rad, USA). The primer pairs of PolF–PolR [41] for the *nifH* gene and 338f–518r [42] for the 16S rDNA as internal control were used. PCR amplification was carried out in 20 μL reaction volumes containing 50 ng metagenomic DNA, 0.5 μL of each primer (5 pM), and 10 μL of 2× SYBR Green mixture (FastStart Universal Probe Master, Roche Life Science, Switzerland). Water was added to make the final reaction volume. The PCR amplification was performed under the following conditions: 95°C for 10 min, followed by 40 cycles of 15 s at 95°C and 1 min at 60°C. Fluorescence was measured at the end of each cycle. All qPCR reactions were run in triplicate with each DNA sample extracted from each of six or four soil or root samples. For each run, the melting curve was analyzed to ensure specific assessment of the *nifH* gene. The relative gene level was calculated by using the 2^-Δ*CT*^ method [43].

### Statistical analyses

Wilcoxon tank-sum test and Tukey’s honest significance difference (HSD) test were used to examine the significance of alpha diversity indices. Analysis of similarities (ANOSIM), multiple response permutation procedure (MRPP) and Adonis (permutational multivariate analysis of variance) [44], which are complementary nonparametric analyses and based on Bray–Curtis distance, were performed by using the vegan package of software R (v3.1.3). Metastats [45] was used to obtain the abundance differences of microbial communities between samples (group = 2; samples per group ≥ 4). The obtained *p*-value was adjusted with Benjamini–Hochberg false discovery rate [46] correction (function “p.adjust” in the stats package of software R (v3.1.3)).

### Ethics statement

The Ministry of Agriculture of the People’s Republic of China issued permissions for the location. The field studies did not involve endangered species. The experimental field was not protected or privately owned in any way.

## Results

### Overall analysis of 16S rDNA amplicon sequencing data by Illumina MiSeq

High-throughput sequencing of 16S rDNA (V4 region) amplicons on the Illumina MiSeq platform was performed to characterize the bacterial community composition and structure in the bulk soil, surrounding soil, rhizospheric soil, and roots of the *EPSPS*-transgenic soybean line Z31 and its recipient cultivar HC3 at the vegetative, flowering, and seed-filling stages.

A total of 2,007,848 qualified paired clean reads with an average count per sample of 62,745 (range: 51,568–69,215) were obtained from the bulk, surrounding and rhizospheric soils at the vegetative stage. In addition, 102,671 OTUs, except singletons, were identified with an average of 3208 OTUs per sample (S1 Table). The OTU counts in each sample’s library were normalized using rarefaction corresponding to the sample Z31BSO1 (S1 Table) with the least absolute OTU abundance (37,694 taxonomic tags) in this group. All normalized OTU abundances of the bulk, surrounding and rhizospheric soils at the vegetative stage were summarized with species annotation in S2 Table.

Moreover, 1,975,338 qualified paired clean reads with an average count per sample of 61,729 (range: 40,052–79,542) were obtained from the surrounding soil, rhizospheric soil, and roots at the flowering stage. A total of 78,094 OTUs, except singletons, were identified with an average of 2440 OTUs per sample (S3 Table). The OTU counts in each sample’s library were normalized using rarefaction corresponding to the sample HC3CRt6 (S3 Table) with the least absolute OTU abundance (37,383 taxonomic tags) in this group. All normalized OTU abundances of these samples at the flowering stage were summarized with species annotation in S4 Table.

Furthermore, 2,371,418 qualified paired clean reads with an average count per sample of 65,873 (range: 38,447–129,173) were obtained from the surrounding soil, rhizospheric soil, and roots at the seed-filling stage. A total of 84,452 OTUs, except singletons, were identified with an average of 2346 OTUs per sample (S5 Table). The OTU counts in each sample’s library were normalized using rarefaction corresponding to the sample Z31DSO5 (S5 Table) with the least absolute OTU abundance (28,540 taxonomic tags) in this group. All normalized OTU abundances of these samples at the seed-filling stage were summarized with species annotation in S6 Table.

Additionally, the analysis codes of above 100 samples’ clean data were listed in S7 Table.

### Comparative analysis of the alpha diversity of root-associated bacterial communities between Z31 and HC3

Basing from the normalization of OTU counts in each sample’s library at different developmental stages, we calculated the rarefaction curves of the observed OTU number of 32 samples at the vegetative stage (S1 Fig), 32 samples at the flowering stage (S2 Fig), and 36 samples at the seed-filling stage (S3 Fig). All their curves nearly reached the saturation plateau (S1–S3 Figs), indicating that the sequencing depth and the OTU coverage included sufficient detectable species in bacterial communities and captured the diversity of bacterial communities at the vegetative, flowering, and seed-filling stages. Moreover, accumulation curves of the observed OTU number of those 32 samples at the vegetative stage (S4 Fig), 32 samples at the flowering stage (S5 Fig), and 36 samples at the seed-filling stage (S6 Fig) almost reached the saturation plateau, suggesting that the sampling number was also sufficient to cover enough detectable species in each of the three groups.

We calculated the mean and standard deviation (SD) of six alpha diversity indices of sequencing data for rhizospheric soil at the vegetative stage (S8 Table), rhizospheric soil and roots at the flowering stage (S9 Table), and rhizospheric soil and roots at the seed-filling stage (S10 Table) with bulk soil (S11 Table) as the blank control and surrounding soil at the vegetative stage (S12 Table), the flowering stage (S13 Table), and the seed-filling stage (S14 Table) as the systematic control.

All *p*-values of six indices of alpha diversity in S8–S14 Tables, which were examined between the samples of Z31 and HC3 by both Wilcoxon rank-sum test and Tukey’s HSD test, were higher than 0.05. Therefore, no statistically significant difference in the overall indices of the alpha diversity were observed between the root-associated bacterial communities of Z31 and those of HC3 in comparative analysis when the bulk soil and/or surrounding soil between Z31 and HC3 were used as control at the vegetative, flowering, and seed-filling stages. However, we observed that the rhizospheric soil of Z31 and HC3 had the largest community richness and Shannon diversity at the vegetative stage, whereas the roots of Z31 and HC3 had the lowest community rich ness and Shannon diversity at the flowering stage (Table 1).

**Table 1:** Comparison of observed_OTUs and Shannon indices of root-associated bacterial communities between the transgenic soybean line Z31 and its recipient cultivar HC3 at different developmental stages.

Developmental stage	Sample	Observed_OTU (Mean ± SD)	*p*-value (Wilcoxon)	Shannon (Mean ± SD)	*p*-value (Wilcoxon)
Vegetative	Z31BRh	2855.83 ± 139.36	0.39178	9.4170 ± 0.2996	0.86862
	HC3BRh	2982.00 ± 253.55		9.4308 ± 0.3835	
Flowering	Z31CRh	2800.17 ± 75.44	0.32789	9.3520 ± 0.0852	0.18843
	HC3CRh	2724.17 ± 161.29		9.1755 ± 0.2364	
	Z31CRt	310.25 ± 39.31	0.89870	2.1005 ± 0.2448	0.87200
	HC3CRt	299.75 ± 35.80		2.0003 ± 0.0991	
Seed-filling	Z31DRh	2412.50 ± 166.50	0.21003	9.1365 ± 0.3049	0.40089
	HC3DRh	2511.83 ± 118.87		9.2725 ± 0.1405	
	Z31DRt	703.67 ± 32.28	0.28706	4.1143 ± 0.2189	0.37411
	HC3DRt	668.50 ± 79.90		3.7895 ± 0.3667	

SD represents standard deviation. The significance test method was Wilcoxon rank-sum test (Wilcoxon). Values were mean ± SD (n = 4 or 6). Z31BRh, Z31CRh, and Z31DRh represent the rhizospheric bacterial communities of Z31 at the vegetative, flowering, and seed-filling stages, respectively. HC3BRh, HC3CRh, and HC3DRh represent the rhizospheric bacterial communities of HC3 at the vegetative, flowering, and seed-filling stages, respectively. Z31CRt and Z31DRt represent the root bacterial communities of Z31 at the flowering and seed-filling stages, respectively. HC3CRt and HC3DRt represent the root bacterial community of HC3 at the flowering and seed-filling stages, respectively.

### Comparative analysis of the beta diversity of root-associated bacterial communities between Z31 and HC3

#### Beta diversity of rhizosphere bacterial communities between Z31 and HC3 at the vegetative stage

**Fig 1:**
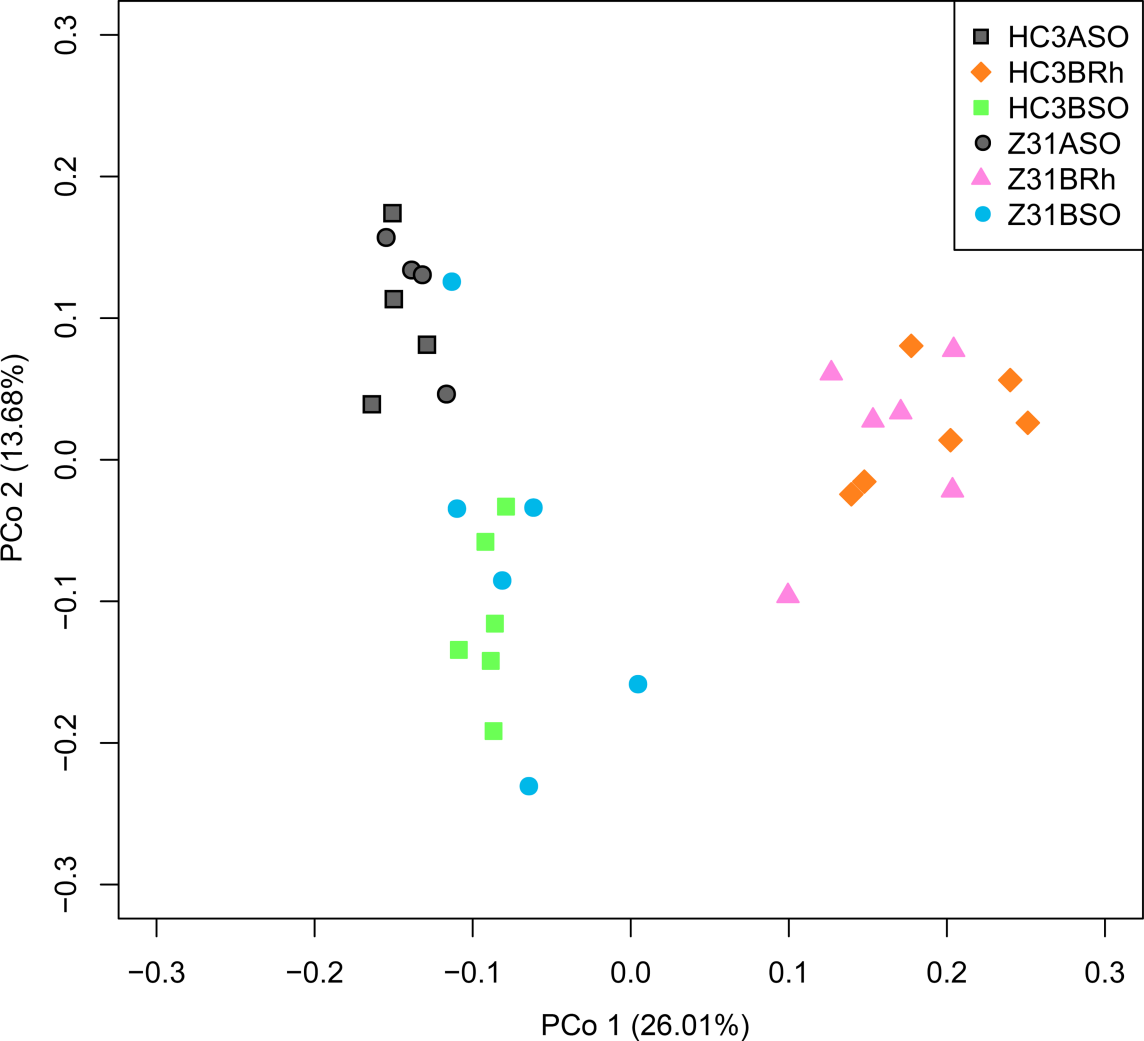
Principal coordinate analysis (PCoA) based on the Bray–Curtis distance metrics of bacterial communities between the 5-enolpyruvylshikimate-3-phosphate synthase (*EPSPS*)-transgenic soybean line ZUTS31 (Z31) and its recipient cultivar Huachun3 (HC3) at the vegetative stage (n = 32). HC3BRh and Z31BRh represent the rhizospheric soils of the recipient cultivar HC3 and the transgenic line Z31 at the vegetative stage, respectively. HC3BSO and Z31BSO represent the surrounding soils of the recipient cultivar HC3 and the transgenic line Z31 at the vegetative stage, respectively. HC3ASO and Z31ASO represent the bulk soils before sowing seeds of the recipient cultivar HC3 and the transgenic line Z31, respectively.

First, we used principal component analysis (PCA) to examine the differences in the OTU composition of the bulk, surrounding, and rhizospheric soil samples between Z31 and HC3 at the vegetative stage. The bacterial communities in the rhizospheric soil, bulk soil and surrounding soil of Z31 were not distinct from those of HC3 (S7A Fig). Then, we performed phylogenetic beta diversity analysis by PCoA based on the WUF distance metrics. The bacterial communities in the rhizospheric, bulk, and surrounding soil samples of Z31 also exhibited no distinction from those of HC3 (S7B Fig). Furthermore, we conducted nonmetric multidimensional scaling (NMDS) analysis based on the Bray–Curtis distance metrics. The bacterial communities in the rhizospheric, bulk, and surrounding soil samples of Z31 overlapped with those of HC3 (S7C Fig). The stress value (S7C Fig) was less than 0.2, indicating that NMDS analysis results were reliable. Additionally, we conducted taxonomic beta diversity analysis by PCoA based on the Bray–Curtis distance metrics. The bacterial communities in the rhizospheric, bulk, and surrounding soil samples of Z31 also displayed no distinction from those of HC3 (Fig 1). However, Adonis analysis based on the Bray–Curtis distance indicated that the taxonomic beta diversity of bacterial communities in the rhizospheric soil of Z31 was significantly (*Pr* < 0.01) different from that of HC3 (Table 2, and S15 Table in detail).Furthermore, we supplemented MRPP (Table 2, and S16 Table in detail) and ANOSIM (Table 2, and S17 Table in detail) statistical analysis results which were consistent with Adonis analysis.

**Table 2:** Significance tests of bacterial community structure between the transgenic soybean line Z31 and its recipient cultivar HC3 at different stages with three different statistical approaches.

Group vs. Group	Adonis ^1^		MRPP ^1^		ANOSIM ^1^	
	F. Model	*Pr* value ^2^	Observed delta	*P*-value ^2^	*R*-value	*P*-value ^2^
Z31ASO vs. HC3ASO	0.9985	0.402	0.2840	0.429	-0.1042	0.850
Z31BSO vs. HC3BSO	1.2876	0.154	0.3418	0.122	0.0907	0.104
Z31BRh vs. HC3BRh	1.6392	**0.005**	0.3367	**0.006**	0.1963	**0.010**
Z31CSO vs. HC3CSO	1.0684	0.335	0.3181	0.373	0.0204	0.392
Z31CRh vs. HC3CRh	1.4249	0.101	0.3080	0.072	0.1333	0.133
Z31CRt vs. HC3CRt	1.7077	0.081	0.06697	0.055	0.1823	0.109
Z31DSO vs. HC3DSO	1.5566	0.097	0.3284	0.120	0.1167	0.159
Z31DRh vs. HC3DRh	1.6885	**0.041**	0.3751	0.051	0.2139	0.056
Z31DRt vs. HC3DRt	0.8186	0.536	0.2644	0.478	-0.0574	0.626

Abbreviations: MRPP, multiple response permutation procedure; ANOSIM, analysis of similarities; Adonis, i.e., PERMANOVA, permutational multivariate analysis of variance; ASO, bulk soil was collected from field before sowing soybean seeds; BSO, surrounding soil at the vegetative stage; BRh, rhizospheric soil at the vegetative stage; CSO, surrounding soil at the flowering stage; CRh, rhizospheric soil at the flowering stage; CRt, roots at the flowering stage; DSO, surrounding soil at the seed-filling stage; DRh, rhizospheric soil at the seed-filling stage; DRt, root endosphere at the seed-filling stage.

1. ADONIS, MRPP, and ANOSIM, which are complementary nonparametric analyses, were performed by using the vegan package of software R (v3.1.3) based on the Bray–Curtis distance metrics.

*2*. *P*-value of corresponding significance test. The *P*-values in bold indicate the significant difference (*P* < 0.05, or *P* < 0.01) between the Z31 and HC3 groups by the tests.

#### Beta diversity of root-associated bacterial community between Z31 and HC3 at the flowering stage

**Fig 2:**
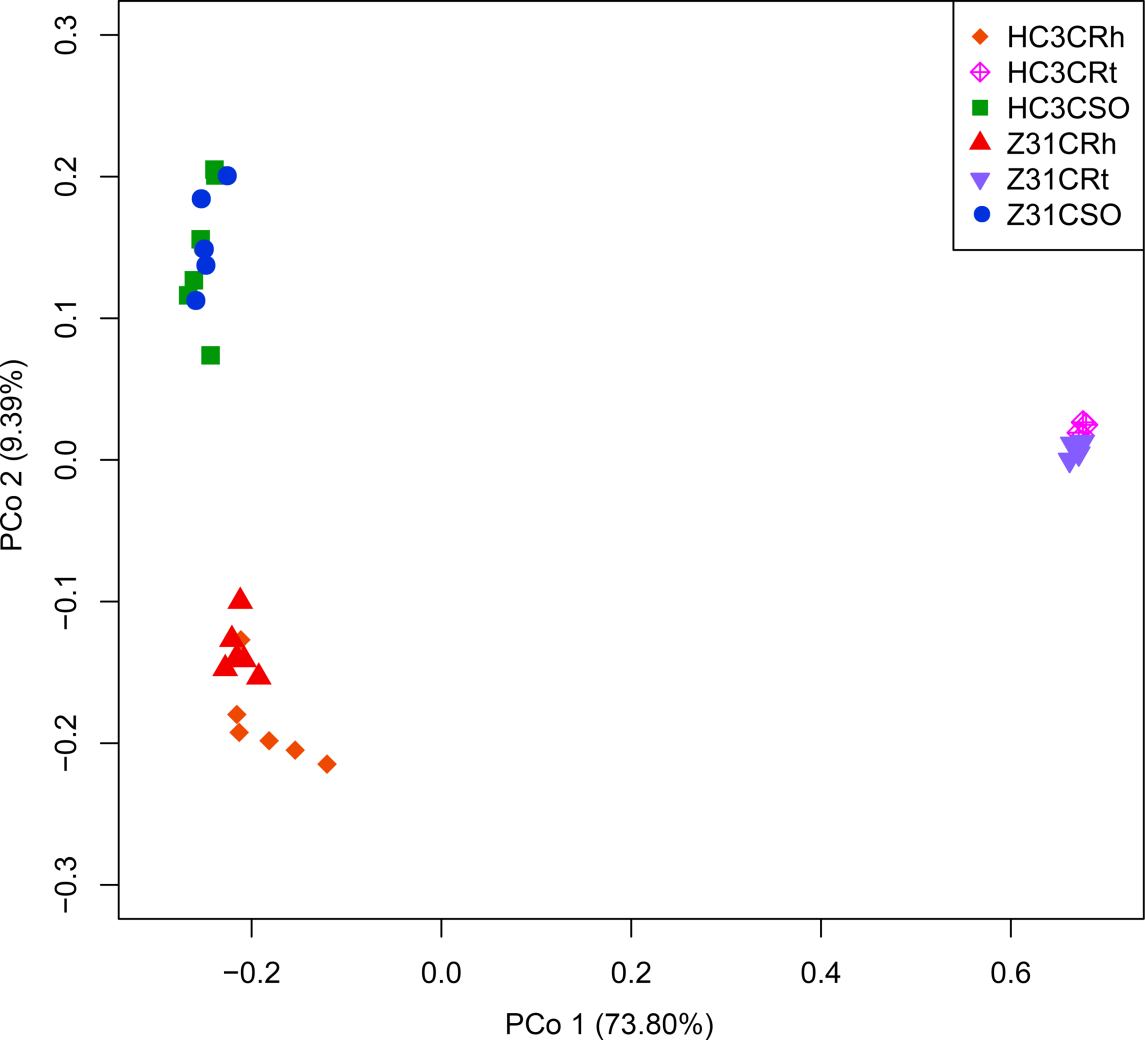
PCoA based on the Bray–Curtis distance metrics of root-associated bacterial communities between Z31 and HC3 at the flowering stage (n = 32). HC3CRt and Z31CRt represent the roots of the recipient cultivar HC3 and the transgenic line Z31 at the flowering stage, respectively. HC3CRh and Z31CRh represent the rhizospheric soils of the recipient cultivar HC3 and the transgenic line Z31 at the flowering stage, respectively. HC3CSO and Z31CSO represent the surrounding soils of the recipient cultivar HC3 and the transgenic line Z31 at the flowering stage, respectively.

At the flowering stage, the results of PCA (S8A Fig) and PCoA based on the WUF distance metrics (S8B Fig) showed no distinct differences between bacterial communities in the rhizospheric soil, root, and surrounding soil of Z31 and those of HC3. PCoA was further used to analyze the taxonomic beta diversity based on the Bray–Curtis distance metrics. The obtained results depicted that the bacterial communities in the rhizospheric soil of Z31 seemed to be distinct from those in the rhizospheric soil of HC3, whereas the bacterial communities in the root or surrounding soil of Z31 were not distinct from those of HC3 (Fig 2). However, MRPP (Table 2 and S18 Table in detail), ANOSIM (Table 2 and S19 Table in detail), and Adonis (Table 2 and S20 Table in detail) statistical analysis results indicated that the bacterial communities in the rhizospheric soil or roots of Z31 were not statistically (*P* > 0.05) distinct from those of HC3. In addition, NMDS analysis results implied that the bacterial communities in the rhizospheric soil, root, and surrounding soil of Z31 overlapped with those of HC3 (S8C Fig).

#### Beta diversity of root-associated bacterial communities between Z31 and HC3 at the seed-filling stage

**Fig 3:**
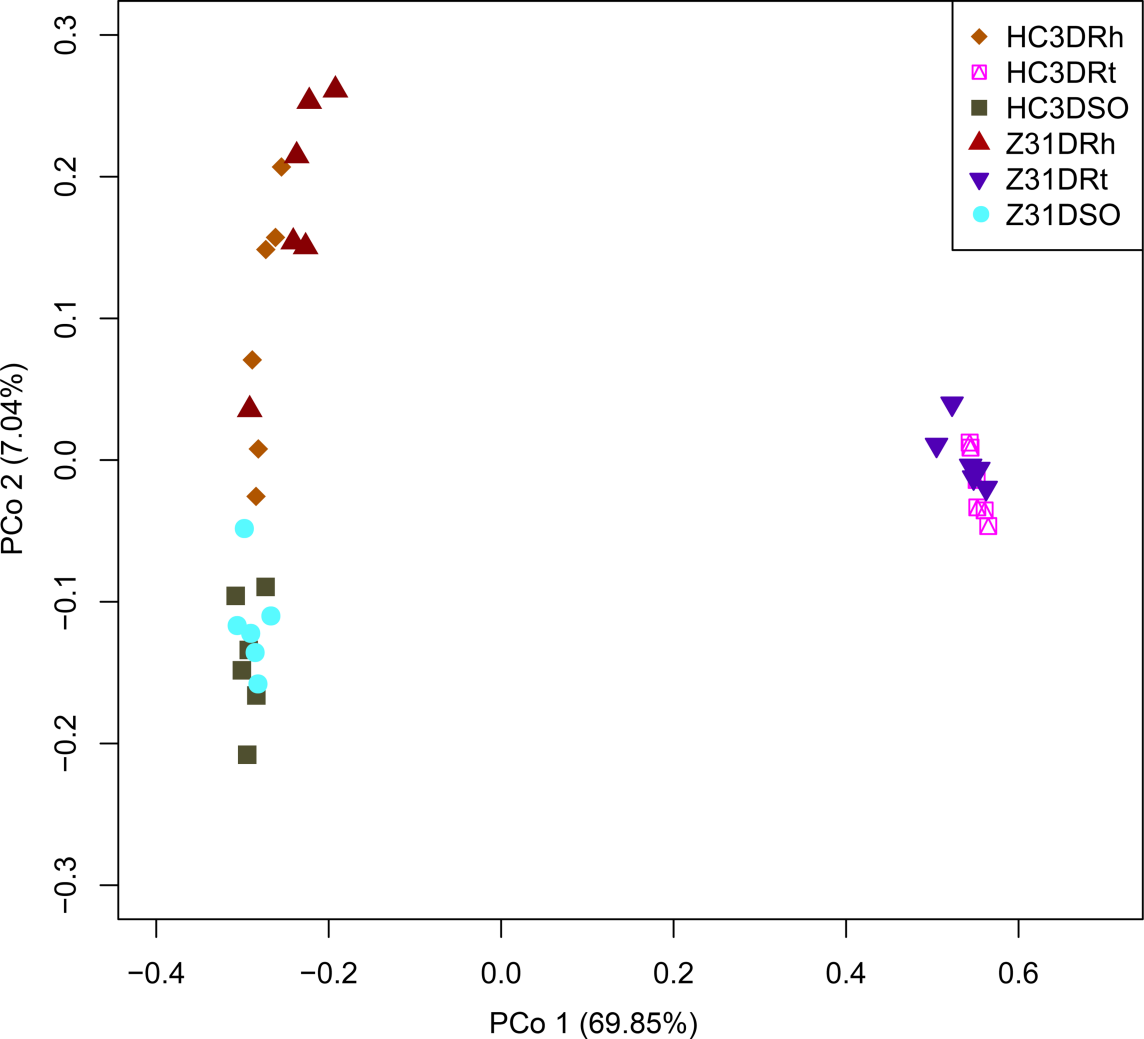
PCoA based on the Bray–Curtis distance metrics of root-associated bacterial communities between Z31 and HC3 at the seed-filling stage (n = 36). HC3DRt and Z31DRt represent the roots of the recipient cultivar HC3 and the transgenic line Z31 at the seed-filling stage, respectively. HC3DRh and Z31DRh represent the rhizospheric soils of the recipient cultivar HC3 and the transgenic line Z31 at the seed-filling stage, respectively. HC3DSO and Z31DSO represent the surrounding soils of the recipient cultivar HC3 and the transgenic line Z31 at the seed-filling stage, respectively.

The results of PCA (S9A Fig) and PCoA based on the WUF distance metrics result (S9B Fig) at the seed-filling stage showed that the bacterial communities in the rhizospheric soil, root, and surrounding soil of Z31 were not distinct from those of HC3. Furthermore, we performed NMDS analysis based on the Bray–Curtis distance metrics, which showed that the bacterial communities in the rhizospheric soil, roots, or surrounding soil of Z31 overlapped with those of HC3 (S9C Fig). Finally, the results of PCoA based on Bray–Curtis distance metrics displayed that the bacterial communities in the rhizospheric soil, root, and surrounding soil of Z31 overlapped with those of HC3 because the bacterial community in the surrounding soil of Z31 also overlapped with those of HC3 (Fig 3). The results of MRPP (Table 2 and S21 Table in detail) and ANOSIM (Table 2 and S22 Table in detail) indicated that the bacterial communities in the rhizospheric soil or roots of Z31 showed no statistical (*P* > 0.05) distinction from those of HC3 at the seed-filling stage. However, the results of Adonis statistical analysis (Table 2 and S23 Table in detail) revealed differences in taxonomic beta diversity between the rhizospheric soil of Z31 and that of HC3.

### Comparison of the composition of the major bacterial phyla at different developmental stages

The taxonomic composition of major bacterial phyla in the rhizosphere of the transgenic line Z31 and its recipient HC3 at the vegetative stage (S10 Fig) displayed Proteobacteria to be the most abundant phylum followed by Acidobacteria, Bacteroidetes, Actinobacteria, Planctomycetes, Verrucomicrobia, and Gemmatimonadetes. Based on the bulk soil as a blank control (S24 Table, sheet 1) and the surrounding soil as a systematic control (S24 Table, sheet 2), the relative abundances of Acidobacteria, Gemmatimonadetes, and Actinobacteria in the rhizospheric soil of Z31 might be different from those of HC3 (S24 Table, sheet 3).

At the flowering stage, Proteobacteria was also the most abundant phylum in the rhizosphere of soybean Z31 and HC3 (S11 Fig), followed by Acidobacteria, Bacteroidetes, Verrucomicrobia, Planctomycetes, or Actinobacteria. On the basis of the surrounding soil as a systematic control (S25 Table, sheet 1), the relative abundances of Proteobacteria, Acidobacteria, and Bacteroidetes in the rhizospheric soil of Z31 should be different from those of HC3 (S25 Table, sheet 2).

The major bacterial phyla at the seed-filling stage (S12 Fig) were Proteobacteria followed by Bacteroidetes or Acidobacteria, Verrucomicrobia, Planctomycetes, and Actinobacteria in the rhizospheric soil, followed by Cyanobacteria (Chloroplast), Actinobacteria, and Bacteroidetes in the roots of soybean Z31 and HC3. The relative abundances of the dominating phylum, Proteobacteria, in the rhizospheric soil of Z31 should be different from those of HC3 (S26 Table, sheet 2) based on the surrounding soil as a systematic control (S26 Table, sheet 1).

### Comparison of the composition of main nitrogen-fixing bacterial genera at different developmental stages

**Fig 4:**
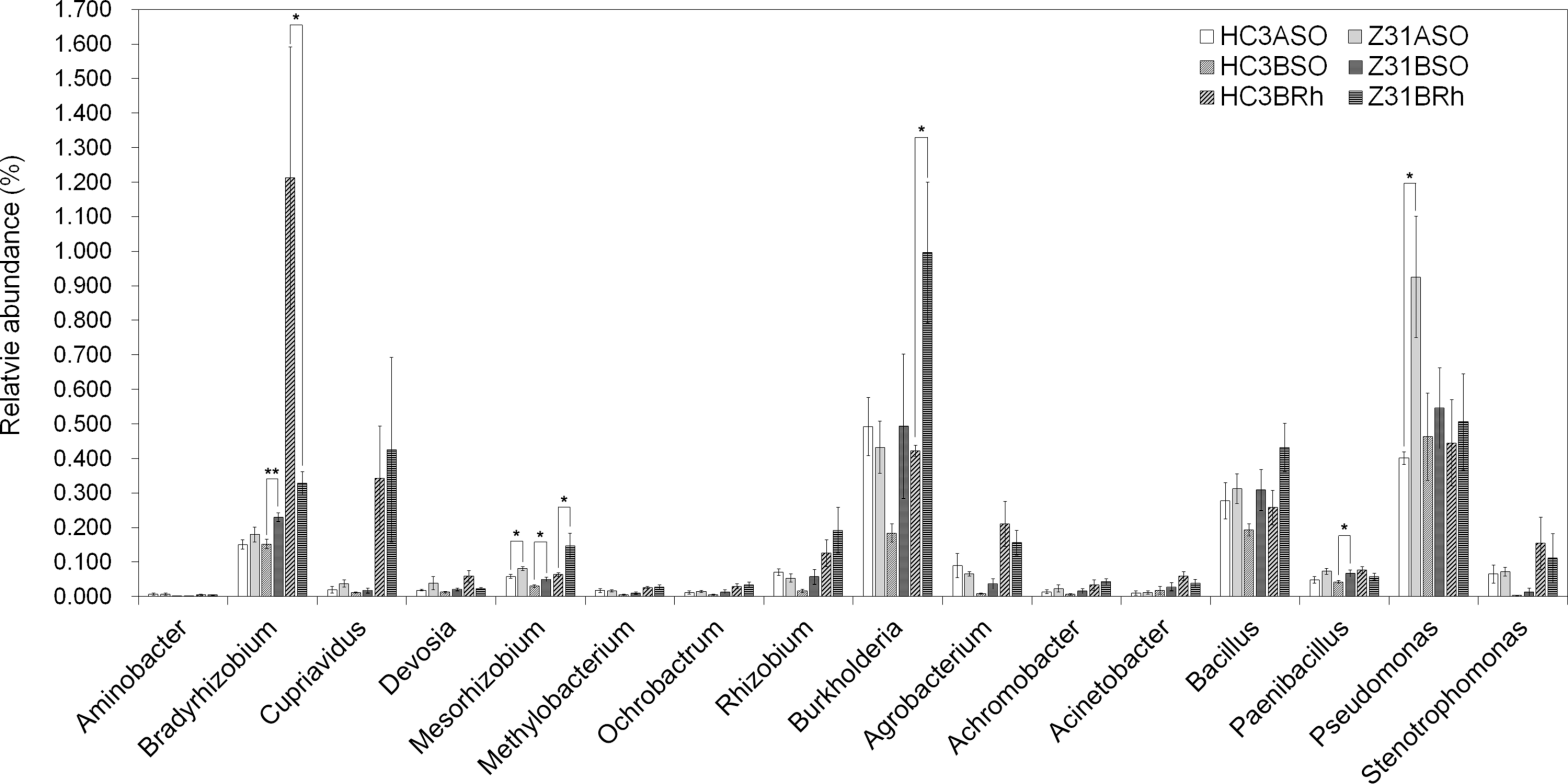
Relative abundances of main nitrogen-fixing bacterial genera at the vegetative stage. HC3BRh and Z31BRh represent the rhizospheric soils of the recipient cultivar HC3 and the transgenic line Z31 at the vegetative stage, respectively. HC3BSO and Z31BSO represent the surrounding soils of the recipient cultivar HC3 and the transgenic line Z31 at the vegetative stage, respectively. HC3ASO and Z31ASO represent the bulk soils before sowing seeds of the recipient cultivar HC3 and the transgenic line Z31, respectively. Error bars indicate the standard errors (SE) calculated by Metastats. Values are mean ± SE (n = 4 or 6);*, *P* < 0.05; **, *P* < 0.01.

A total of 275 identified genera were detected in the bulk, surrounding, and rhizospheric soil samples of Z31 and HC3 at the vegetative stage (S27 Table, sheet 1–3). Concerning the main nitrogen-fixing bacterial genera, the relative abundance of symbiotic *Burkholderia* in the rhizospheric soil of Z31was significantly higher whereas that of symbiotic *Bradyrhizobium* was significantly lower than that of HC3. This result was based on the contrast analysis of surrounding soils as systematic control and bulk soils as blank control (Fig 4).In addition, the relative abundance of symbiotic *Mesorhizobium* in the rhizospheric soil was not significantly affected by Z31 as it was significantly higher not only in the rhizospheric soil but also in the bulk and surrounding soils of Z31 compared with HC3 (Fig 4).

**Fig 5:**
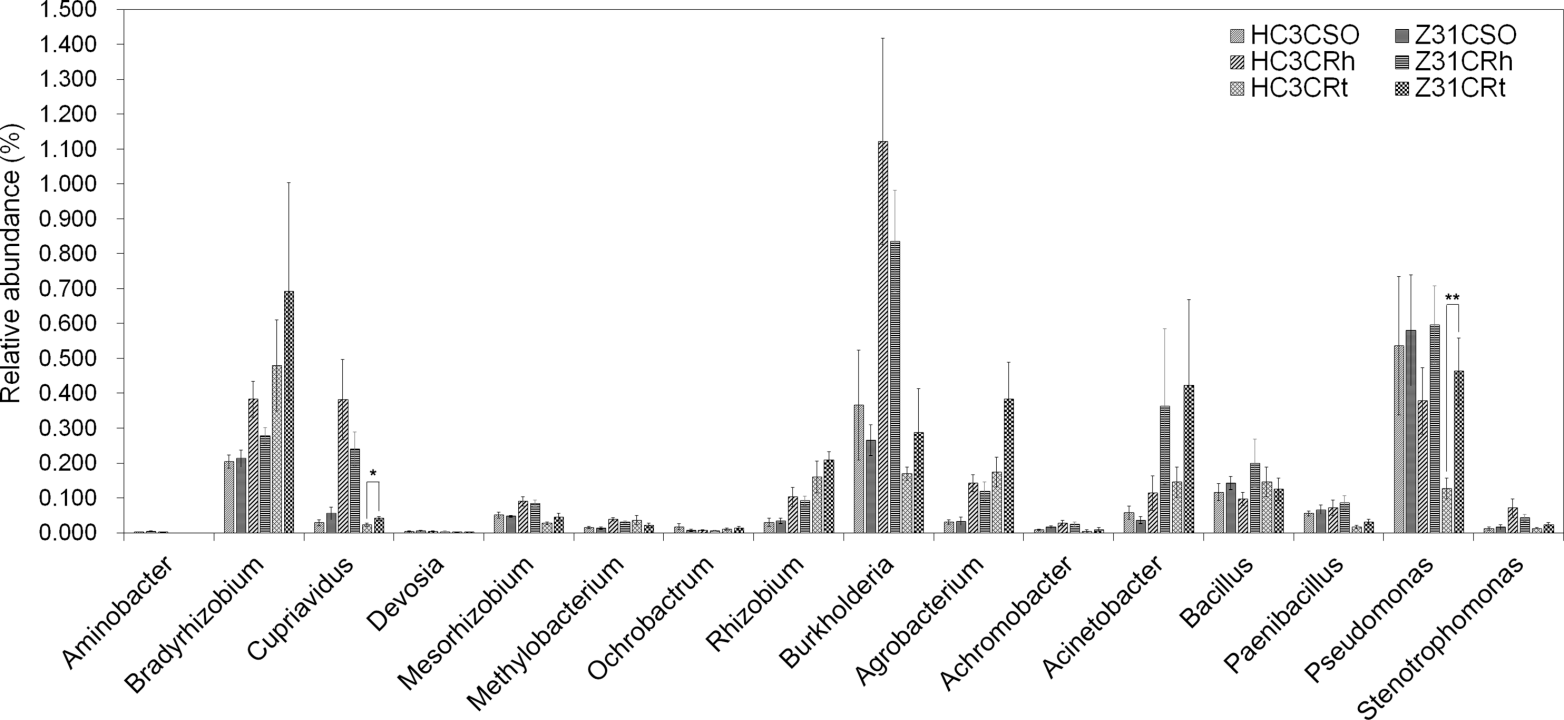
Relative abundances of main nitrogen-fixing bacterial genera at the flowering stage. HC3CRt and Z31CRt represent the roots of the recipient cultivar HC3 and the transgenic line Z31 at the flowering stage, respectively. HC3CRh and Z31CRh represent the rhizospheric soils of the recipient cultivar HC3 and the transgenic line Z31 at the flowering stage, respectively. HC3CSO and Z31CSO represent the surrounding soils of the recipient cultivar HC3 and the transgenic line Z31 at the flowering stage, respectively. Error bars indicate SE calculated by Metastats. Values are mean ± SE (n = 4 or 6); *, *P* < 0.05; **, *P* < 0.01.

Moreover, 257 identified genera were detected at the flowering stage (S28 Table, sheet 1–3). For the main nitrogen-fixing bacterial genera at this stage, the relative abundances of all those genera in the rhizospheric soil showed no significant difference between Z31 and HC3. This result was based on the contrast analysis of surrounding soils as systematic control (Fig 5). Moreover, the relative abundances of most of those genera in the roots at the flowering stage exhibited no significant difference between Z31 and HC3. However, the relative abundances of symbiotic diazotroph *Cupriavidus* and associated diazotroph *Pseudomonas* were significantly higher in the roots of Z31 than in those of HC3 (Fig 5).

**Fig 6:**
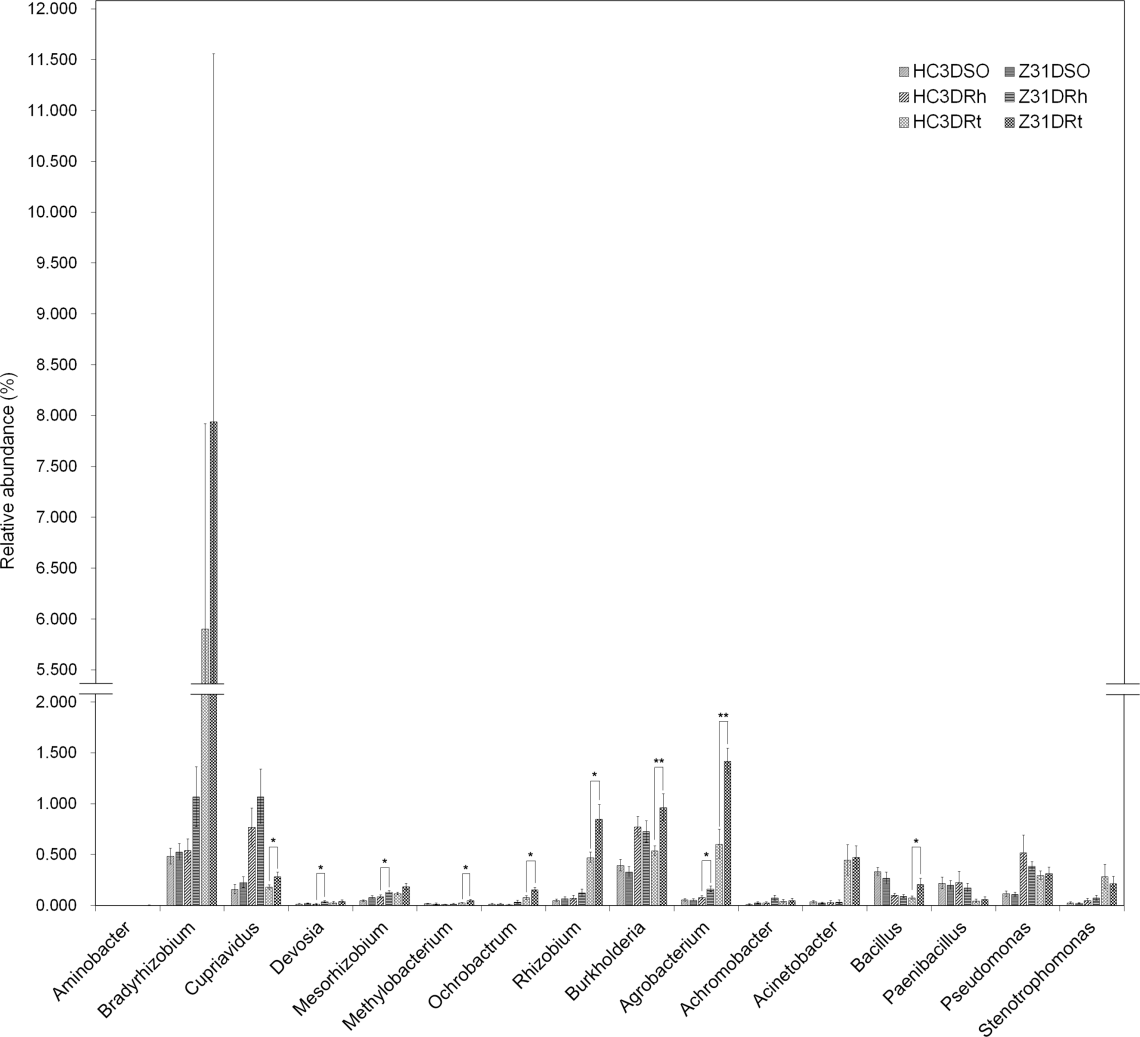
Relative abundances of main nitrogen-fixing bacterial genera at the seed-filling stage. HC3DRt and Z31DRt represent the roots of the recipient cultivar HC3 and the transgenic line Z31 at the seed-filling stage, respectively. HC3DRh and Z31DRh represent the rhizospheric soils of the recipient cultivar HC3 and the transgenic line Z31 at the seed-filling stage, respectively. HC3DSO and Z31DSO represent the surrounding soils of the recipient cultivar HC3 and the transgenic line Z31 at the seed-filling stage, respectively. Error bars indicate SE calculated by Metastats. Values are mean ± SE (n = 6); *, *P* < 0.05; **, *P* < 0.01.

Furthermore, 290 genera were identified at the seed-filling stage (S29 Table, sheet 1–3). With regard to the main nitrogen-fixing bacterial genera, the relative abundances of *Devosia*, *Mesorhizobium*, and *Agrobacterium* were significantly higher in the rhizospheric soil of Z31 than in that of HC3 (Fig 6). In addition, the relative abundances of *Cupriavidus*, *Methylobacterium*, *Ochrobactrum*, *Rhizobium*, *Burkholderia*, *Agrobacterium* and associated diazotroph *Bacillus* were significantly higher in the roots of Z31 than in those of HC3 (Fig 6). Remarkably, the relative abundance of symbiotic diazotroph *Bradyrhizobium* increased considerably in the roots of Z31and HC3 at the seed-filling stage (Fig 6).

### Comparison of the relative abundance of the *nifH* gene at the flowering and seed-filling stages

**Fig 7:**
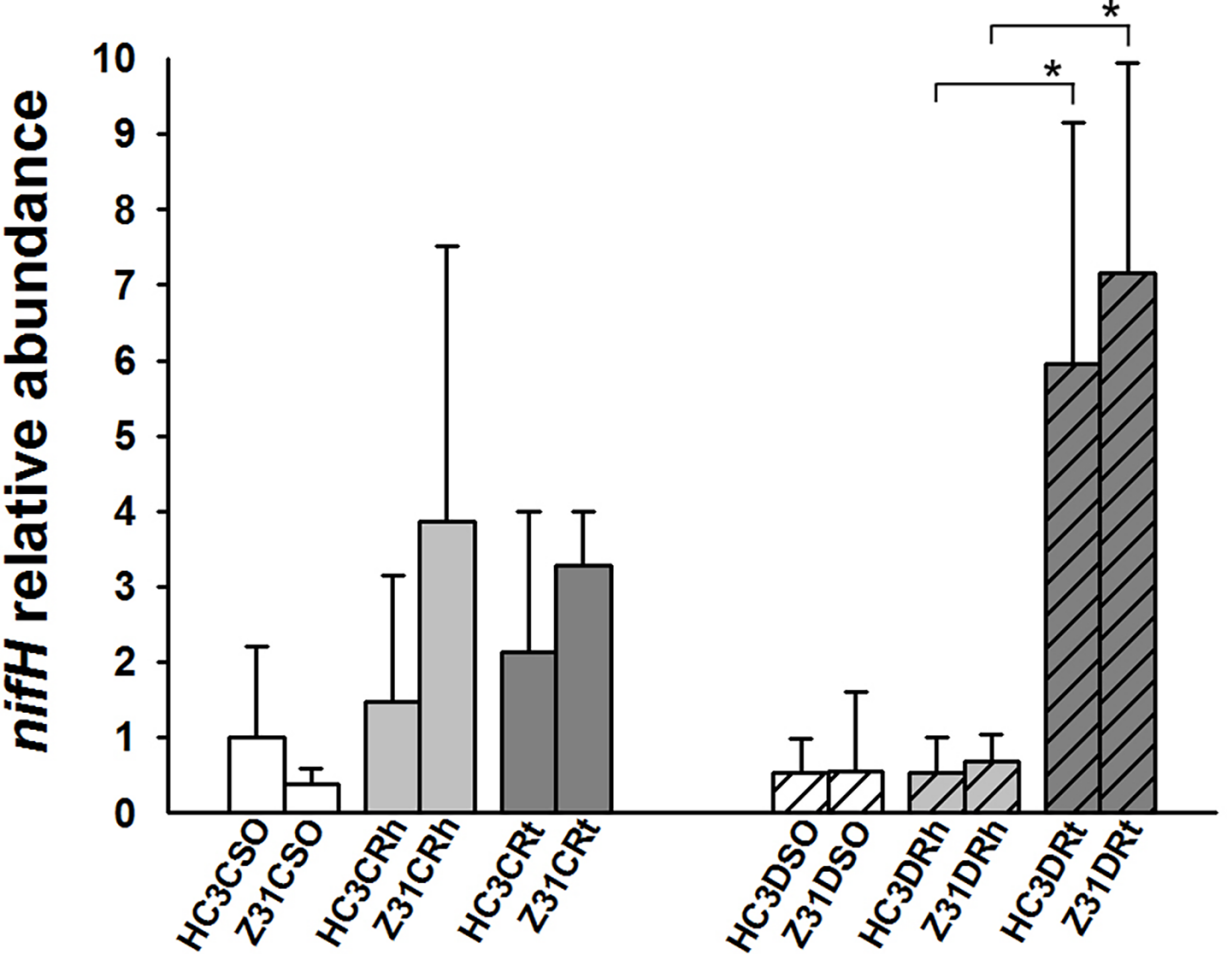
Relative abundance of the *nifH* gene in soybean root-associated bacterial communities of Z31 and HC3 at the flowering and seed-filling stages. CRt and DRt represent the roots of soybean at the flowering and seed-filling stages, respectively. CRh and DRh represent the rhizospheric soils of soybean at the flowering and seed-filling stages, respectively. CSO and DSO represent the surrounding soils of soybean at the flowering and seed-filling stages, respectively. Levels of the *nifH* gene abundance were normalized to 16S rDNA abundance. A value of 1 was assigned to the detected value of the surrounding soil of HC3 at the flowering stage (HC3CSO). The error bars represent the standard deviation of four or six replicates of soil or root samples and each replicate with technical triplicate. *Indicates significant difference (*P* < 0.05) according to the one-way ANOVA.

The relative abundance of *Bradyrhizobium* increased considerably in the roots of Z31and HC3 at the seed-filling stage. Thus, we determined the number of *nifH* gene copies by qPCR, and found no significant differences in the relative abundance of the *nifH* gene between the rhizosphere and roots of Z31 and those of HC3 at the flowering and seed-filling stages (Fig 7). However, the relative abundance of the *nifH* gene significantly increased in the roots of Z31 or HC3 at the seed-filling stage compared with that in the rhizosphere of Z31 or HC3 (Fig 7). These findings are consistent with the results of 16S rDNA (V4 region) amplicon deep sequencing (Fig 6).

## Discussion

Considering the different opinions on the concept of root-associated microbiota by Edwards et al. [16] and Rascovan et al.[47], we provided an overall framework of the habitats related to this study. The components sequentially ordered from the roots inside to outside are the endosphere, rhizoplane, rhizosphere, surrounding soil, and bulk soil. Here, the root-associated microbial communities include three compartments, namely, the endosphere, rhizoplane, and rhizosphere [16]. The bulk soil as a blank control was collected from the field before sowing seeds. Surrounding soil is very loosely adhering to the plant roots and easily shaken off from the roots [8,48]. The rhizospheric soil is tightly adhering to the plant roots and collected by brushing off from root surface as previously described by Inceoglu et al. [35]. The rhizoplane is the microbial habitat at the root surface, which can be sonicated but cannot be washed off from the roots with PBS buffer [16]. The endosphere is the microbial habitat inside the plant root interior compartment [8,16]. In our study, we adopted bulk soil, surrounding soil, rhizospheric soil, and intact roots. The intact roots include the endosphere and the rhizoplane.

We observed that the insertion site of T-DNA and *g10-epsps* transgene in the transgenic soybean line Z31 is located in the chromosome No. 5 between two predicated open reading frames (ORF) (S13 Fig). We have a great interest to study the impacts of unexpected random insertion of transgene into some chromosomal noncoding regions on plant underground habitats (rhizosphere and endosphere), which is also related to genetic modified (GM) or transgenic crop biosafety evaluation in China. Thus, we initiated a project to evaluate the potential effects of the microbial communities in the root-associated soil habitats of the transgenic soybean line Z31, which has not been reported previously, as compared with its recipient cultivar HC3. We focused on the effect of the transgenic soybean line on the root-associated soil microbial communities and also would like to elucidate the mechanism underlying this effect.

Previous studies hypothesized that the horizontal gene transfer of transgenes, product of transgene expression [3], and unintentional changes in transgenic plant root exudates, including some chemical compositions [4], influences rhizosphere microbes. In the present study, we hypothesized that some other unexpected pleiotropic effects resulting from the integration of transgene into plant chromosome, such as the altered expression of flanking genes which encode small RNAs including microRNAs [49] or small interfering RNAs (siRNAs), influence rhizosphere microbes because small RNAs including some siRNAs move from host plant cells to the fungal pathogen [50,51].

Although the direct connection between this genetic modification and the effect on root-associated microbial communities remains unknown, we still cannot exclude some evident unexpected pleiotropic effects, such as the significant difference in beta diversity between the rhizosphere bacterial communities of Z31 and HC3 at the vegetative and seed-filling stages. Furthermore, we observed that the plant height and pod height of the transgenic line Z31 was lower than that of HC3, and the branch number of Z31 was greater than that of HC3 under water treatment. However, no significant differences in most of other agronomic traits, e.g., pitch number, pod number, seed number, yield per plant, hundred grain weight, and root architecture, were found between them.

Essentially, the detection limit of 16S rDNA amplicon deep sequencing-based detection is low reproducibility and quantitation, especially for beta-diversity [44,52]. Therefore, increasing sampling efforts (including sequencing efforts) and sample replicate number is the most effective way to improve technical reproducibility and quantitation [52]. In the present study, we adopted six sample replicates of different compartments of Z31 and HC3 and conducted 16S rDNA amplicon deep sequencing for most of these sample replicates, except for four sample replicates of bulk soil and roots at the flowering stage. Moreover, an average count per sample with more than 61,729 paired clean reads in this study was near the sequencing number for the desired 90% OTU overlap [52].

The increased reproducibility and quantitation in 16S rDNA amplicon deep sequencing in this study have been confirmed by previous studies [18,25,30]. The top one dominating phylum in the rhizospheric soil of soybean Z31 and HC3 in this study is Proteobacteria, which is consistent with the reports of Lu et al.[25] and Sugiyama et al.[30]. The top three dominating phyla in the rhizospheric soil of soybean Z31 and HC3 in this study are Proteobacteria, Acidobacteria, and Bacteroidetes, which are consistent with the results reported by Lu et al. [25] and by Liang et al. [18]. By contrast, Sujiyama et al. [30] showed that Actinobacteria is one of the top three dominating phyla in the rhizosphere soil of soybean Fukujishi at the growth stages (R2 and R6) and is one of top two dominating phyla in the bulk soil of their study. Similarly, Acidobacteria was one of top two dominating phyla in the bulk and surrounding soil samples of our study but was not one of the top three dominating phyla in the study by Sujiyama et al. [30]. Previous studies and our study confirmed again that soil type is the most major factor determining the composition and structure of bacterial communities in the bulk, surrounding and rhizospheric soil [8].

However, Bulgarelli et al. hypothesized plant species and/or genotype to be the major factor determining the composition and structure of bacterial communities in the two-step model for root microbiota differentiation [8]. Recently, Rascovan et al. have reported that the most abundant phylum in the root endophytes of soybean cultivar Wiliams 82 is Proteobacteria, wherein the most abundant class is Gammaproteobacteria, although only 1.5%–2.5% pyrosequencing reads were identified as Proteobacteria in the rhizospheric soil [47]. They hypothesized that PCR bias effect is not observed in root datasets because Proteobacteria highly dominate the root endophytes and are successfully amplified even with poor primer hybridization [47]. In the present study, Proteobacteria also highly dominated the soybean root endophytes at the reproductive stages (S25 and S26 Tables, sheet3) along with Cyanobacteria (chloroplast) (S26 Table, sheet 3). Furthermore, Gammaproteobacteria was the most abundant class with 25.534% ± 3.006% and 23.034% ± 3.164% relative abundances in the roots of Z31 and HC3, respectively, at the seed-filling stage except chloroplast (S31 Table, sheet 3). Meanwhile, Alphaproteobacteria was the dominating class with 31.064% ± 0.629% and 28.925% ± 0.793% relative abundances in the roots of Z31 and HC3 at the flowering stage (S30 Table, sheet 3). It was also the dominating class with 19.707% ± 1.474% and 21.198% ± 2.918% relative abundances in the roots of Z31 and HC3 at the seed-filling stage (S31 Table, sheet 3). The results of the present study and the above results reported by Rascovan et al. [47] confirmed the two-step model for root microbiota differentiation [8].

To date, only few studies have further revealed the root-associated (endosphere, rhizoplane, and rhizosphere) microbiota of crops under field growth conditions using NGS platform [16,47,53]. Nevertheless, the root-associated microbiota of model plants *Arabidopsis thaliana* and *Lotus japonicus* have been comprehensively analyzed recently [54–57]. To the best of published knowledge [3,32,33,47,57–62], the present work is one of the rare examples to address the effect of the GT transgenic crops on root-associated bacterial communities, especially root endophytes, by using the NGS platform.

Previous studies showed that the culturable root-endophytic bacterial community of the *EPSPS*-transgenic plant Quest canola has a lower diversity compared with the conventional Excel canola at the mid-flowering stage via three different methods, namely, fatty acid methyl ester profiles, community level physiological profiles, and/or terminal amplified ribosomal DNA restriction analysis profiles [21,63].

Recently, Lopes et al. have reported that the composition and diversity of culturable endophytic bacterial population differ between nontransgenic and transgenic soybean at the R5 growth stage, but they attributed the difference to the herbicide application in the GT soybean and its nontransgenic control. They also found differences in the abundance of endophytic bacterial communities between some soybean cultivars but not in the abundance between BRS 245 RR and its nontransgenic BRS 133.[62]

In this study, we observed no significant differences in the alpha and beta diversities of root bacterial communities between Z31 and HC3 under field growth. The differential results might be attributed to the use of soybean plants with water treatment and culture-independent amplicon deep sequencing in this study.

The rhizosphere bacterial community is affected by the development of plants, such as soybean [30] and *Arabidopsis* [31]. Root-associated bacterial communities, including root endophytes, are affected by rice development [16]. In the present study, the relative abundance of the dominating phylum Proteobacteria, especially the Gammaproteobacteria class, in the root endosphere of Z31 and HC3 was affected by soybean developmental change from the flowering stage (S25 Table, sheet 3) to the seed-filling stage (S26 Table, sheet 3).

Rhizobium–legume symbioses provide ammonia for plant growth through symbiotic nitrogen fixation in land ecosystems [13]. Therefore, this study also focused on the composition of the main nitrogen-fixing bacteria (Figs 4–7). Among the 15 main symbiotic nitrogen-fixing bacterial genera including *Agrobacterium* with legumes [64,65], five genera (i.e., *Azorhizobium*, *Ensifer* formerly *Sinorhizobium*, *Phyllobacterium*, *Microvirga*, and *Shinella*) were not found in this study. The relative abundances of most of the root-associated main symbiotic nitrogen-fixing bacteria were influenced by soybean developmental change, especially from the flowering stage to the seed-filling stage (Figs 5–7). Chaparro et al [31] reported that beneficial microbes are remarkably active during the late stage of *Arabidopsis* development. In addition, the relative abundances of *Bradyrhizobium* increased in the rhizospheric soils of HC3 and Z31 at the seed-filling stage compared with the flowering stage, which is also consistent with the previous results reported by Sugiyama et al. [30].

We were impressed by the highly relative abundance of the symbiotic diazotroph *Bradyrhizobium* in the roots of Z31and HC3 at the seed-filling stage (Fig 6) because it was one of the top two dominating genera (S29 Table, sheet 3). However, further research is needed to discover whether the relative abundance of the symbiotic diazotroph *Bradyrhizobium* in the roots of Z31 is statistically different from that of HC3 at the seed-filling stage. Moreover, identifying *Bradyrhizobium* species in detail using culture-dependent isolation methods is noteworthy.

## Conclusion

The *EPSPS*-transgenic soybean line Z31 exerted transitory effects on the taxonomic diversity of rhizosphere bacterial communities at the vegetative and seed-filling stages compared with the control under field conditions. In addition, soybean developmental change evidently influenced the main symbiotic nitrogen-fixing bacterial genera in the roots from the flowering stage to the seed-filling stage.

## Supporting information

S1 Fig Rarefaction curves of observed OTUs of the transgenic line Z31 and its recipient HC3 at the vegetative stage. HC3BRh and Z31BRh represent the rhizospheric soils of the recipient cultivar HC3 and the transgenic line Z31 at the vegetative stage, respectively. HC3BSO and Z31BSO represent the surrounding soils of the recipient cultivar HC3 and the transgenic line Z31 at the vegetative stage, respectively. HC3ASO and Z31ASO represent the bulk soils before sowing seeds of the recipient cultivar HC3 and the transgenic line Z31, respectively.
(TIF)

S2 Fig Rarefaction curves of observed OTUs of the transgenic line Z31 and its recipient HC3 at the flowering stage. HC3CRt and Z31CRt represent the roots of the recipient cultivar HC3 and the transgenic line Z31 at the flowering stage, respectively. HC3CRh and Z31CRh represent the rhizospheric soils of the recipient cultivar HC3 and the transgenic line Z31 at the flowering stage, respectively. HC3CSO and Z31CSO represent the surrounding soils of the recipient cultivar HC3 and the transgenic line Z31 at the flowering stage, respectively.
(TIF)

S3 Fig Rarefaction curves of observed OTUs of the transgenic line Z31 and its recipient HC3 at the seed-filling stage. HC3DRt and Z31DRt represent the roots of the recipient cultivar HC3 and the transgenic line Z31 at the seed-filling stage, respectively. HC3DRh and Z31DRh represent the rhizospheric soils of the recipient cultivar HC3 and the transgenic line Z31 at the seed-filling stage, respectively. HC3DSO and Z31DSO represent the surrounding soils of the recipient cultivar HC3 and the transgenic line Z31 at the seed-filling stage, respectively.
(TIF)

S4 Fig Accumulation curves of observed OTUs of the transgenic line Z31 and its recipient HC3 at the vegetative stage. (TIF)

S5 Fig Accumulation curves of observed OTUs of the transgenic line Z31 and its recipient HC3 at the flowering stage. (TIF)

S6 Fig Accumulation curves of observed OTUs of the transgenic line Z31 and its recipient HC3 at the seed-filling stage. (TIF)

S7 Fig Multivariate analyses of bacterial communities between Z31 and HC3 at the vegetative stage (n = 32). (A) Principal component analysis (PCA) based on operational taxonomic unit (OTU) abundance of bacterial communities. (B) PCoA based on the weighted UniFrac (WUF) distance metric. (C) Non-metric multidimensional scaling (NMDS) analysis based on the Bray–Curtis distance metrics. HC3BRh and Z31BRh represent the rhizospheric soils of the recipient cultivar HC3 and the transgenic line Z31 at the vegetative stage, respectively. HC3BSO and Z31BSO represent the surrounding soils of the recipient cultivar HC3 and the transgenic line Z31 at the vegetative stage, respectively. HC3ASO and Z31ASO represent the bulk soils before sowing seeds of the recipient cultivar HC3 and the transgenic line Z31, respectively.
(TIF)

S8 Fig Multivariate analyses of root-associated bacterial communities between Z31 and HC3 at the flowering stage (n = 32). (A) PCA based on OTU abundance of bacterial communities. (B) PCoA based on the WUF distance metric. (C) NMDS analysis based on the Bray–Curtis distance metrics. HC3CRt and Z31CRt represent the roots of the recipient cultivar HC3 and the transgenic line Z31 at the flowering stage, respectively. HC3CRh and Z31CRh represent the rhizospheric soils of the recipient cultivar HC3 and the transgenic line Z31 at the flowering stage, respectively. HC3CSO and Z31CSO represent the surrounding soils of the recipient cultivar HC3 and the transgenic line Z31 at the flowering stage, respectively.
(TIF)

S9 Fig Multivariate analyses of root-associated bacterial communities between Z31 and HC3 at the seed-filling stage (n = 36). (A) PCA based on OTU abundance of bacterial communities. (B) PCoA based on the WUF distance metric. (C) NMDS analysis based on the Bray–Curtis distance metrics. HC3DRt and Z31DRt represent the roots of the recipient cultivar HC3 and the transgenic line Z31 at the seed-filling stage, respectively. HC3DRh and Z31DRh represent the rhizospheric soils of the recipient cultivar HC3 and the transgenic line Z31 at the seed-filling stage, respectively. HC3DSO and Z31DSO represent the surrounding soils of the recipient cultivar HC3 and the transgenic line Z31 at the seed-filling stage, respectively.
(TIF)

S10 Fig Unweighted pair-group method with arithmetic mean (UPGMA) tree based on the WUF at the vegetative stage. (TIF)

S11 Fig UPGMA_tree based on the WUF at the flowering stage. (TIF)

S12 Fig UPGMA_tree based on the WUF at the seed-filling stage. (TIF)

S13 Fig Flanking sequence and insertion site of T-DNA and transgenes in the transgenic soybean line Z31. Capital letters represented sequences of T-DNA and transgenes including *g10-epsps*. Lowercase letters represented flanking sequences in the chromosome No. 5 of the soybean cultivar HC3, in which two predicted ORF labeled with red color were found. The sense primer with capital letters and the reverse complementary sequence of antisense primer, labeled by green color in the rectangular frame, were designed to confirm insertion site. Database: Gmax_275_v2.0. (https://soybase.org/GlycineBlastPages/).
(TIF)

S1 Table Summary of reads, tags and OTUs of bulk, surrounding and rhizospheric soils of Z31 and HC3 at the vegetative stage. (DOC)

S2 Table Normalized OTU table for biome of the bulk, surrounding and rhizospheric soils of Z31 and HC3 at the vegetative stage. (XLS)

S3 Table Summary of reads, tags and OTUs of the surrounding and rhizospheric soils and the roots of Z31 and HC3 at the flowering stage. (DOC)

S4 Table Normalized OTU table for biome of the surrounding and rhizospheric soils and the roots of Z31 and HC3 at the flowering stage. (XLS)

S5 Table Summary of reads, tags and OTUs of the surrounding and rhizospheric soils and the roots of Z31 and HC3 at the seed-filling stage. (DOC)

S6 Table Normalized OTU table for biome of the surrounding and rhizospheric soils and the roots of Z31 and HC3 at the seed-filling stage. (XLS)

S7 Table Analysis codes of 100 samples’ clean data. (DOCX)

S8 Table Comparison of alpha diversity between rhizospheric soils of Z31&HC3 at the vegetative stage. (DOC)

S9 Table Comparison of alpha diversity between rhizospheric soils and roots of Z31&HC3 at the flowering stage. (DOC)

S10 Table Comparison of alpha diversity between rhizospheric soils and roots of Z31&HC3 at the seed-filling stage. (DOC)

S11 Table Comparison of the alpha diversity between the bulk soil of Z31 and HC3 before sowing soybean seeds. (DOC)

S12 Table Comparison of the alpha diversity between the surrounding soils of Z31 and HC3 at the vegetative stage. (DOC)

S13 Table Comparison of the alpha diversity between the surrounding soils of Z31 and HC3 at the flowering stage. (DOC)

S14 Table Comparison of the alpha diversity between the surrounding soils of Z31 and HC3 at the seed-filling stage. (DOC)

S15 Table ADONIS analysis of the bulk, surrounding, and rhizospheric soil samples between Z31 and HC3 based on Bray-Curtis distance at vegetative stage. (DOC)

S16 Table Multiple response permutation procedure (MRPP) analysis of the bulk, surrounding, and rhizospheric soil samples between Z31&HC3 at vegetative stage. (DOC)

S17 Table Analysis of similarities (ANOSIM) analysis of the bulk, surrounding, and rhizospheric soil samples between Z31&HC3 at vegetative stage. (DOC)

S18 Table Multiple response permutation procedure (MRPP) analysis of the surrounding and rhizospheric soil samples and the root samples between Z31 and HC3 at the flowering stage. (DOC)

S19 Table Analysis of similarities (ANOSIM) of the surrounding and rhizospheric soil samples and the root samples between Z31 and HC3 at the flowering stage. (DOC)

S20 Table ADONIS analysis of the surrounding soil, rhizosphere soil and root samples between Z31 and HC3 based on Bray-Curtis distance metrics at the flowering stage. (DOC)

S21 Table MRPP analysis of the surrounding and rhizospheric soil samples and the root samples between Z31 and HC3 at the seed-filling stage. (DOC)

S22 Table ANOSIM of the surrounding and rhizosphere soil samples and the root samples between Z31 and HC3 at the seed-filling stage. (DOC)

S23 Table ADONIS analysis of the surrounding soil, rhizosphere soil and root samples between Z31 and HC3 based on Bray-Curtis distance metrics at the seed-filling stage. (DOC)

S24 Table Relative abundances of bacterial phyla at the vegetative stage. (XLS)

S25 Table Relative abundances of bacterial phyla at the flowering stage. (XLS)

S26 Table Relative abundances of bacterial phyla at the seed-filling stage. (XLS)

S27 Table Relative abundances of bacterial genera at the vegetative stage. (XLS)

S28 Table Relative abundances of bacterial genera at the flowering stage. (XLS)

S29 Table Relative abundances of bacterial genera at the seed-filling stage. (XLS)

S30 Table Relative abundances of bacterial classes at the flowering stage. (XLS)

S31 Table Relative abundances of bacterial classes at the seed-filling stage. (XLS)
