# Supplementary figures and images for: Effects of an EPSPS-transgenic soybean line ZUTS31 on root-associated bacterial communities during field growth
Source: PLoS One. 2018 Feb 6;13(2):e0192008. doi: 10.1371/journal.pone.0192008 (PMC5800644; doi:10.1371/journal.pone.0192008)

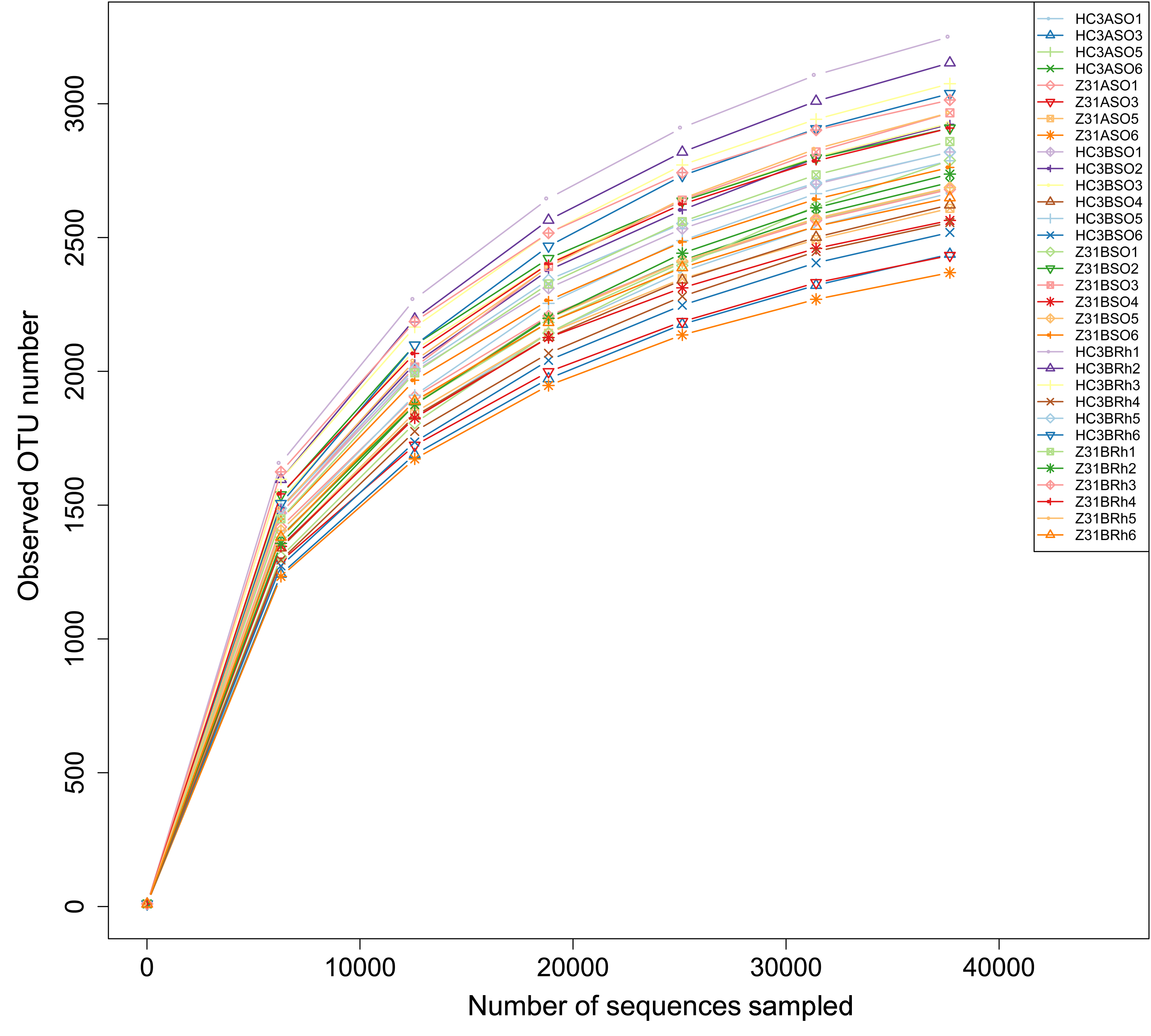

Supplement: S1 Fig — HC3BRh and Z31BRh represent the rhizospheric soils of the recipient cultivar HC3 and the transgenic line Z31 at the vegetative stage, respectively. HC3BSO and Z31BSO represent the surrounding soils of the recipient cultivar HC3 and the transgenic line Z31 at the vegetative stage, respectively. HC3ASO and Z31ASO represent the bulk soils before sowing seeds of the recipient cultivar HC3 and the transgenic line Z31, respectively. (TIF) [file pone.0192008.s001.tif]

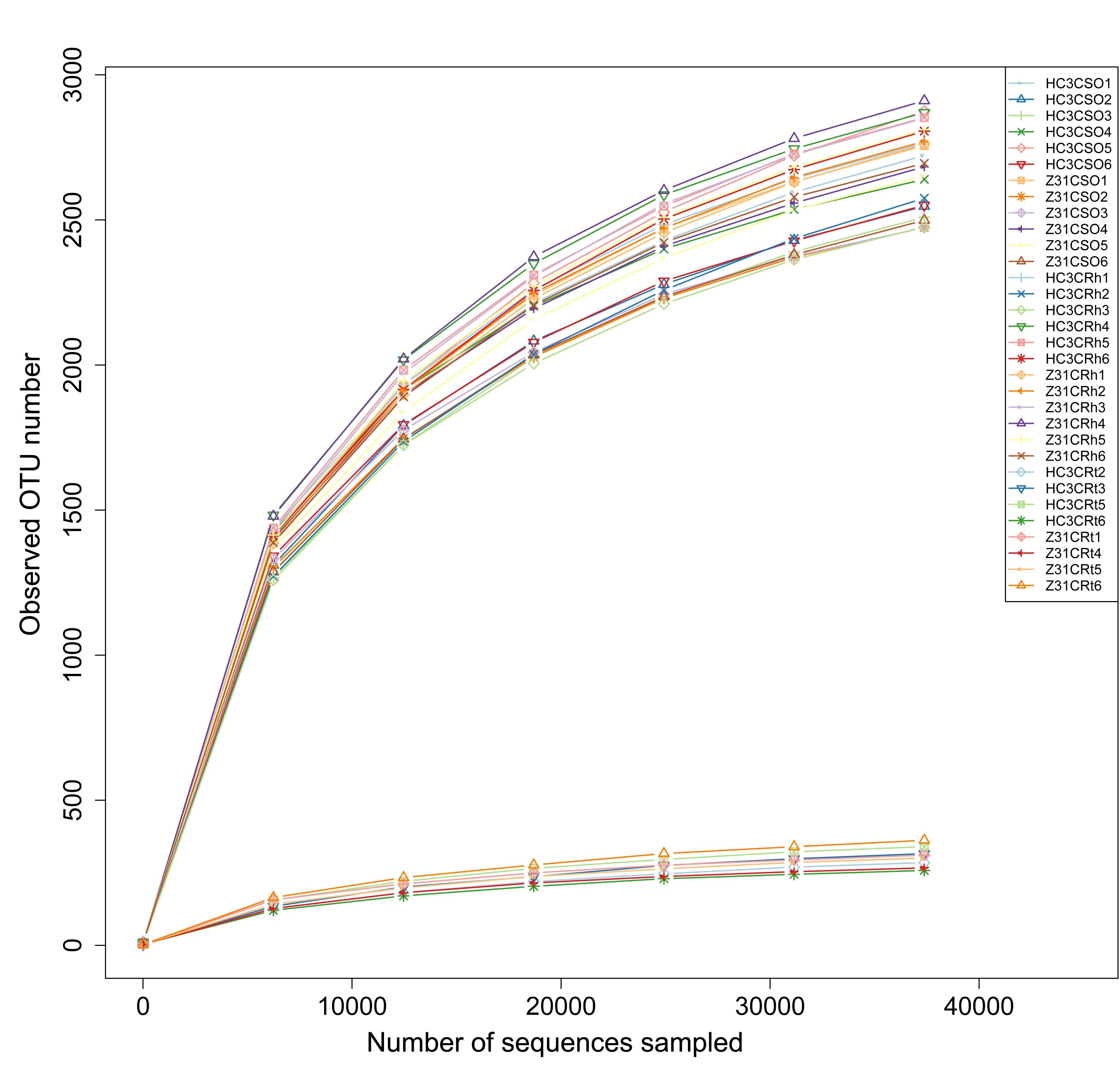

Supplement: S2 Fig — HC3CRt and Z31CRt represent the roots of the recipient cultivar HC3 and the transgenic line Z31 at the flowering stage, respectively. HC3CRh and Z31CRh represent the rhizospheric soils of the recipient cultivar HC3 and the transgenic line Z31 at the flowering stage, respectively. HC3CSO and Z31CSO represent the surrounding soils of the recipient cultivar HC3 and the transgenic line Z31 at the flowering stage, respectively. (TIF) [file pone.0192008.s002.tif]

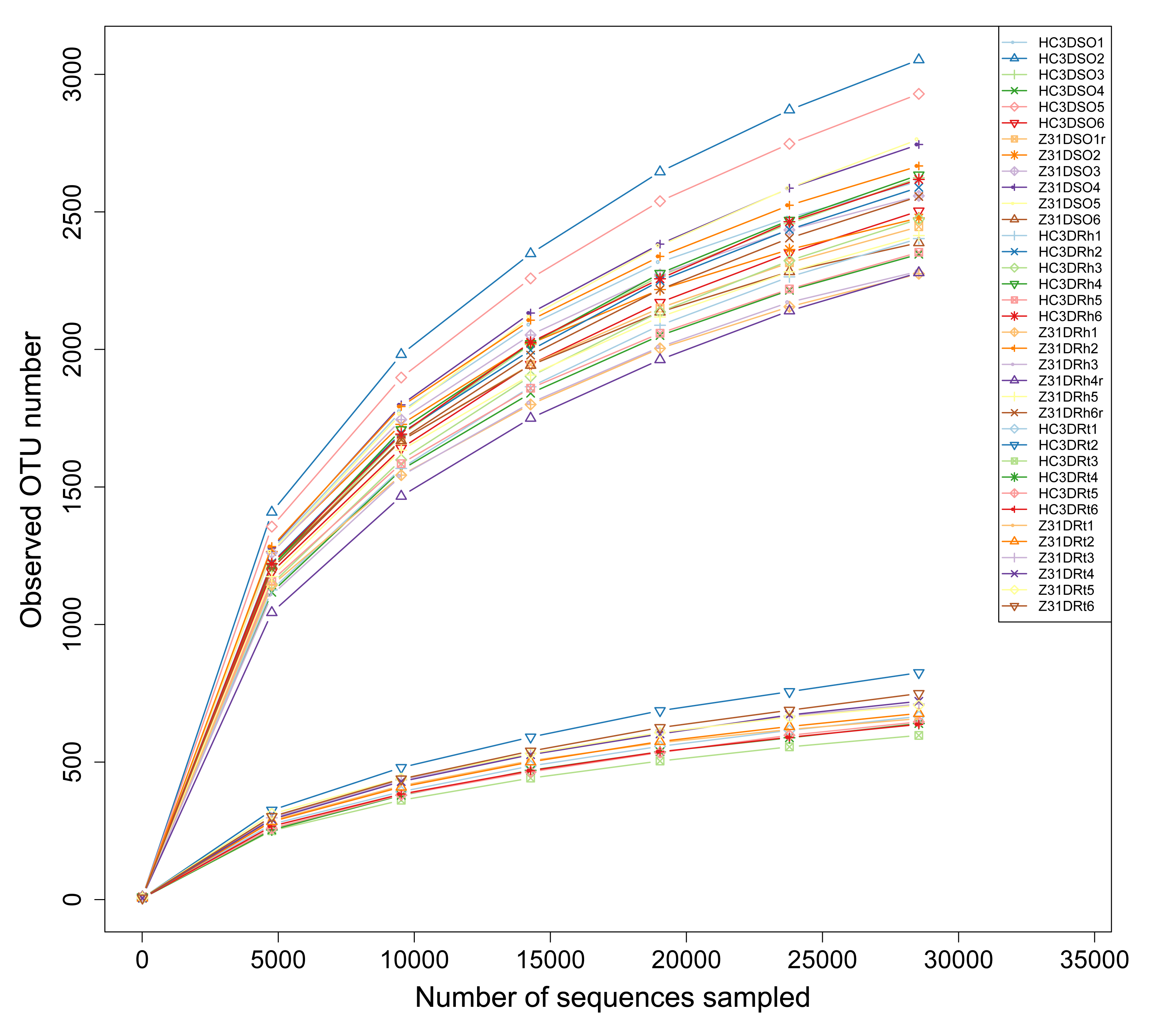

Supplement: S3 Fig — HC3DRt and Z31DRt represent the roots of the recipient cultivar HC3 and the transgenic line Z31 at the seed-filling stage, respectively. HC3DRh and Z31DRh represent the rhizospheric soils of the recipient cultivar HC3 and the transgenic line Z31 at the seed-filling stage, respectively. HC3DSO and Z31DSO represent the surrounding soils of the recipient cultivar HC3 and the transgenic line Z31 at the seed-filling stage, respectively. (TIF) [file pone.0192008.s003.tif]

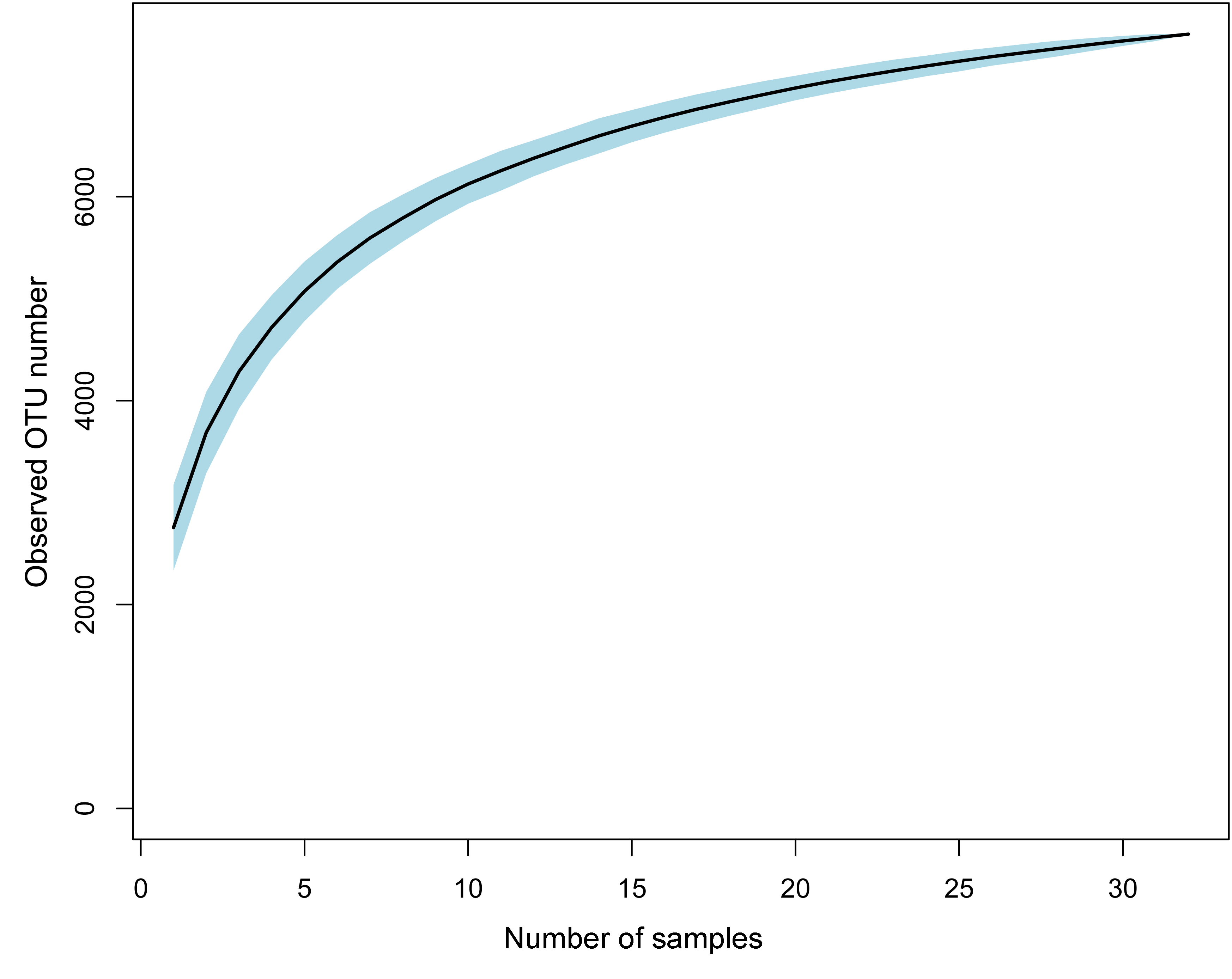

Supplement: S4 Fig — (TIF) [file pone.0192008.s004.tif]

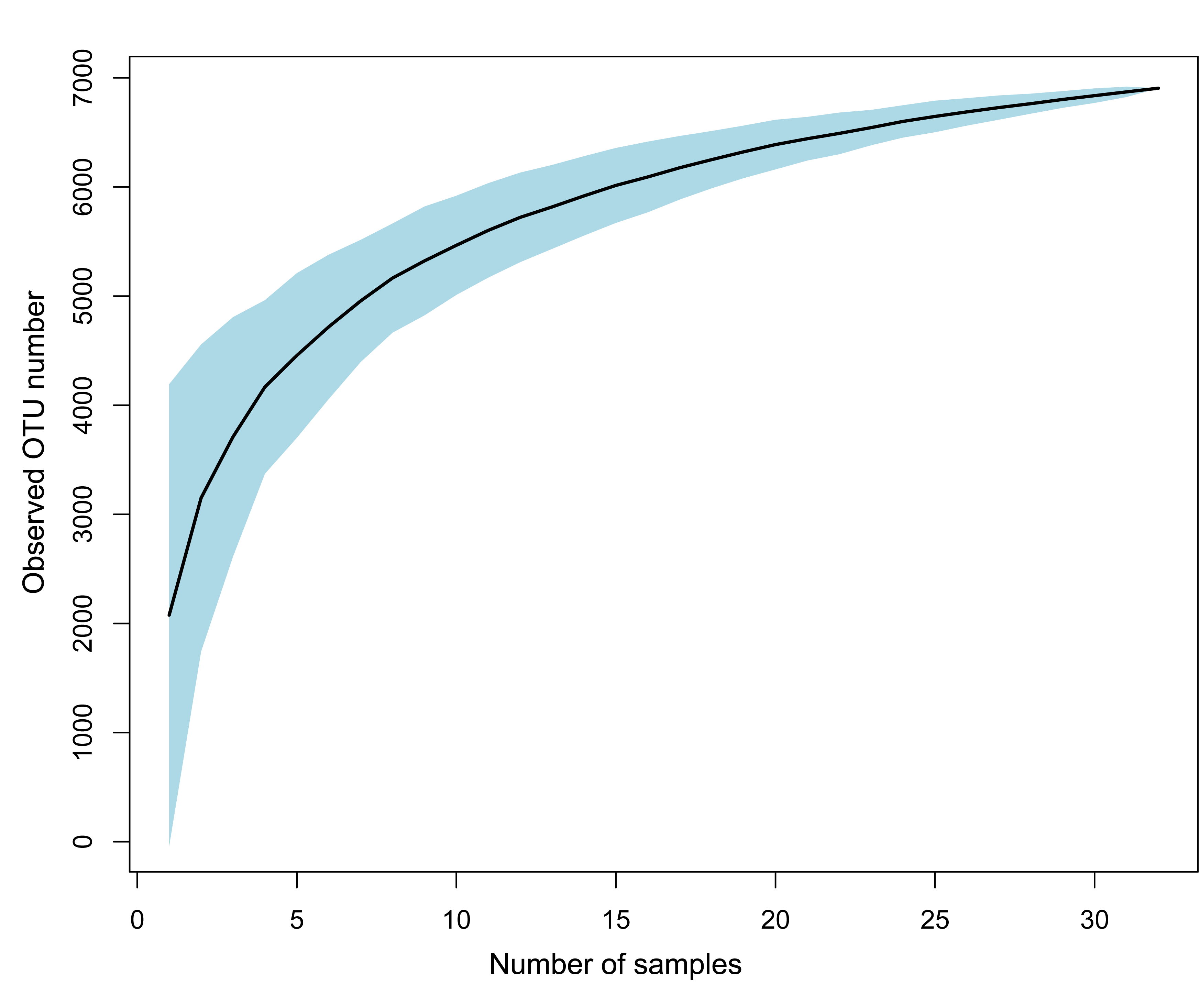

Supplement: S5 Fig — (TIF) [file pone.0192008.s005.tif]

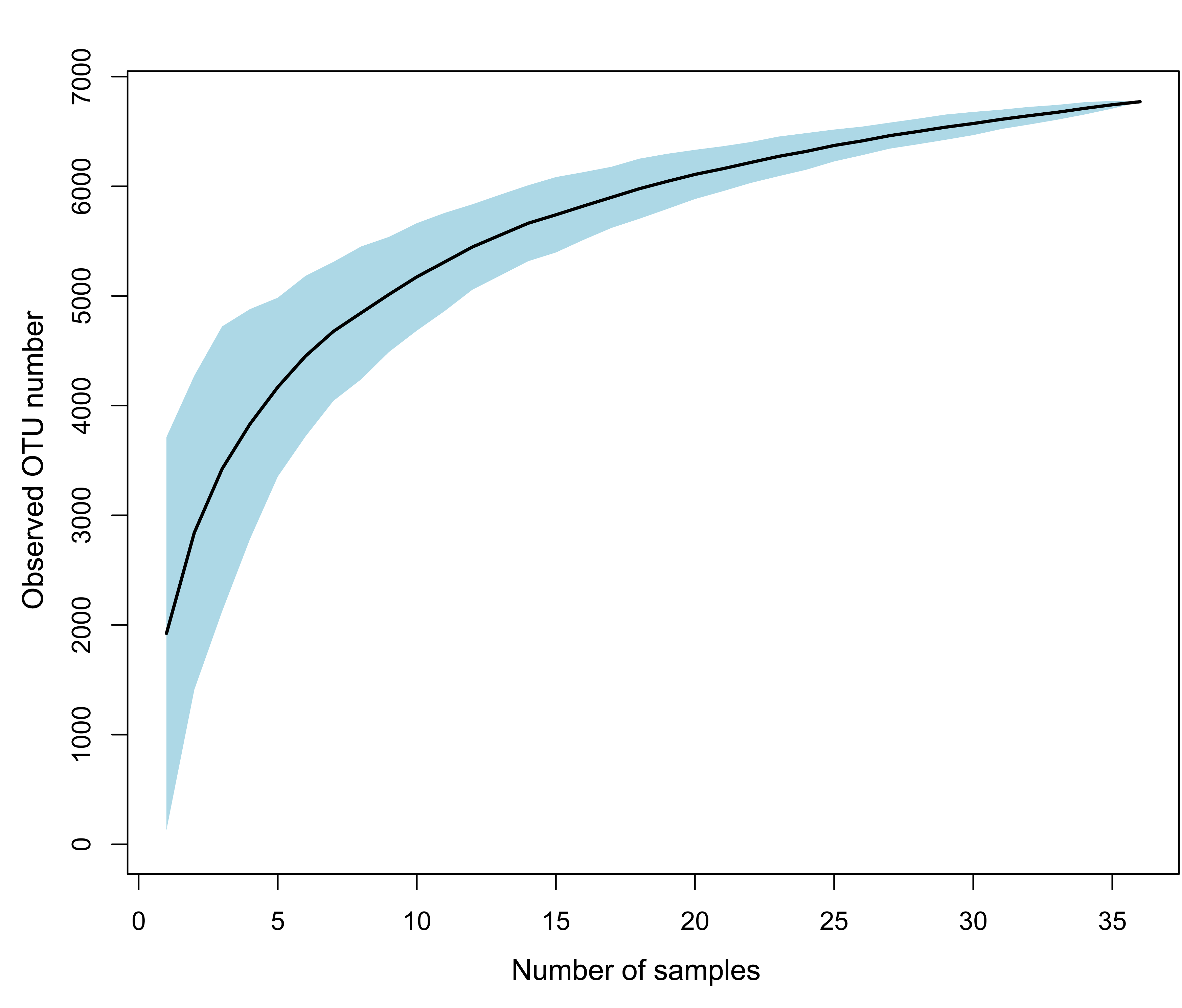

Supplement: S6 Fig — (TIF) [file pone.0192008.s006.tif]

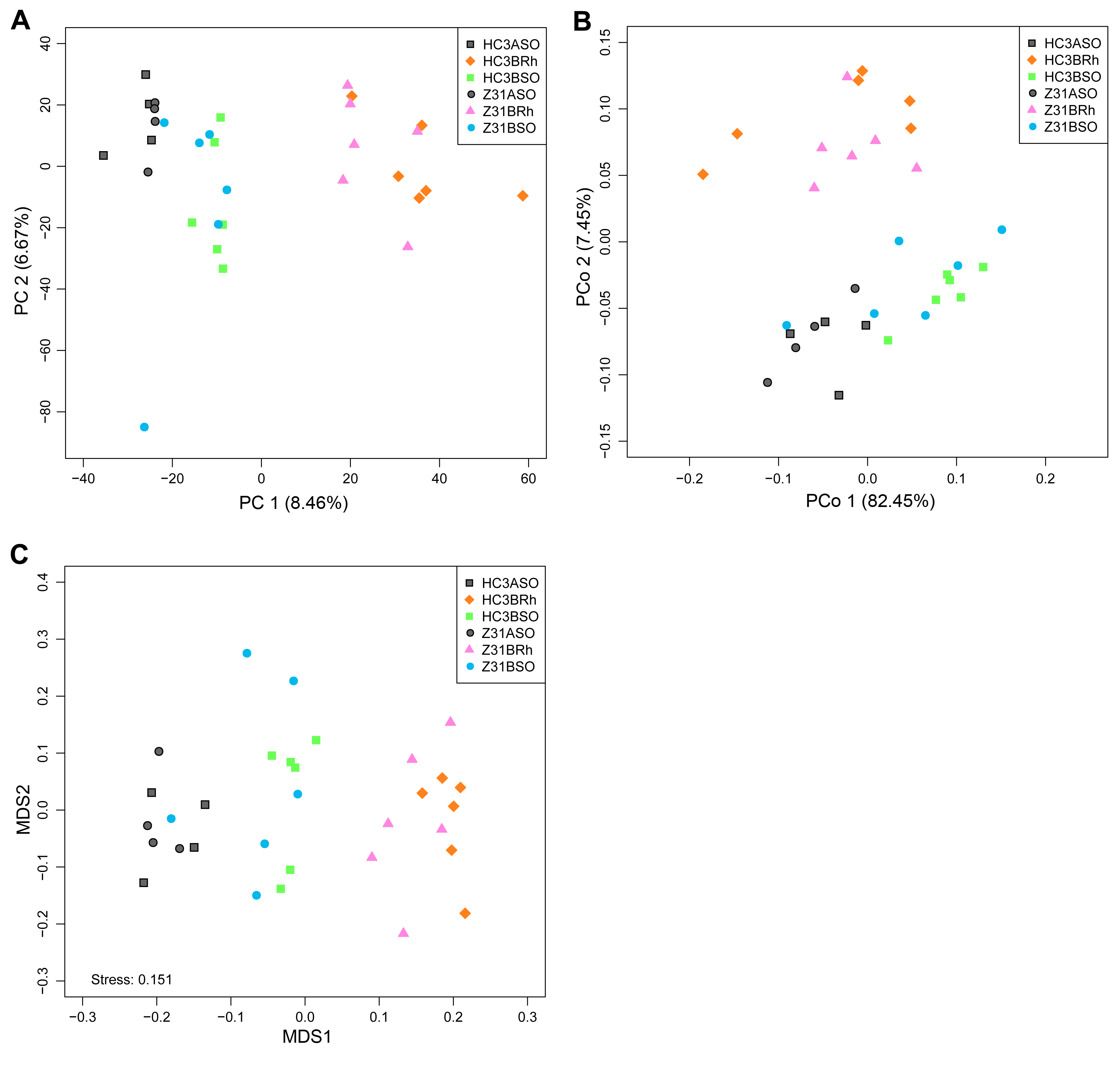

Supplement: S7 Fig — (A) Principal component analysis (PCA) based on operational taxonomic unit (OTU) abundance of bacterial communities. (B) PCoA based on the weighted UniFrac (WUF) distance metric. (C) Non-metric multidimensional scaling (NMDS) analysis based on the Bray–Curtis distance metrics. HC3BRh and Z31BRh represent the rhizospheric soils of the recipient cultivar HC3 and the transgenic line Z31 at the vegetative stage, respectively. HC3BSO and Z31BSO represent the surrounding soils of the recipient cultivar HC3 and the transgenic line Z31 at the vegetative stage, respectively. HC3ASO and Z31ASO represent the bulk soils before sowing seeds of the recipient cultivar HC3 and the transgenic line Z31, respectively. (TIF) [file pone.0192008.s007.tif]

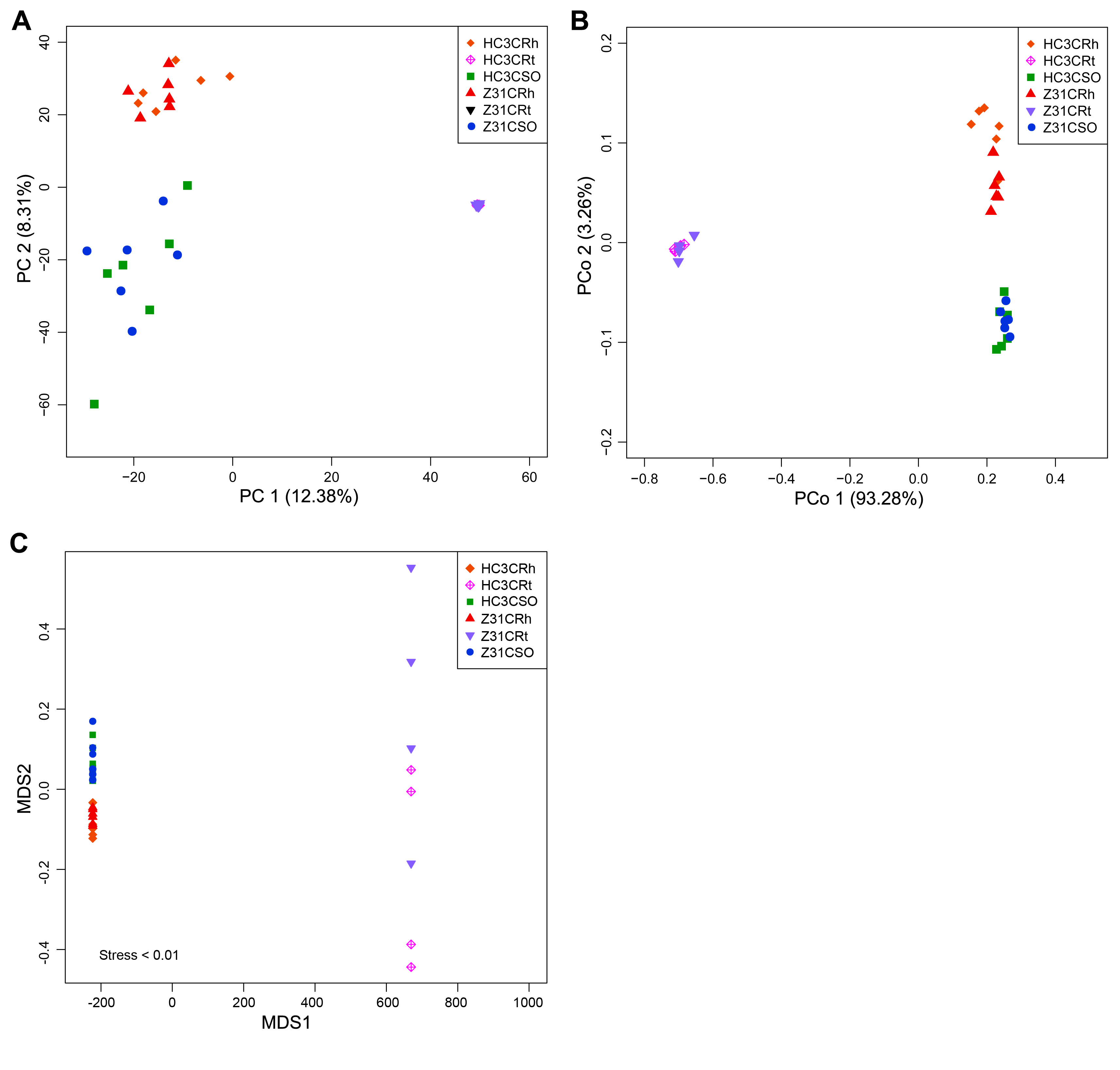

Supplement: S8 Fig — (A) PCA based on OTU abundance of bacterial communities. (B) PCoA based on the WUF distance metric. (C) NMDS analysis based on the Bray–Curtis distance metrics. HC3CRt and Z31CRt represent the roots of the recipient cultivar HC3 and the transgenic line Z31 at the flowering stage, respectively. HC3CRh and Z31CRh represent the rhizospheric soils of the recipient cultivar HC3 and the transgenic line Z31 at the flowering stage, respectively. HC3CSO and Z31CSO represent the surrounding soils of the recipient cultivar HC3 and the transgenic line Z31 at the flowering stage, respectively. (TIF) [file pone.0192008.s008.tif]

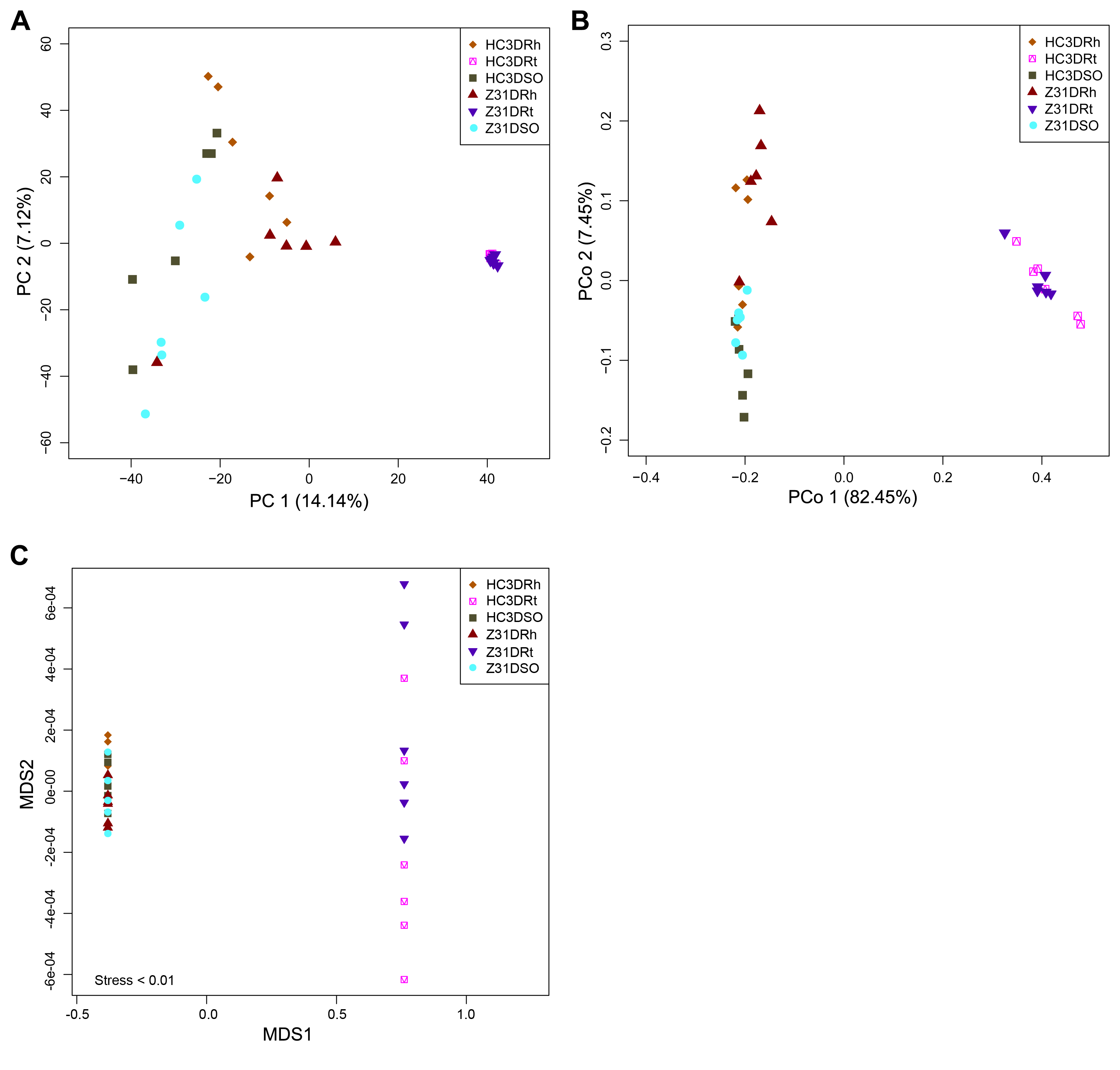

Supplement: S9 Fig — (A) PCA based on OTU abundance of bacterial communities. (B) PCoA based on the WUF distance metric. (C) NMDS analysis based on the Bray–Curtis distance metrics. HC3DRt and Z31DRt represent the roots of the recipient cultivar HC3 and the transgenic line Z31 at the seed-filling stage, respectively. HC3DRh and Z31DRh represent the rhizospheric soils of the recipient cultivar HC3 and the transgenic line Z31 at the seed-filling stage, respectively. HC3DSO and Z31DSO represent the surrounding soils of the recipient cultivar HC3 and the transgenic line Z31 at the seed-filling stage, respectively. (TIF) [file pone.0192008.s009.tif]

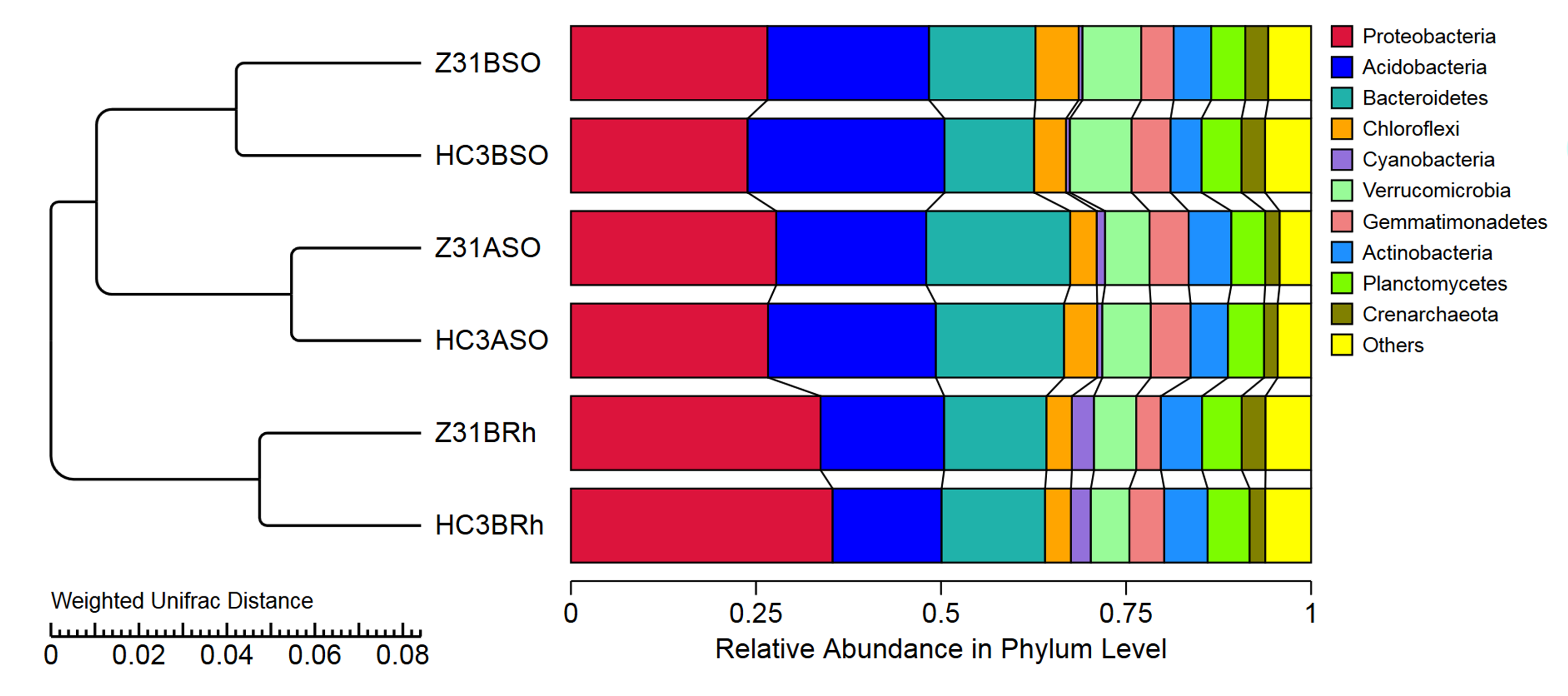

Supplement: S10 Fig — (TIF) [file pone.0192008.s010.tif]

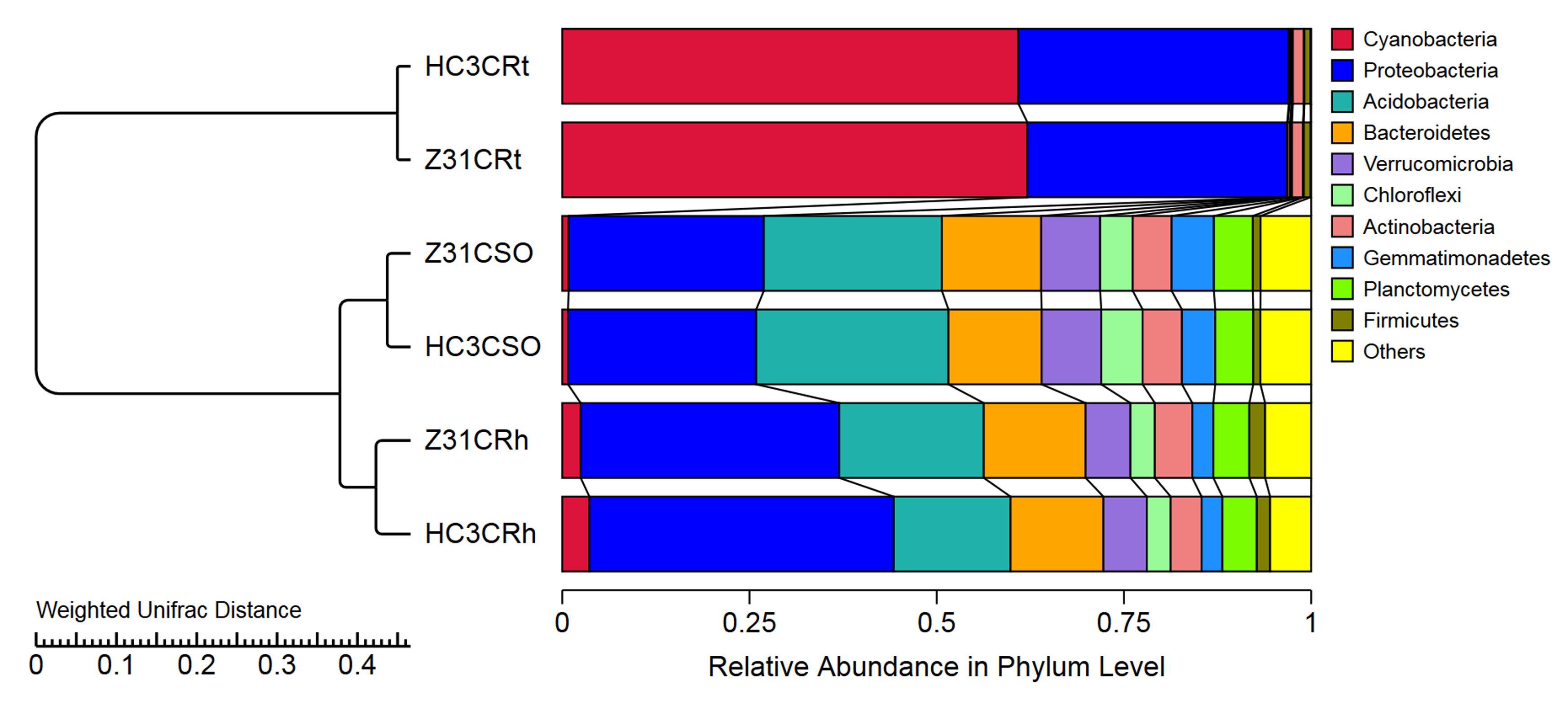

Supplement: S11 Fig — (TIF) [file pone.0192008.s011.tif]

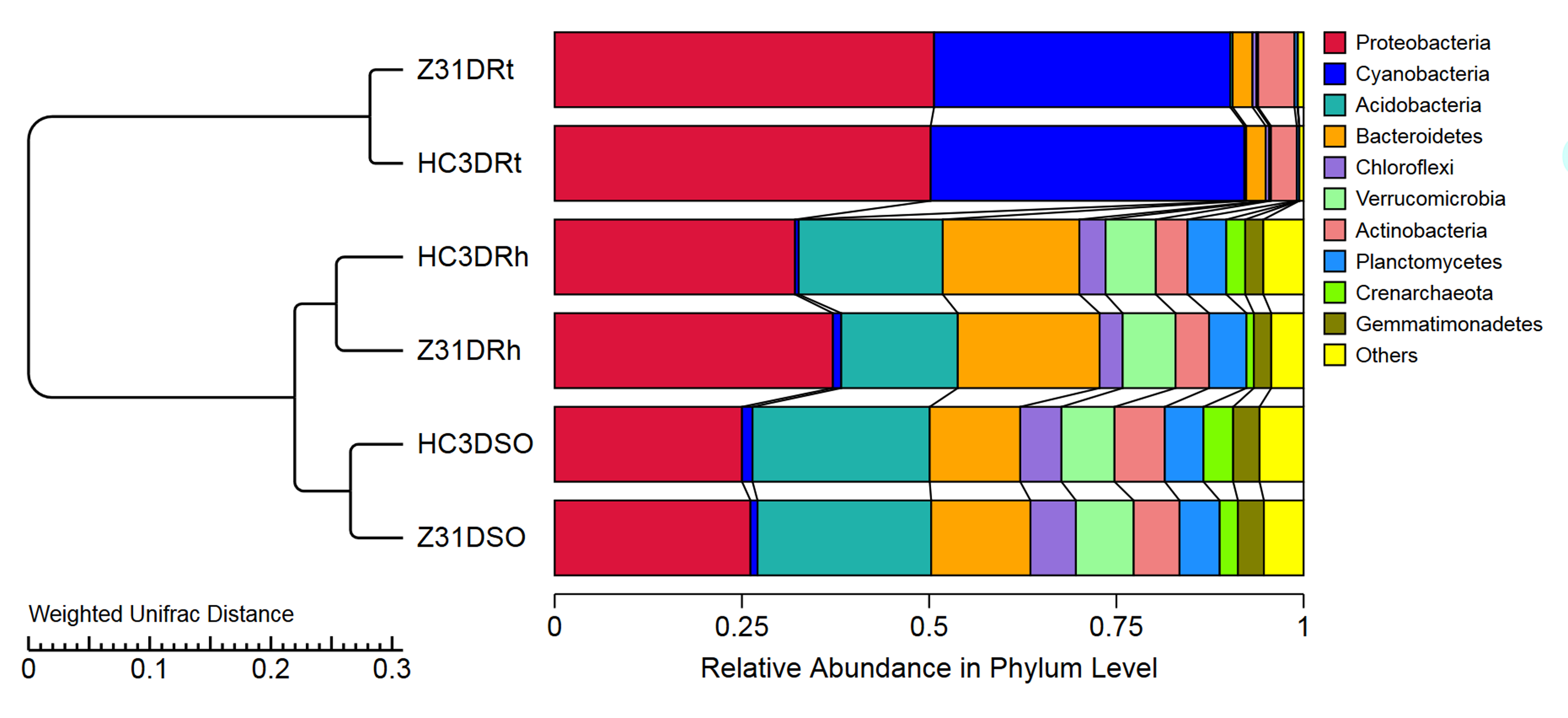

Supplement: S12 Fig — (TIF) [file pone.0192008.s012.tif]

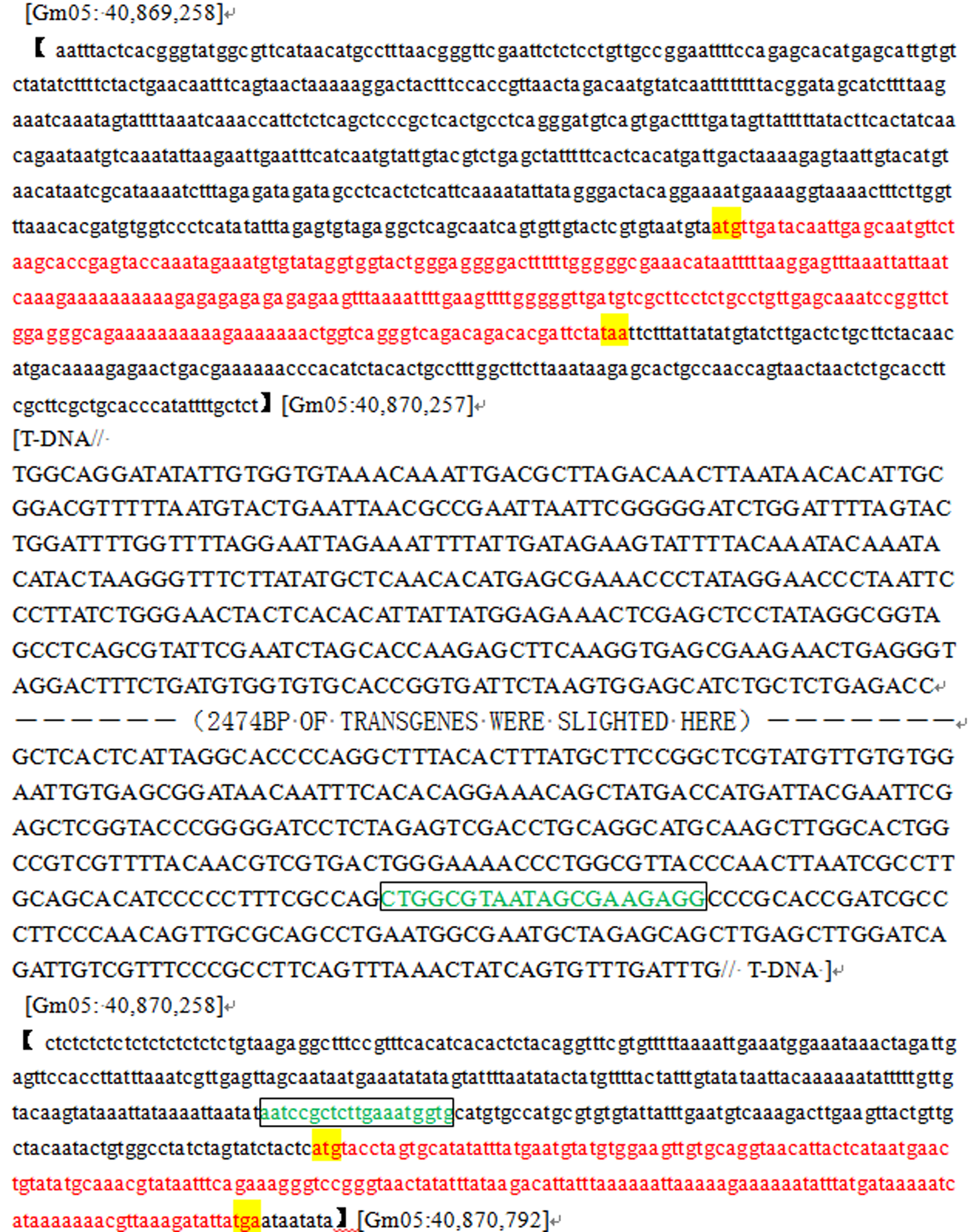

Supplement: S13 Fig — Capital letters represented sequences of T-DNA and transgenes including g10-epsps. Lowercase letters represented flanking sequences in the chromosome No. 5 of the soybean cultivar HC3, in which two predicted ORF labeled with red color were found. The sense primer with capital letters and the reverse complementary sequence of antisense primer, labeled by green color in the rectangular frame, were designed to confirm insertion site. Database: Gmax_275_v2.0. (https://soybase.org/GlycineBlastPages/). (TIF) [file pone.0192008.s013.tif]
