# Supplementary material for: Effects of an EPSPS-transgenic soybean line ZUTS31 on root-associated bacterial communities during field growth
Source: PLoS One. 2018 Feb 6;13(2):e0192008. doi: 10.1371/journal.pone.0192008 (PMC5800644; doi:10.1371/journal.pone.0192008)
Supplement: S1 Table — (DOC) [file pone.0192008.s014.doc]

**S1 Table. Summary of reads, tags and OTUs of bulk soils, surrounding soils, and rhizospheric soils of the transgenic soybean line Z31 and its recipient cultivar HC3 at the vegetative stage.**

| Sample name | Clean reads  (250 nt) (paired-end) | Clean Tags | Effective Tags | Q30 of effective Tags (%) | Taxonomic Tags at 97% similarity | OTUs |
| --- | --- | --- | --- | --- | --- | --- |
| HC3ASO1 | 69,215 × 2 | 66,231 | 60,816 | 98.36 | 50,391 | 3178 |
| HC3ASO3 | 60,752 × 2 | 58,420 | 53,731 | 98.24 | 45,009 | 2850 |
| HC3ASO5 | 61,493 × 2 | 59,233 | 55,215 | 98.28 | 44,981 | 3149 |
| HC3ASO6 | 69,210 × 2 | 66,456 | 61,242 | 98.31 | 49,515 | 3214 |
| Z31ASO1 | 59,751 × 2 | 57,582 | 53,230 | 98.33 | 44,082 | 3075 |
| Z31ASO3 | 55,843 × 2 | 53,364 | 48,573 | 98.32 | 39,868 | 2859 |
| Z31ASO5 | 55,983 × 2 | 53,429 | 49,009 | 98.40 | 40,777 | 3011 |
| Z31ASO6 | 56,261 × 2 | 53,736 | 49,797 | 98.35 | 42,055 | 2780 |
| HC3BSO1 | 57,350 × 2 | 55,335 | 51,841 | 98.45 | 41,057 | 3280 |
| HC3BSO2 | 63,843 × 2 | 61,752 | 58,092 | 98.41 | 45,839 | 3400 |
| HC3BSO3 | 59,349 × 2 | 57,351 | 53,827 | 98.45 | 42,720 | 3399 |
| HC3BSO4 | 68,573 × 2 | 66,399 | 62,047 | 98.47 | 50,782 | 3091 |
| HC3BSO5 | 65,443 × 2 | 63,339 | 59,463 | 98.42 | 47,329 | 3312 |
| HC3BSO6 | 63,366 × 2 | 61,164 | 56,651 | 98.40 | 45,931 | 2982 |
| Z31BSO1 | 51,568 × 2 | 49,820 | 45,969 | 98.39 | **37,694** | 2788 |
| Z31BSO2 | 58,361 × 2 | 56,358 | 53,364 | 98.42 | 41,062 | 3336 |
| Z31BSO3 | 66,143 × 2 | 63,871 | 59,649 | 98.44 | 48,284 | 3496 |
| Z31BSO4 | 56,138 × 2 | 54,249 | 49,640 | 98.39 | 40,486 | 2972 |
| Z31BSO5 | 61,056 × 2 | 59,119 | 53,500 | 98.43 | 43,131 | 3129 |
| Z31BSO6 | 63,535 × 2 | 61,741 | 56,944 | 98.50 | 45,227 | 3197 |
| HC3BRh1 | 68,222 × 2 | 65,937 | 61,045 | 98.60 | 48,383 | 3716 |
| HC3BRh2 | 66,273 × 2 | 63,989 | 58,213 | 98.57 | 46,747 | 3608 |
| HC3BRh3 | 61,950 × 2 | 59,819 | 54,944 | 98.64 | 43,674 | 3459 |
| HC3BRh4 | 68,938 × 2 | 66,307 | 59,917 | 98.58 | 50,479 | 2994 |
| HC3BRh5 | 64,545 × 2 | 62,218 | 56,654 | 98.52 | 44,930 | 3207 |
| HC3BRh6 | 64,274 × 2 | 61,686 | 55,707 | 98.54 | 46,645 | 3518 |
| Z31BRh1 | 71,521 × 2 | 68,919 | 62,661 | 98.56 | 50,707 | 3302 |
| Z31BRh2 | 72,732 × 2 | 69,714 | 61,591 | 98.60 | 50,206 | 3195 |
| Z31BRh3 | 55,843 × 2 | 54,045 | 50,231 | 98.53 | 38,492 | 3408 |
| Z31BRh4 | 63,289 × 2 | 61,168 | 55,447 | 98.55 | 44,413 | 3321 |
| Z31BRh5 | 70,488 × 2 | 68,053 | 61,919 | 98.59 | 49,156 | 3428 |
| Z31BRh6 | 56,540 × 2 | 54,627 | 49,372 | 98.47 | 39,737 | 3017 |

1. Clean Tags were obtained after connected tags were filtered to eliminate low quality and short sequence.
2. Effective Tags were obtained after clean tags were filtered to remove chimeras.
3. Four samples collected from six sampling points within three replicates/plots of bulk soils for planting the transgenic soybean line Z31 or its recipient cultivar HC3 were named as Z31ASO1, 3, 5, 6 or HC3ASO1, 3, 5, 6, respectively.
4. Six samples collected from six sampling points within three replicates/plots of surrounding soils of the transgenic soybean line Z31 or its recipient cultivar HC3 at seedling stage were named as Z31BSO1 ~ 6 or HC3BSO1 ~ 6, respectively.
5. Six samples collected from six sampling points within three replicates/plots of rhizosphere soils of the transgenic soybean line Z31 or its recipient cultivar HC3 at seedling stage were named as Z31BRh1 ~ 6 or HC3BRh1 ~ 6, respectively.
