# Supplementary material for: Effects of an EPSPS-transgenic soybean line ZUTS31 on root-associated bacterial communities during field growth
Source: PLoS One. 2018 Feb 6;13(2):e0192008. doi: 10.1371/journal.pone.0192008 (PMC5800644; doi:10.1371/journal.pone.0192008)
Supplement: S3 Table — (DOC) [file pone.0192008.s016.doc]

**S3 Table. Summary of reads, tags and OTUs of surrounding soils, rhizospheric soils, and roots of soybean transgenic line Z31 and its recipient cultivar HC3 at the flowering stage.**

| Sample name | Clean reads  (250 nt) (paired-end) | Clean Tags | Effective Tags | Q30 of effective Tags (%) | Taxonomic Tags at 97% similarity | OTUs |
| --- | --- | --- | --- | --- | --- | --- |
| HC3CSO1 | 66,675× 2 | 63,959 | 59,799 | 98.3 | 48087 | 3169 |
| HC3CSO2 | 63,724× 2 | 60,957 | 56,475 | 98.34 | 45576 | 2988 |
| HC3CSO3 | 69,752× 2 | 67,064 | 61,874 | 98.29 | 51252 | 2953 |
| HC3CSO4 | 55,813× 2 | 53,255 | 49,733 | 98.32 | 38903 | 3054 |
| HC3CSO5 | 79,566× 2 | 76,571 | 71,889 | 98.38 | 57512 | 3440 |
| HC3CSO6 | 57,347× 2 | 54,952 | 51,141 | 98.31 | 41368 | 2934 |
| Z31CSO1 | 70,883× 2 | 68,506 | 64,343 | 98.33 | 51510 | 3225 |
| Z31CSO2 | 68,007× 2 | 65,519 | 61,108 | 98.26 | 49220 | 2926 |
| Z31CSO3 | 58,326× 2 | 55,937 | 51,687 | 98.39 | 40767 | 2833 |
| Z31CSO4 | 61,463× 2 | 59,127 | 55,216 | 98.24 | 44598 | 3132 |
| Z31CSO5 | 69,234× 2 | 66,700 | 61,188 | 98.3 | 48464 | 3118 |
| Z31CSO6 | 69,323× 2 | 66,920 | 61,303 | 98.34 | 49777 | 2976 |
| HC3CRh1 | 64,545× 2 | 62,380 | 57,098 | 98.68 | 46307 | 3187 |
| HC3CRh2 | 79,542× 2 | 76,658 | 67,984 | 98.59 | 56533 | 3011 |
| HC3CRh3 | 65,882× 2 | 63,532 | 57,405 | 98.62 | 48060 | 2846 |
| HC3CRh4 | 64,645× 2 | 62,195 | 55,504 | 98.56 | 44838 | 3269 |
| HC3CRh5 | 68,934× 2 | 66,519 | 59,568 | 98.63 | 47678 | 3317 |
| HC3CRh6 | 75,741× 2 | 73,030 | 66,352 | 98.64 | 53602 | 3248 |
| Z31CRh1 | 74,421× 2 | 72,144 | 65,289 | 98.63 | 52538 | 3189 |
| Z31CRh2 | 71,050× 2 | 68,861 | 62,337 | 98.63 | 51004 | 3217 |
| Z31CRh3 | 71,310× 2 | 69,028 | 61,153 | 98.69 | 49614 | 3367 |
| Z31CRh4 | 65,477× 2 | 63,411 | 58,039 | 98.59 | 47182 | 3336 |
| Z31CRh5 | 70,757× 2 | 68,592 | 62,993 | 98.64 | 51140 | 3264 |
| Z31CRh6 | 66,934× 2 | 64,694 | 58,608 | 98.57 | 48103 | 3133 |
| HC3CRt2 | 49,184× 2 | 47,905 | 46,806 | 98.59 | 46391 | 343 |
| HC3CRt3 | 48,262× 2 | 47,073 | 45,912 | 98.48 | 45438 | 375 |
| HC3CRt5 | 40,900× 2 | 39,940 | 38,955 | 98.68 | 38531 | 420 |
| HC3CRt6 | 40,052× 2 | 39,171 | 37,825 | 98.7 | **37383** | 319 |
| Z31CRt1 | 41,328× 2 | 40,290 | 38,412 | 98.66 | 37836 | 372 |
| Z31CRt4 | 41,685× 2 | 40,733 | 39,503 | 98.69 | 39022 | 341 |
| Z31CRt5 | 43,194× 2 | 42,214 | 41,030 | 98.75 | 40589 | 369 |
| Z31CRt6 | 41,382× 2 | 40,520 | 39,561 | 98.73 | 39108 | 423 |

1. Clean Tags were obtained after connected tags were filtered to eliminate low quality and short sequence.
2. Effective Tags were obtained after clean tags were filtered to remove chimeras.
3. Six samples collected from six sampling points within three replicates/plots of surrounding soils of the transgenic soybean line Z31 or its recipient cultivar HC3 at flowering stage are named as Z31CSO1 ~ 6 or HC3CSO1 ~ 6, respectively.
4. Six samples collected from six sampling points within three replicates/plots of rhizosphere soils of the transgenic soybean line Z31 or its recipient cultivar HC3 at flowering stage are named as Z31CRh1 ~ 6 or HC3CRh1 ~ 6, respectively.
5. Four samples collected from six sampling points within three replicates/plots of root endosphere of the transgenic soybean line Z31 or its recipient cultivar HC3 at flowering stage are named as Z31CRt2, 3, 5, 6 or HC3CRt1, 4, 5, 6, respectively.
