# Supplementary material for: Effects of an EPSPS-transgenic soybean line ZUTS31 on root-associated bacterial communities during field growth
Source: PLoS One. 2018 Feb 6;13(2):e0192008. doi: 10.1371/journal.pone.0192008 (PMC5800644; doi:10.1371/journal.pone.0192008)
Supplement: S5 Table — (DOC) [file pone.0192008.s018.doc]

**S5 Table. Summary of reads, tags and OTUs of surrounding soils, rhizospheric soils, and roots of soybean transgenic line Z31 and its recipient cultivar HC3 at the seed-filling stage.**

| Sample name | Clean reads  (250 nt) (paired-end) | Clean Tags | Effective Tags | Q30 of effective Tags (%) | Taxonomic Tags at 97% similarity | OTUs |
| --- | --- | --- | --- | --- | --- | --- |
| HC3DSO1 | 48,647 × 2 | 47,075 | 43,384 | 98.4 | 35761 | 3054 |
| HC3DSO2 | 72,589 × 2 | 69,630 | 63,594 | 98.4 | 51123 | 3692 |
| HC3DSO3 | 76,609 × 2 | 73,660 | 67,229 | 98.47 | 55277 | 3243 |
| HC3DSO4 | 57,262 × 2 | 55,477 | 51,570 | 98.46 | 42968 | 2817 |
| HC3DSO5 | 68,519 × 2 | 65,602 | 60,922 | 98.37 | 49209 | 3537 |
| HC3DSO6 | 66,625 × 2 | 63,925 | 58,856 | 98.33 | 47609 | 3046 |
| Z31DSO1r | 77,366 × 2 | 74,706 | 70,211 | 98.63 | 57110 | 3093 |
| Z31DSO2 | 41,712 × 2 | 40,342 | 37,632 | 98.35 | 30414 | 2849 |
| Z31DSO3 | 52,754 × 2 | 49,614 | 44,894 | 98.33 | 36812 | 3013 |
| Z31DSO4 | 68,205 × 2 | 65,519 | 60,720 | 98.36 | 49461 | 3345 |
| Z31DSO5 | 38,447 × 2 | 37,136 | 34,522 | 98.35 | **28540** | 2764 |
| Z31DSO6 | 42,677 × 2 | 41,239 | 38,147 | 98.3 | 31971 | 2789 |
| HC3DRh1 | 69,057 × 2 | 66,948 | 60,486 | 98.71 | 50218 | 2925 |
| HC3DRh2 | 68,533 × 2 | 66,319 | 60,160 | 98.66 | 49352 | 3092 |
| HC3DRh3 | 70,047 × 2 | 68,180 | 62,845 | 98.73 | 51323 | 2944 |
| HC3DRh4 | 71,163 × 2 | 69,053 | 63,306 | 98.74 | 51848 | 3144 |
| HC3DRh5 | 67,255 × 2 | 65,187 | 58,917 | 98.57 | 47059 | 2779 |
| HC3DRh6 | 69,066 × 2 | 67,007 | 61,124 | 98.57 | 49411 | 3133 |
| Z31DRh1 | 63,081 × 2 | 61,177 | 55,021 | 98.68 | 45217 | 2653 |
| Z31DRh2 | 63,540 × 2 | 61,628 | 57,527 | 98.56 | 45726 | 3140 |
| Z31DRh3 | 57,961 × 2 | 56,182 | 50,911 | 98.61 | 42356 | 2690 |
| Z31DRh4r | 125,335 × 2 | 121,076 | 108,493 | 98.73 | 93859 | 3315 |
| Z31DRh5 | 60,644 × 2 | 58,834 | 53,074 | 98.6 | 44058 | 2837 |
| Z31DRh6r | 129,173 × 2 | 124,964 | 113,090 | 98.66 | 95072 | 3593 |
| HC3DRt1 | 68,398 × 2 | 66,173 | 57,789 | 98.64 | 56335 | 881 |
| HC3DRt2 | 63,998 × 2 | 61,705 | 53,449 | 98.51 | 51411 | 1079 |
| HC3DRt3 | 71,637 × 2 | 69,277 | 61,753 | 98.56 | 60256 | 841 |
| HC3DRt4 | 73,352 × 2 | 70,964 | 65,198 | 98.56 | 63207 | 957 |
| HC3DRt5 | 74,764 × 2 | 72,165 | 64,798 | 98.57 | 63028 | 953 |
| HC3DRt6 | 56,968 × 2 | 55,095 | 50,670 | 98.59 | 49102 | 831 |
| Z31DRt1 | 43,180× 2 | 40,923 | 36,721 | 98.1 | 35725 | 817 |
| Z31DRt2 | 70,889 × 2 | 68,610 | 60,545 | 98.65 | 58730 | 950 |
| Z31DRt3 | 49,602 × 2 | 47,091 | 41,260 | 98.25 | 40137 | 892 |
| Z31DRt4 | 58,382 × 2 | 56,418 | 50,964 | 98.59 | 49503 | 939 |
| Z31DRt5 | 48,928 × 2 | 46,541 | 42,658 | 98.21 | 41544 | 862 |
| Z31DRt6 | 65,053 × 2 | 62,981 | 56,176 | 98.66 | 54322 | 963 |

1. Clean Tags were obtained after connected tags were filtered to eliminate low quality and short sequence.
2. Effective Tags were obtained after clean tags were filtered to remove chimeras.
3. Six samples collected from six sampling points within three replicates/plots of surrounding soils of the transgenic soybean line Z31 or its recipient cultivar HC3 at seed-filling stage are named as Z31DSO1 ~ 6 or HC3DSO1 ~ 6, respectively.
4. Six samples collected from six sampling points within three replicates/plots of rhizosphere soils of the transgenic soybean line Z31 or its recipient cultivar HC3 at seed-filling stage are named as Z31DRh1 ~ 6 or HC3DRh1 ~ 6, respectively.
5. Six samples collected from six sampling points within three replicates/plots of root endosphere of the transgenic soybean line Z31 or its recipient cultivar HC3 at seed-filling stage are named as Z31DRt1 ~ 6 or HC3DRt1 ~ 6, respectively.
6. The “r” in the name of Z31DRh4r and Z31DRh6r indicated that PCR amplification of 16S rDNA and Illumina Miseq sequencing has been done again because the original clean data of Illumina Miseq sequencing from Z31DRh4 and Z31DRh6 metagenomic DNA were not enough.
