# Supplementary material for: Effects of an EPSPS-transgenic soybean line ZUTS31 on root-associated bacterial communities during field growth
Source: PLoS One. 2018 Feb 6;13(2):e0192008. doi: 10.1371/journal.pone.0192008 (PMC5800644; doi:10.1371/journal.pone.0192008)
Supplement: S7 Table — (DOCX) [file pone.0192008.s020.docx]

**S7 Table. Analysis codes of 100 samples’ clean data.**

| Analysis code | Data form | library ID | File name |
| --- | --- | --- | --- |
| 83707f20b5c621007f379cd601102690 | Clean_Data | HC3ASO1 | HC3ASO1_1.fq.gz |
| 7838e90de33bcb86e1f524d41515a50a | Clean_Data | HC3ASO1 | HC3ASO1_2.fq.gz |
| 07db7c8503f0baef1d17564d9a0e0243 | Clean_Data | HC3ASO3 | HC3ASO3_1.fq.gz |
| 7802a4d7ca64d81e35467a55221142de | Clean_Data | HC3ASO3 | HC3ASO3_2.fq.gz |
| a6909cc37dd7809c7f319055720e8483 | Clean_Data | HC3ASO5 | HC3ASO5_1.fq.gz |
| 4fae2a7b7178a44e95eb72b2999d2c9e | Clean_Data | HC3ASO5 | HC3ASO5_2.fq.gz |
| 1f02bbb08e9e3f2ef05b74453864e8f1 | Clean_Data | HC3ASO6 | HC3ASO6_1.fq.gz |
| 15bb9f08d77a8a50a82c9ed96df7aac7 | Clean_Data | HC3ASO6 | HC3ASO6_2.fq.gz |
| b1f7e39ad91d16ccfd00cb6490b74459 | Clean_Data | Z31ASO1 | Z31ASO1_1.fq.gz |
| 38b3f0224a569c20a436e3336c77f2d6 | Clean_Data | Z31ASO1 | Z31ASO1_2.fq.gz |
| 8fa3cc32d810ebf9b3ff267364335d46 | Clean_Data | Z31ASO3 | Z31ASO3_1.fq.gz |
| e96b17e173ae819fda1d5dba9f4ca9af | Clean_Data | Z31ASO3 | Z31ASO3_2.fq.gz |
| 6cc7e79e79724def26a253c8cbcded98 | Clean_Data | Z31ASO5 | Z31ASO5_1.fq.gz |
| 003000bbd082d8c9c4f839261a1464f0 | Clean_Data | Z31ASO5 | Z31ASO5_2.fq.gz |
| 2364abf08a4572ddb4ce10c3fb7924e5 | Clean_Data | Z31ASO6 | Z31ASO6_1.fq.gz |
| 5ee3fbc572fbd4a4d94065424c2c4351 | Clean_Data | Z31ASO6 | Z31ASO6_2.fq.gz |
| f2c114eeb098cb30cef864ba9c865208 | Clean_Data | HC3BSO1 | HC3BSO1_1.fq.gz |
| 1b223ee865544f3c33e325f36ffc586f | Clean_Data | HC3BSO1 | HC3BSO1_2.fq.gz |
| 3c02d47f78a7b5568c00cf5b77588351 | Clean_Data | HC3BSO2 | HC3BSO2_1.fq.gz |
| aeba01b7421caf451fa7246db04c650f | Clean_Data | HC3BSO2 | HC3BSO2_2.fq.gz |
| dabe65ca49b2a609e7fb75d6163f0912 | Clean_Data | HC3BSO3 | HC3BSO3_1.fq.gz |
| f4023902705026b92ab2b2459e274547 | Clean_Data | HC3BSO3 | HC3BSO3_2.fq.gz |
| 01891a6a435058412a9d34e7756f43bd | Clean_Data | HC3BSO4 | HC3BSO4_1.fq.gz |
| e968a26ccd488aed5f2956276cc7f08f | Clean_Data | HC3BSO4 | HC3BSO4_2.fq.gz |
| cf35bec37d257354bb51ada88e5f02dc | Clean_Data | HC3BSO5 | HC3BSO5_1.fq.gz |
| 8d36c6792c533bb2cf06c3bf36e452e8 | Clean_Data | HC3BSO5 | HC3BSO5_2.fq.gz |
| a082eec67178e8cfcbd9e9833518e373 | Clean_Data | HC3BSO6 | HC3BSO6_1.fq.gz |
| 8f40a54c8057ba58e5be1238fb7fb991 | Clean_Data | HC3BSO6 | HC3BSO6_2.fq.gz |
| a1bea56af3f01c1976dc6feef3ded33b | Clean_Data | Z31BSO1 | Z31BSO1_1.fq.gz |
| fe3edccbbf3d477d35d54dc003fd3ca5 | Clean_Data | Z31BSO1 | Z31BSO1_2.fq.gz |
| 9e1aafb0191c16f3f573f63cc8197aa6 | Clean_Data | Z31BSO2 | Z31BSO2_1.fq.gz |
| 56860a78a4b222c0b2db0d92a8817d8d | Clean_Data | Z31BSO2 | Z31BSO2_2.fq.gz |
| 2c26558bb8c4e3f76968b3409ea7e101 | Clean_Data | Z31BSO3 | Z31BSO3_1.fq.gz |
| 9aecc7a70298f93f3225210bd0145613 | Clean_Data | Z31BSO3 | Z31BSO3_2.fq.gz |
| e205f04db49499283e0b058a9175efae | Clean_Data | Z31BSO4 | Z31BSO4_1.fq.gz |
| 9d9037007b577895715e1f9315d791b0 | Clean_Data | Z31BSO4 | Z31BSO4_2.fq.gz |
| 5e4be29b9fafeac1d628495e22579a46 | Clean_Data | Z31BSO5 | Z31BSO5_1.fq.gz |
| 74c8a64df4a6daeddead0aaab9fdf684 | Clean_Data | Z31BSO5 | Z31BSO5_2.fq.gz |
| 27a678f2ac80f607e774cb001fec977b | Clean_Data | Z31BSO6 | Z31BSO6_1.fq.gz |
| 9da2e142c4cd6053bc90931665c19d04 | Clean_Data | Z31BSO6 | Z31BSO6_2.fq.gz |
| 46651976e87d0dafe9d91a4fe2851cc3 | Clean_Data | HC3BRh1 | HC3BRh1_1.fq.gz |
| 1c1d456bc115d7b482907b74acdc0311 | Clean_Data | HC3BRh1 | HC3BRh1_2.fq.gz |
| 1dd8e9760eb657787cb048d66dee157d | Clean_Data | HC3BRh2 | HC3BRh2_1.fq.gz |
| e6f5ada673ecd569ad5fb6a9c994f352 | Clean_Data | HC3BRh2 | HC3BRh2_2.fq.gz |
| 81832b55d3d84edc5150c8b02491419b | Clean_Data | HC3BRh3 | HC3BRh3_1.fq.gz |
| fb3e08cd483feb410d34e8e869b65b71 | Clean_Data | HC3BRh3 | HC3BRh3_2.fq.gz |
| 66cd144ced324a101b8b8a98e05cc77a | Clean_Data | HC3BRh4 | HC3BRh4_1.fq.gz |
| 90c3fcdaeadda8812cb833725119197c | Clean_Data | HC3BRh4 | HC3BRh4_2.fq.gz |
| 5844f8602fdfab4c58aa8bec640c692b | Clean_Data | HC3BRh5 | HC3BRh5_1.fq.gz |
| fcf5bb4d2a4026ea41fb7a971483df1a | Clean_Data | HC3BRh5 | HC3BRh5_2.fq.gz |
| 3839b28f07c51c162d80c03eb2ddaeb2 | Clean_Data | HC3BRh6 | HC3BRh6_1.fq.gz |
| 9b9276f5c2d8c5da854fee8ee1c58563 | Clean_Data | HC3BRh6 | HC3BRh6_2.fq.gz |
| fad3639c189390cb378d074cc963612c | Clean_Data | Z31BRh1 | Z31BRh1_1.fq.gz |
| 25ee6ffadd25788f9375c0e36cdd0ac4 | Clean_Data | Z31BRh1 | Z31BRh1_2.fq.gz |
| 96ff4e963494bde5d2c9d8e35ba62973 | Clean_Data | Z31BRh2 | Z31BRh2_1.fq.gz |
| 28ff8a7e2d1a2286ecbbc8cc15825e1e | Clean_Data | Z31BRh2 | Z31BRh2_2.fq.gz |
| 7cf053bd8dc3a49b948d59046220ec97 | Clean_Data | Z31BRh3 | Z31BRh3_1.fq.gz |
| 8a64eeb54215958ea672ed885eb0b6d2 | Clean_Data | Z31BRh3 | Z31BRh3_2.fq.gz |
| c126514c8241e98a9545a4f2fa5fbb25 | Clean_Data | Z31BRh4 | Z31BRh4_1.fq.gz |
| f67ad26dd61076cd4ea0fe976bcd033a | Clean_Data | Z31BRh4 | Z31BRh4_2.fq.gz |
| e529eba7ecd942793e358abb88c3a284 | Clean_Data | Z31BRh5 | Z31BRh5_1.fq.gz |
| c938a1cf6d8db682befeb36e32ba1e87 | Clean_Data | Z31BRh5 | Z31BRh5_2.fq.gz |
| a6008c5d155517f83e8d5d7343716ce9 | Clean_Data | Z31BRh6 | Z31BRh6_1.fq.gz |
| 852d85df65f28b799de6bab68ab2444c | Clean_Data | Z31BRh6 | Z31BRh6_2.fq.gz |
| 1d41c6a5bfc2d0ad0bcfa3c5a8815021 | Clean_Data | HC3CSO1 | HC3CSO1_1.fq.gz |
| e1a67df96ed06d72d65eff3d257de171 | Clean_Data | HC3CSO1 | HC3CSO1_2.fq.gz |
| 0c010370f6cf02ad949401fc1f5600fe | Clean_Data | HC3CSO2 | HC3CSO2_1.fq.gz |
| b147c84bc3586814dfdc577b3907b3b7 | Clean_Data | HC3CSO2 | HC3CSO2_2.fq.gz |
| 42129084e7b0a7fb247f04ea69691565 | Clean_Data | HC3CSO3 | HC3CSO3_1.fq.gz |
| 22bb0d091fafe84880832d65a8db8352 | Clean_Data | HC3CSO3 | HC3CSO3_2.fq.gz |
| 1d6e89045a4d0887c31017fbd19035ba | Clean_Data | HC3CSO4 | HC3CSO4_1.fq.gz |
| 5ecfa998d20db4790818347005e6c1fc | Clean_Data | HC3CSO4 | HC3CSO4_2.fq.gz |
| afd8303386032649cc636ae709918ea3 | Clean_Data | HC3CSO5 | HC3CSO5_1.fq.gz |
| 31ab70dda2965aac9ed82bd25fde2b61 | Clean_Data | HC3CSO5 | HC3CSO5_2.fq.gz |
| 6ae333fde9ddd950bef74051cda11aab | Clean_Data | HC3CSO6 | HC3CSO6_1.fq.gz |
| c15c752c4930a9e4770fbc1f1cfa3cdc | Clean_Data | HC3CSO6 | HC3CSO6_2.fq.gz |
| cf9ba2fcc38d6d892611b6598e53c784 | Clean_Data | Z31CSO1 | Z31CSO1_1.fq.gz |
| df9002761e6808fe8d56fe6314eff5fa | Clean_Data | Z31CSO1 | Z31CSO1_2.fq.gz |
| adac372bc664e00bbee528f650311749 | Clean_Data | Z31CSO2 | Z31CSO2_1.fq.gz |
| 9f655790a552dd5e9337780327eb9f3e | Clean_Data | Z31CSO2 | Z31CSO2_2.fq.gz |
| f033df864f3d0bbaf29765f61ecad6b2 | Clean_Data | Z31CSO3 | Z31CSO3_1.fq.gz |
| 2d212929b484ebe83fe400dd97a39743 | Clean_Data | Z31CSO3 | Z31CSO3_2.fq.gz |
| 6049d740f7bd5c664ad213d4ac9ffaa4 | Clean_Data | Z31CSO4 | Z31CSO4_1.fq.gz |
| 02eb4719b06003bc8622b341aaabdc71 | Clean_Data | Z31CSO4 | Z31CSO4_2.fq.gz |
| af4eeaaa34abd0c045d82e6c8bc506eb | Clean_Data | Z31CSO5 | Z31CSO5_1.fq.gz |
| af788234423d2457a643ce65b26d2535 | Clean_Data | Z31CSO5 | Z31CSO5_2.fq.gz |
| 7784a4210d062519df05578bfbbbf942 | Clean_Data | Z31CSO6 | Z31CSO6_1.fq.gz |
| 8b7519c7400a03cb94be9e2191e80d77 | Clean_Data | Z31CSO6 | Z31CSO6_2.fq.gz |
| 2edae7931026e662413032251a58b642 | Clean_Data | HC3CRh1 | HC3CRh1_1.fq.gz |
| 3e5b9ba12ace6cbc1c2527add3dc956a | Clean_Data | HC3CRh1 | HC3CRh1_2.fq.gz |
| 8e81e191d8c93459d9e46358551ddca3 | Clean_Data | HC3CRh2 | HC3CRh2_1.fq.gz |
| 774abe3c76028d8ee8380217b77a13bf | Clean_Data | HC3CRh2 | HC3CRh2_2.fq.gz |
| d9fc95d884f422ffb7fced83de78a870 | Clean_Data | HC3CRh3 | HC3CRh3_1.fq.gz |
| e26842012677d1a4ffd80ed09361edcd | Clean_Data | HC3CRh3 | HC3CRh3_2.fq.gz |
| 40f87823bd9eae6ce86ce1936a8c2972 | Clean_Data | HC3CRh4 | HC3CRh4_1.fq.gz |
| db3c3cd124bd2b230542d26afcf41b4d | Clean_Data | HC3CRh4 | HC3CRh4_2.fq.gz |
| 326e55ebe838f4c240b10284ccc52719 | Clean_Data | HC3CRh5 | HC3CRh5_1.fq.gz |
| 8623707ee3bd914001e7898ae1f117ad | Clean_Data | HC3CRh5 | HC3CRh5_2.fq.gz |
| 71ca2868ec34c2823b1ede45e69015c1 | Clean_Data | HC3CRh6 | HC3CRh6_1.fq.gz |
| 20e52ad154e81b96955202b6d42db51d | Clean_Data | HC3CRh6 | HC3CRh6_2.fq.gz |
| bf7d9245337e506487d39abdcb27da14 | Clean_Data | Z31CRh1 | Z31CRh1_1.fq.gz |
| a9bbf44ec2a2431f211e210d1ae44f22 | Clean_Data | Z31CRh1 | Z31CRh1_2.fq.gz |
| fccc903222ba3595871a5c88234372c4 | Clean_Data | Z31CRh2 | Z31CRh2_1.fq.gz |
| 21307926dee8d062609d94bace42a84c | Clean_Data | Z31CRh2 | Z31CRh2_2.fq.gz |
| e92bb2c9dd199b8cc02c2818c4211e14 | Clean_Data | Z31CRh3 | Z31CRh3_1.fq.gz |
| 8d63a59f7319b23c91223fcaf7ba3b02 | Clean_Data | Z31CRh3 | Z31CRh3_2.fq.gz |
| a98c03e9d98177b108263876929dcf37 | Clean_Data | Z31CRh4 | Z31CRh4_1.fq.gz |
| d0cf1ee76e2437d144e35a24b2a0dc37 | Clean_Data | Z31CRh4 | Z31CRh4_2.fq.gz |
| f3c90053b3beb9c5f1938b77a2ae8965 | Clean_Data | Z31CRh5 | Z31CRh5_1.fq.gz |
| a409e0ebd1f5a45841928e31931e4a99 | Clean_Data | Z31CRh5 | Z31CRh5_2.fq.gz |
| d8bb7a3d1c2bbc34e8927f045960af0c | Clean_Data | Z31CRh6 | Z31CRh6_1.fq.gz |
| 989296477123745e8a1545d63b30fd82 | Clean_Data | Z31CRh6 | Z31CRh6_2.fq.gz |
| 1455fa0be025b396889b5c4265802fb3 | Clean_Data | HC3CRt2 | HC3CRt2_1.fq.gz |
| 46b4d1f952084f69ed1009a44b59be6a | Clean_Data | HC3CRt2 | HC3CRt2_2.fq.gz |
| de3d802a0a9e4c620e8e58edabaf313d | Clean_Data | HC3CRt3 | HC3CRt3_1.fq.gz |
| fa9b84b4d0687ff3c3b4fe31eb4eca18 | Clean_Data | HC3CRt3 | HC3CRt3_2.fq.gz |
| 3608037d9a695be7a9328c62ae646d8c | Clean_Data | HC3CRt5 | HC3CRt5_1.fq.gz |
| 0e80589dd915d8cc296f047f09e9e844 | Clean_Data | HC3CRt5 | HC3CRt5_2.fq.gz |
| b6f556655ad3c0f14623d8f660c0dd05 | Clean_Data | HC3CRt6 | HC3CRt6_1.fq.gz |
| 9a9eb4360d5bedf18ea65a665b3b2fb9 | Clean_Data | HC3CRt6 | HC3CRt6_2.fq.gz |
| 289c1202008365a050e5259faff3949a | Clean_Data | Z31CRt1 | Z31CRt1_1.fq.gz |
| d4f7b75d232bfb5cd682ef0ed5ac18a4 | Clean_Data | Z31CRt1 | Z31CRt1_2.fq.gz |
| 325c85c5484aaf55851fbebf524d6ece | Clean_Data | Z31CRt4 | Z31CRt4_1.fq.gz |
| 4c882f559e6a3bdaa9515cd73bf6b1a5 | Clean_Data | Z31CRt4 | Z31CRt4_2.fq.gz |
| d7c3ed31ae8037941a64201c8cc02c5c | Clean_Data | Z31CRt5 | Z31CRt5_1.fq.gz |
| 05560650c7f14feeb3ff743a26e76e01 | Clean_Data | Z31CRt5 | Z31CRt5_2.fq.gz |
| b5bb0b4808179438784fc7a3ee3724ad | Clean_Data | Z31CRt6 | Z31CRt6_1.fq.gz |
| 8714fc24b359b2936f5ac68d587ea06b | Clean_Data | Z31CRt6 | Z31CRt6_2.fq.gz |
| e343b3a8a88f5afa09a2c876cbd4bc1c | Clean_Data | HC3DSO1 | HC3DSO1_1.fq.gz |
| 9097cdaecf4a07cdc8151f9497d28a9b | Clean_Data | HC3DSO1 | HC3DSO1_2.fq.gz |
| 8e681c3465b30e99dfa51f8761ca6880 | Clean_Data | HC3DSO2 | HC3DSO2_1.fq.gz |
| d1be6310396b5354d229959fb1aed318 | Clean_Data | HC3DSO2 | HC3DSO2_2.fq.gz |
| a29502dec8d2d54c2428bf833aa8b037 | Clean_Data | HC3DSO3 | HC3DSO3_1.fq.gz |
| c773f9da75bd98bf62941b839d5388e4 | Clean_Data | HC3DSO3 | HC3DSO3_2.fq.gz |
| d361f18515356e1a6d7a33f513684aa5 | Clean_Data | HC3DSO4 | HC3DSO4_1.fq.gz |
| 8890a9244919cefe30a94c93f7ac0566 | Clean_Data | HC3DSO4 | HC3DSO4_2.fq.gz |
| 98f430847ef2e7ca8d2f288ea3ee0d60 | Clean_Data | HC3DSO5 | HC3DSO5_1.fq.gz |
| b063d8128c6ce9e5add7eaea5ac7912e | Clean_Data | HC3DSO5 | HC3DSO5_2.fq.gz |
| d0e822aa9137ff7c35d9b448aca3b2d9 | Clean_Data | HC3DSO6 | HC3DSO6_1.fq.gz |
| 1893289a3e16811f2b2624a814e95bb2 | Clean_Data | HC3DSO6 | HC3DSO6_2.fq.gz |
| 19d55c30da06bb9308d0555495353dc7 | Clean_Data | Z31DSO1r | Z31DSO1r_1.fq.gz |
| 742a612215a9bb7382f8505b5ba5685c | Clean_Data | Z31DSO1r | Z31DSO1r_2.fq.gz |
| 8e4129cf9ad52c97f5dcd78f2d78837b | Clean_Data | Z31DSO2 | Z31DSO2_1.fq.gz |
| 188e8c98f8756641d7ccded0a0e9f15b | Clean_Data | Z31DSO2 | Z31DSO2_2.fq.gz |
| cad85bcba1a43137ccf259139d26b1fd | Clean_Data | Z31DSO3 | Z31DSO3_1.fq.gz |
| d758faa149b4e25e5771975c924c0d20 | Clean_Data | Z31DSO3 | Z31DSO3_2.fq.gz |
| 979883fe7a3154bff8493db5c23df4b4 | Clean_Data | Z31DSO | Z31DSO4_1.fq.gz |
| a404de6767fa7674f9ea7e2a1a8a01ad | Clean_Data | Z31DSO | Z31DSO4_2.fq.gz |
| 763ed7552b48c5bb794fd7f35294121a | Clean_Data | Z31DSO5 | Z31DSO5_1.fq.gz |
| b9e54b99acaa4a68db366ab7d2d27145 | Clean_Data | Z31DSO5 | Z31DSO5_2.fq.gz |
| 6271dc27b1945c490dead7136ac72699 | Clean_Data | Z31DSO6 | Z31DSO6_1.fq.gz |
| e7a7249d9546c673e763050987e045d9 | Clean_Data | Z31DSO6 | Z31DSO6_2.fq.gz |
| b49d31802a48acf47aa3bc425afc6318 | Clean_Data | HC3DRh1 | HC3DRh1_1.fq.gz |
| cd2b1b0829ce05fbc63abbd4b5ffaa7a | Clean_Data | HC3DRh1 | HC3DRh1_2.fq.gz |
| 36c98c77d9c8f4178133c05b85fa866f | Clean_Data | HC3DRh2 | HC3DRh2_1.fq.gz |
| 93f4f67366a9ec5d155129967df279e2 | Clean_Data | HC3DRh2 | HC3DRh2_2.fq.gz |
| 13ba8e4efbe12cac4d0b409e9c490c5d | Clean_Data | HC3DRh3 | HC3DRh3_1.fq.gz |
| f8e76e378d77b553405fc36dce9a0ecd | Clean_Data | HC3DRh3 | HC3DRh3_2.fq.gz |
| f51224999e41c5107b5228db6e6dbb34 | Clean_Data | HC3DRh4 | HC3DRh4_1.fq.gz |
| c1485c12d164990f1a003f757cd55433 | Clean_Data | HC3DRh4 | HC3DRh4_2.fq.gz |
| 7c00c5b5747ae822625bbd8b482f4da5 | Clean_Data | HC3DRh5 | HC3DRh5_1.fq.gz |
| 54fc1d1ce9b83f08b00a72ba9e5c605d | Clean_Data | HC3DRh5 | HC3DRh5_2.fq.gz |
| 6a96562edf7244dc85821945815cdb36 | Clean_Data | HC3DRh6 | HC3DRh6_1.fq.gz |
| 4634e8cc3f8679d84d36e15de1be02ba | Clean_Data | HC3DRh6 | HC3DRh6_2.fq.gz |
| 59963ff0e75dc580a23eaba704b4077b | Clean_Data | Z31DRh1 | Z31DRh1_1.fq.gz |
| 39df9fca34176d1b4fe1ab49b57e98c3 | Clean_Data | Z31DRh1 | Z31DRh1_2.fq.gz |
| 6caa8577fab006a7afc85c52773e84dd | Clean_Data | Z31DRh2 | Z31DRh2_1.fq.gz |
| 46395e8379d1124622b65c6ca48866a6 | Clean_Data | Z31DRh2 | Z31DRh2_2.fq.gz |
| 5dcda0de39f3a70aaacbfa6a9a53af28 | Clean_Data | Z31DRh3 | Z31DRh3_1.fq.gz |
| 4f034eb86b868fd629bb43dcbd72b3bc | Clean_Data | Z31DRh3 | Z31DRh3_2.fq.gz |
| 7d1883c76db729b76143fa496b7b13e5 | Clean_Data | Z31DRh4r | Z31DRh4r_1.fq.gz |
| 16e8e81fd476086f47698cea4449bf65 | Clean_Data | Z31DRh4r | Z31DRh4r_2.fq.gz |
| f5b87861ad02031524fcf1a9600aff1e | Clean_Data | Z31DRh5 | Z31DRh5_1.fq.gz |
| 26049a169daacfe575e1f8331b3005ec | Clean_Data | Z31DRh5 | Z31DRh5_2.fq.gz |
| 8d4bfd304f6dec5bcd6199e65a4946d9 | Clean_Data | Z31DRh6r | Z31DRh6r_1.fq.gz |
| b02e36c292f258aa6dd5f952cd6b83c4 | Clean_Data | Z31DRh6r | Z31DRh6r_2.fq.gz |
| 2514826bafda9d827f4ccae486a2386f | Clean_Data | HC3DRt1 | HC3DRt1_1.fq.gz |
| c8522cfff91321211f8259a65d1ace85 | Clean_Data | HC3DRt1 | HC3DRt1_2.fq.gz |
| 82a87967b6a0634aea8c5f531ff69d56 | Clean_Data | HC3DRt2 | HC3DRt2_1.fq.gz |
| 39a8c87147763f90fb1104e02465fc90 | Clean_Data | HC3DRt2 | HC3DRt2_2.fq.gz |
| 887d4a98996d6d0c1e61c9a1ec957377 | Clean_Data | HC3DRt3 | HC3DRt3_1.fq.gz |
| 29b4cf4c3277a860366b20d1d7283122 | Clean_Data | HC3DRt3 | HC3DRt3_2.fq.gz |
| fbd9a89c87ff4fbaf57706952bf8b648 | Clean_Data | HC3DRt4 | HC3DRt4_1.fq.gz |
| 1ebbe472c99c25d659caf177451a1598 | Clean_Data | HC3DRt4 | HC3DRt4_2.fq.gz |
| 63019ab36b79d811eaa7d9f26c8c598c | Clean_Data | HC3DRt5 | HC3DRt5_1.fq.gz |
| e157033fff09ffe30c9ae1d856f5eccb | Clean_Data | HC3DRt5 | HC3DRt5_2.fq.gz |
| 1875a96fea29efdcae961c273346c226 | Clean_Data | HC3DRt6 | HC3DRt6_2.fq.gz |
| b9c396fb58d0142d728d2c5b079f87ab | Clean_Data | HC3DRt6 | HC3DRt6_2.fq.gz |
| 2d097e668c87b0f2122dec9821464db0 | Clean_Data | Z31DRt1 | Z31DRt1_1.fq.gz |
| 9e9d6a173cf5ab7f76c21ef6397365ea | Clean_Data | Z31DRt1 | Z31DRt1_2.fq.gz |
| 3acd8b53266c927ee183824ec124b79e | Clean_Data | Z31DRt2 | Z31DRt2_1.fq.gz |
| 3fa78836d8f7d29eae0e57bc45c71d64 | Clean_Data | Z31DRt2 | Z31DRt2_2.fq.gz |
| 151605915a87f7f693cbd72ed1d17ee2 | Clean_Data | Z31DRt3 | Z31DRt3_1.fq.gz |
| 237c853afb3f37486f7f6c8d97a06197 | Clean_Data | Z31DRt3 | Z31DRt3_2.fq.gz |
| b67302bd211f15281fb594fd1c09c4b2 | Clean_Data | Z31DRt4 | Z31DRt4_1.fq.gz |
| 0824080f49052fd2eba1af7df07a8c7c | Clean_Data | Z31DRt4 | Z31DRt4_2.fq.gz |
| cdb185d24d27af002e50744361773a35 | Clean_Data | Z31DRt5 | Z31DRt5_1.fq.gz |
| 826809bd192f6da89f97d4b8884b3099 | Clean_Data | Z31DRt5 | Z31DRt5_2.fq.gz |
| e3bd24c44ca1ca05535373c172c51452 | Clean_Data | Z31DRt6 | Z31DRt6_1.fq.gz |
| 3a55a31c8e49372e2eefbb2d1c202444 | Clean_Data | Z31DRt6 | Z31DRt6_2.fq.gz |
