# Supplementary material for: Effects of an EPSPS-transgenic soybean line ZUTS31 on root-associated bacterial communities during field growth
Source: PLoS One. 2018 Feb 6;13(2):e0192008. doi: 10.1371/journal.pone.0192008 (PMC5800644; doi:10.1371/journal.pone.0192008)
Supplement: S8 Table — (DOC) [file pone.0192008.s021.doc]

**S8 Table. Comparison of the alpha diversity of rhizospheric soils bacterial communities between the *EPSPS*-transgenic soybean line Z31 and its recipient cultivar HC3 at the vegetative stage.**

| Alpha diversity index | Rhizosphere soil of the transgenic line Z31 at vegetative stage (Z31BRh) | | Rhizosphere soil of its recipient cultivar HC3 at vegetative stage (HC3BRh) | | *p*-value (Wilcoxon) | *p*-value (Tukey) |
| --- | --- | --- | --- | --- | --- | --- |
|  | Mean | SD | Mean | SD |  |  |
| Observed_ OTUs | 2855.83 | 139.36 | 2982.00 | 253.55 | 0.39178 | 0.79901 |
| Chao 1 | 3238.78 | 172.90 | 3404.13 | 338.31 | 0.48953 | 0.86738 |
| ACE | 3331.65 | 184.18 | 3487.47 | 302.45 | 0.53578 | 0.87551 |
| Shannon | 9.4170 | 0.2996 | 9.4308 | 0.3835 | 0.86862 | 0.10000 |
| Simpson | 0.99433 | 0.00327 | 0.99367 | 0.00350 | 0.47255 | 0.99560 |
| Good’s coverage | 0.98300 | 0.00167 | 0.98183 | 0.00204 | 0.33881 | 0.94652 |

SD, standard deviation; ACE, abundance coverage-based estimator.

The significance test methods were Wilcoxon rank-sum Test (Wilcoxon) and Tukey’s HSD test (Tukey).
