# Supplementary material for: Effects of an EPSPS-transgenic soybean line ZUTS31 on root-associated bacterial communities during field growth
Source: PLoS One. 2018 Feb 6;13(2):e0192008. doi: 10.1371/journal.pone.0192008 (PMC5800644; doi:10.1371/journal.pone.0192008)
Supplement: S9 Table — (DOC) [file pone.0192008.s022.doc]

**S9 Table. Comparison of the alpha diversity of rhizosphere soil and roots bacterial communities between the *EPSPS*-transgenic soybean line Z31 and its recipient cultivar HC3 at the flowering stage.**

| Alpha diversity index | Rhizosphere soil of the transgenic line Z31 at flowering stage (Z31CRh) | | Rhizosphere soil of its recipient cultivar HC3 at flowering stage (HC3CRh) | | *p*-value (Wilcoxon) | *p*-value (Tukey) | Roots of the transgenic line Z31 at flowering stage (Z31CRt) | | Roots of its recipient cultivar HC3 at flowering stage (HC3CRt) | | *p*-value (Wilcoxon) | *p*-value (Tukey) |
| --- | --- | --- | --- | --- | --- | --- | --- | --- | --- | --- | --- | --- |
|  | Mean | SD | Mean | SD |  |  | Mean | SD | Mean | SD |  |  |
| Observed_ OTUs | 2800.17 | 75.44 | 2724.17 | 161.29 | 0.32789 | 0.85367 | 310.25 | 39.31 | 299.75 | 35.80 | 0.89870 | 0.99999 |
| Chao 1 | 3176.39 | 93.42 | 3170.16 | 153.17 | 0.87605 | 1.00000 | 368.09 | 63.49 | 355.26 | 58.24 | 0.89865 | 0.99999 |
| ACE | 3316.39 | 82.45 | 3254.32 | 162.29 | 0.53609 | 0.98448 | 381.46 | 60.55 | 372.45 | 54.58 | 0.89865 | 1.00000 |
| Shannon | 9.3520 | 0.0852 | 9.1755 | 0.2364 | 0.18843 | 0.45710 | 2.1005 | 0.2448 | 2.0003 | 0.0991 | 0.87200 | 0.95377 |
| Simpson | 0.99517 | 0.00041 | 0.99350 | 0.00217 | 0.10277 | 0.99997 | 0.56600 | 0.04390 | 0.56975 | 0.01664 | 0.79139 | 0.99940 |
| Good’s coverage | 0.98233 | 0.00052 | 0.98233 | 0.00137 | 0.54815 | 1.0000 | 0.99775 | 0.0005 | 0.99750 | 0.00058 | 0.79751 | 0.99992 |

SD, standard deviation; ACE, abundance coverage-based estimator.

The significance test methods were Wilcoxon rank-sum Test (Wilcoxon) and Tukey’s HSD test (Tukey).
