# Supplementary material for: Effects of an EPSPS-transgenic soybean line ZUTS31 on root-associated bacterial communities during field growth
Source: PLoS One. 2018 Feb 6;13(2):e0192008. doi: 10.1371/journal.pone.0192008 (PMC5800644; doi:10.1371/journal.pone.0192008)
Supplement: S10 Table — (DOC) [file pone.0192008.s023.doc]

**S10 Table. Comparison of the alpha diversity of rhizospheric soils and roots bacterial communities between the *EPSPS*-transgenic soybean line Z31 and its recipient cultivar HC3 at the seed-filling stage.**

| Alpha diversity index | Rhizosphere soil of the transgenic line Z31 at seed-filling stage (Z31DRh) | | Rhizosphere soil of its recipient cultivar HC3 at seed-filling stage (HC3DRh) | | *p*-value (Wilcoxon) | *p*-value (Tukey) | Roots of the transgenic line Z31 at seed-filling stage (Z31DRt) | | Roots of its recipient cultivar HC3 at seed-filling stage (HC3DRt) | | *p*-value (Wilcoxon) | *p*-value (Tukey) |
| --- | --- | --- | --- | --- | --- | --- | --- | --- | --- | --- | --- | --- |
|  | Mean | SD | Mean | SD |  |  | Mean | SD | Mean | SD |  |  |
| Observed_OTUs | 2412.50 | 166.50 | 2511.83 | 118.87 | 0.21003 | 0.87422 | 703.67 | 32.28 | 668.50 | 79.90 | 0.28706 | 0.99867 |
| Chao 1 | 3096.52 | 310.24 | 3324.54 | 193.26 | 0.15052 | 0.76433 | 942.84 | 136.96 | 973.44 | 153.16 | 0.77718 | 0.99997 |
| ACE | 3148.68 | 261.37 | 3392.91 | 219.17 | 0.15667 | 0.73204 | 1013.98 | 131.88 | 1040.43 | 180.70 | 0.92595 | 0.99999 |
| Shannon | 9.1365 | 0.3049 | 9.2725 | 0.1405 | 0.40089 | 0.93753 | 4.1143 | 0.2189 | 3.7895 | 0.3667 | 0.37411 | 0.26578 |
| Simpson | 0.99383 | 0.00232 | 0.99533 | 0.00103 | 0.16990 | 1.00000 | 0.80383 | 0.03304 | 0.78100 | 0.05460 | 0.75681 | 0.65707 |
| Good’s coverage | 0.97400 | 0.00329 | 0.97083 | 0.00248 | 0.14721 | 0.70108 | 0.99083 | 0.00160 | 0.99017 | 0.00172 | 0.73922 | 0.99615 |

SD, standard deviation; ACE, abundance coverage-based estimator.

The significance test methods were Wilcoxon rank-sum Test (Wilcoxon) and Tukey’s HSD test (Tukey).
