# Supplementary material for: Effects of an EPSPS-transgenic soybean line ZUTS31 on root-associated bacterial communities during field growth
Source: PLoS One. 2018 Feb 6;13(2):e0192008. doi: 10.1371/journal.pone.0192008 (PMC5800644; doi:10.1371/journal.pone.0192008)
Supplement: S11 Table — (DOC) [file pone.0192008.s024.doc]

**S11 Table. Comparison of alpha diversity of bulk soils bacterial communities between the *EPSPS*-transgenic soybean line Z31 and its recipient cultivar HC3 before sowing soybean seeds.**

| Alpha diversity index | Bulk soil of the transgenic line Z31 (Z31ASO) | | Bulk soil of its recipient cultivar HC3 (HC3ASO) | | *p*-value (Wilcoxon) | *p*-value (Tukey) |
| --- | --- | --- | --- | --- | --- | --- |
|  | Mean | SD | Mean | SD |  |  |
| Observed_  OTUs | 2522.75 | 145.90 | 2624.00 | 125.07 | 0.42136 | 0.95890 |
| Chao 1 | 2866.40 | 197.40 | 3019.22 | 117.49 | 0.55306 | 0.95543 |
| ACE | 2933.05 | 187.66 | 3125.58 | 136.57 | 0.33511 | 0.87148 |
| Shannon | 9.1805 | 0.2509 | 9.2258 | 0.1819 | 0.98530 | 0.99989 |
| Simpson | 0.99475 | 0.00171 | 0.99525 | 0.00096 | 0.64581 | 0.99958 |
| Good’s coverage | 0.98500 | 0.00115 | 0.98325 | 0.00050 | 0.07243 | 0.88287 |

SD, standard deviation; ACE, abundance coverage-based estimator.

The significance test methods were Wilcoxon Rank-Sum Test (Wilcoxon) and Tukey HSD test (Tukey).
