# Supplementary material for: Effects of an EPSPS-transgenic soybean line ZUTS31 on root-associated bacterial communities during field growth
Source: PLoS One. 2018 Feb 6;13(2):e0192008. doi: 10.1371/journal.pone.0192008 (PMC5800644; doi:10.1371/journal.pone.0192008)
Supplement: S12 Table — (DOC) [file pone.0192008.s025.doc]

**S12 Table. Comparison of alpha diversity of surrounding soils bacterial communities between the *EPSPS*-transgenic soybean line Z31 and its recipient cultivar HC3 at vegetative stage.**

| Alpha diversity index | Surrounding soil of the transgenic line Z31 at vegetative stage (Z31BSO) | | Surrounding soil of its recipient cultivar HC3 at vegetative stage (HC3BSO) | | *p*-value (Wilcoxon) | *p*-value (Tukey) |
| --- | --- | --- | --- | --- | --- | --- |
|  | Mean | SD | Mean | SD |  |  |
| Observed_ OTUs | 2779.33 | 146.24 | 2766.33 | 164.72 | 0.89780 | 0.99999 |
| Chao 1 | 3287.94 | 348.53 | 3164.25 | 201.81 | 0.62775 | 0.95702 |
| ACE | 3358.54 | 348.45 | 3257.74 | 178.65 | 0.80911 | 0.97852 |
| Shannon | 9.4023 | 0.1863 | 9.3335 | 0.2417 | 0.76376 | 0.99774 |
| Simpson | 0.99567 | 0.00082 | 0.99533 | 0.00121 | 0.84503 | 0.99984 |
| Good’s coverage | 0.98183 | 0.00422 | 0.98283 | 0.00117 | 0.58402 | 0.97192 |

SD, standard deviation; ACE, abundance coverage-based estimator.

The significance test methods were Wilcoxon Rank-Sum Test (Wilcoxon) and Tukey HSD test (Tukey).
