# Supplementary material for: Effects of an EPSPS-transgenic soybean line ZUTS31 on root-associated bacterial communities during field growth
Source: PLoS One. 2018 Feb 6;13(2):e0192008. doi: 10.1371/journal.pone.0192008 (PMC5800644; doi:10.1371/journal.pone.0192008)
Supplement: S13 Table — (DOC) [file pone.0192008.s026.doc]

**S13 Table. Comparison of alpha diversity of surrounding soils bacterial communities between the *EPSPS*-transgenic soybean line Z31 and its recipient cultivar HC3 at the flowering stage.**

| Alpha diversity index | Surrounding soil of the transgenic line Z31 at flowering stage (Z31CSO) | | Surrounding soil its recipient cultivar HC3 at flowering stage (HC3CSO) | | *p*-value (Wilcoxon) | *p*-value (Tukey) |
| --- | --- | --- | --- | --- | --- | --- |
|  | Mean | SD | Mean | SD |  |  |
| Observed_ OTUs | 2589.67 | 122.67 | 2640.67 | 138.33 | 0.28052 | 0.96940 |
| Chao 1 | 2978.03 | 137.00 | 3104.78 | 315.75 | 0.28025 | 0.79752 |
| ACE | 3057.13 | 170.80 | 3171.09 | 265.27 | 0.30560 | 0.82444 |
| Shannon | 9.3053 | 0.1115 | 9.3078 | 0.1598 | 0.91269 | 1.00000 |
| Simpson | 0.99567 | 0.00052 | 0.99533 | 0.00082 | 0.39075 | 1.00000 |
| Good’s coverage | 0.98383 | 0.00133 | 0.98267 | 0.00301 | 0.44987 | 0.80184 |

SD, standard deviation; ACE, abundance coverage-based estimator.

The significance test methods were Wilcoxon Rank-Sum Test (Wilcoxon) and Tukey HSD test (Tukey).
