# Supplementary material for: Effects of an EPSPS-transgenic soybean line ZUTS31 on root-associated bacterial communities during field growth
Source: PLoS One. 2018 Feb 6;13(2):e0192008. doi: 10.1371/journal.pone.0192008 (PMC5800644; doi:10.1371/journal.pone.0192008)
Supplement: S14 Table — (DOC) [file pone.0192008.s027.doc]

**S14 Table. Comparison of alpha diversity of surrounding soils bacterial communities between the *EPSPS*-transgenic soybean line Z31 and its recipient cultivar HC3 at the seed-filling stage.**

| Alpha diversity index | Surrounding soil of the transgenic line Z31 at seed-filling stage (Z31DSO) | | Surrounding soil of its recipient cultivar HC3 at seed-filling stage  (HC3DSO) | | *p*-value (Wilcoxon) | *p*-value (Tukey) |
| --- | --- | --- | --- | --- | --- | --- |
|  | Mean | SD | Mean | SD |  |  |
| Observed_ OTUs | 2563.17 | 158.17 | 2677.67 | 265.28 | 0.49566 | 0.79477 |
| Chao 1 | 3185.44 | 439.34 | 3455.20 | 396.66 | 0.10617 | 0.61874 |
| ACE | 3261.58 | 453.94 | 3534.80 | 427.39 | 0.21550 | 0. 63241 |
| Shannon | 9.4443 | 0.1036 | 9.4382 | 0.2920 | 0.45798 | 1.00000 |
| Simpson | 0.99583 | 0.00117 | 0.99600 | 0.00110 | 0.71797 | 1.00000 |
| Good’s coverage | 0.97433 | 0.00635 | 0.97033 | 0.00489 | 0.09428 | 0.46681 |

SD, standard deviation; ACE, abundance coverage-based estimator.

The significance test methods were Wilcoxon Rank-Sum Test (Wilcoxon) and Tukey HSD test (Tukey).
