# Supplementary material for: Effects of an EPSPS-transgenic soybean line ZUTS31 on root-associated bacterial communities during field growth
Source: PLoS One. 2018 Feb 6;13(2):e0192008. doi: 10.1371/journal.pone.0192008 (PMC5800644; doi:10.1371/journal.pone.0192008)
Supplement: S15 Table — (DOC) [file pone.0192008.s028.doc]

**S15 Table. Adonis analysis of bulk soil, surrounding soil, rhizosphere soil bacterial communities between Z31 and HC3 based on Bray-Curtis distance at the vegetative stage**.

| **Group vs. Group** | **Df** | **Sums Of Sqs** | **Mean Sqs** | **F. Model** | **R^2^** | **Pr(>F)** |
| --- | --- | --- | --- | --- | --- | --- |
| **Z31ASO vs. HC3ASO** | 1(6) | 0.040869(0.245579) | 0.040869(0.040930) | 0.99852 | 0.14268(0.85732) | 0.402 |
| **Z31BSO vs. HC3BSO** | 1(10) | 0.07865(0.61080) | 0.07865(0.06108) | 1.2876 | 0.11408(0.88592) | 0.154 |
| **Z31BRh vs. HC3BRh** | 1(10) | 0.09486(0.57869) | 0.094858(0.057869) | 1.6392 | 0.14083(0.85917) | **0.005** |
| Z31ASO **vs.** Z31BRh | 1(8) | 0.27808(0.42009) | 0.278084(0.052512) | 5.2957 | 0.3983(0.6017) | 0.001 |
| Z31ASO **vs.** Z31BSO | 1(8) | 0.13304(0.53060) | 0.133038(0.066325) | 2.0058 | 0.20047(0.79953) | 0.057 |
| Z31ASO **vs.** HC3BRh | 1(8) | 0.32167(0.41780) | 0.32167(0.05222) | 6.1594 | 0.435(0.565) | 0.001 |
| Z31ASO **vs.** HC3BSO | 1(8) | 0.19349(0.33939) | 0.193493(0.042424) | 4.561 | 0.36311(0.63689) | 0.002 |
| HC3ASO **vs.** Z31BRh | 1(8) | 0.27999(0.40648) | 0.27999(0.05081) | 5.5106 | 0.40787(0.59213) | 0.009 |
| HC3ASO **vs.** Z31BSO | 1(8) | 0.11538(0.51699) | 0.115380(0.064623) | 1.7854 | 0.18246(0.81754) | 0.054 |
| HC3ASO **vs.** HC3BRh | 1(8) | 0.32388(0.40418) | 0.32388(0.05052) | 6.4106 | 0.44485(0.55515) | 0.006 |
| HC3ASO **vs.** HC3BSO | 1(8) | 0.15456(0.32577) | 0.154556(0.040722) | 3.7954 | 0.32177(0.67823) | 0.001 |
| Z31BRh **vs.** Z31BSO | 1(10) | 0.22931(0.69150) | 0.22931(0.06915) | 3.3161 | 0.24903(0.75097) | 0.001 |
| Z31BRh **vs.** HC3BSO | 1(10) | 0.29640(0.50029) | 0.296402(0.050029) | 5.9247 | 0.37204(0.62796) | 0.001 |
| Z31BSO **vs.** HC3BRh | 1(10) | 0.28867(0.68920) | 0.28867(0.06892) | 4.1884 | 0.2952(0.7048) | 0.001 |
| HC3BRh **vs.** HC3BSO | 1(10) | 0.32892(0.49799) | 0.32892(0.04980) | 6.605 | 0.39777(0.60223) | 0.004 |

An ADONIS difference, which was calculated based on Bray–Curtis distance, is a non-parametric method to measure statistical significance of sample grouping. **, Pr < 0.01. Residuals were presented in parentheses.

ASO, bulk soil was collected from field before sowing soybean seeds; BSO, surrounding soil at the vegetative stage; BRh, rhizosphere soil at the vegetative stage.
