# Supplementary material for: Effects of an EPSPS-transgenic soybean line ZUTS31 on root-associated bacterial communities during field growth
Source: PLoS One. 2018 Feb 6;13(2):e0192008. doi: 10.1371/journal.pone.0192008 (PMC5800644; doi:10.1371/journal.pone.0192008)
Supplement: S16 Table — (DOC) [file pone.0192008.s029.doc]

**S16 Table. Multiple response permutation procedure (MRPP) analysis of bulk soil , surrounding soil, rhizosphere soil of Z31 and HC3 at vegetative stage based on Bray-Curtis metric distances.**

| Group vs. Group | A | Observed-delta | Expected-delta | *P*-value |
| --- | --- | --- | --- | --- |
| **Z31ASO vs. HC3ASO** | -0.00188 | 0.2840 | 0.2834 | 0.429 |
| **Z31BSO vs. HC3BSO** | 0.01647 | 0.3418 | 0.3475 | 0.122 |
| **Z31BRh vs. HC3BRh** | 0.02759 | 0.3367 | 0.3467 | **0.006** |
| HC3BSO vs. Z31ASO | 0.1493 | 0.2883 | 0.3389 | **0.005** |
| HC3ASO vs. HC3BSO | 0.1233 | 0.2829 | 0.3227 | **0.003** |
| HC3BRh vs. HC3BSO | 0.1791 | 0.3115 | 0.3794 | **0.003** |
| HC3BRh vs. Z31ASO | 0.1986 | 0.318 | 0.3968 | **0.004** |
| HC3ASO vs. HC3BRh | 0.2057 | 0.3127 | 0.3936 | **0.005** |
| HC3BSO vs. Z31BRh | 0.1633 | 0.312 | 0.3729 | **0.003** |
| Z31ASO vs. Z31BRh | 0.1758 | 0.3186 | 0.3865 | **0.004** |
| HC3ASO vs. Z31BRh | 0.1822 | 0.3133 | 0.3831 | **0.006** |
| Z31ASO vs. Z31BSO | 0.05898 | 0.3544 | 0.3766 | **0.046** |
| HC3ASO vs. Z31BSO | 0.04954 | 0.3491 | 0.3673 | **0.039** |
| HC3BRh vs. Z31BSO | 0.1183 | 0.3666 | 0.4158 | **0.003** |
| Z31BRh vs. Z31BSO | 0.09135 | 0.3671 | 0.404 | **0.002** |

ASO, bulk soil before sowing soybean seeds; BSO, surrounding soil at vegetative stage; BRh, rhizosphere soil at vegetative stage.
