# Supplementary material for: Effects of an EPSPS-transgenic soybean line ZUTS31 on root-associated bacterial communities during field growth
Source: PLoS One. 2018 Feb 6;13(2):e0192008. doi: 10.1371/journal.pone.0192008 (PMC5800644; doi:10.1371/journal.pone.0192008)
Supplement: S17 Table — (DOC) [file pone.0192008.s030.doc]

**S17 Table. ANOSIM analysis of bulk soil and surrounding, rhizosphere soil of Z31 and HC3 at vegetative stage based on Bray-Curtis distance metric.**

| Group vs. Group | *R*-value | *P*-value |
| --- | --- | --- |
| **Z31ASO vs. HC3ASO** | -0.1042 | 0.850 |
| **Z31BSO vs. HC3BSO** | 0.0907 | 0.104 |
| **Z31BRh vs. HC3BRh** | 0.1963 | **0.010** |
| Z31ASO vs. Z31BSO | 0.1389 | 0.143 |
| Z31BRh vs. Z31BSO | 0.5852 | **0.005** |
| Z31BRh vs. Z31ASO | 0.9008 | **0.005** |
| HC3BRh vs. Z31BSO | 0.7444 | **0.004** |
| HC3BRh vs. Z31ASO | 0.9921 | **0.005** |
| HC3BSO vs. Z31ASO | 0.9683 | **0.004** |
| HC3BSO vs. Z31BRh | 0.8556 | **0.003** |
| HC3BSO vs. HC3BRh | 0.9111 | **0.005** |
| HC3ASO vs. Z31BSO | 0.0873 | 0.226 |
| HC3ASO vs. Z31BRh | 0.8968 | **0.005** |
| HC3ASO vs. HC3BRh | 0.9683 | **0.003** |
| HC3ASO vs. HC3BSO | 0.8571 | **0.008** |

ASO, bulk soil before sowing soybean seeds; BSO, surrounding soil at vegetative stage; BRh, rhizosphere soil at vegetative stage.
