# Supplementary material for: Effects of an EPSPS-transgenic soybean line ZUTS31 on root-associated bacterial communities during field growth
Source: PLoS One. 2018 Feb 6;13(2):e0192008. doi: 10.1371/journal.pone.0192008 (PMC5800644; doi:10.1371/journal.pone.0192008)
Supplement: S18 Table — (DOC) [file pone.0192008.s031.doc]

**S18 Table. Multiple response permutation procedure (MRPP) analysis of surrounding soil, rhizosphere soil and root endosphere of Z31 and HC3 based on Bray-Curtis distances at flowering stage.**

| Group vs. Group | A | Observed-delta | Expected-delta | *P*-value |
| --- | --- | --- | --- | --- |
| Z31CSO vs. HC3CSO | 0.00206 | 0.3181 | 0.3188 | 0.373 |
| **Z31CRh vs. HC3CRh** | 0.02311 | 0.3080 | 0.3153 | 0.072 |
| **Z31CRt vs. HC3CRt** | 0.05865 | 0.06697 | 0.07114 | 0.055 |
| HC3CRh vs. HC3CSO | 0.1824 | 0.3259 | 0.3987 | **0.002** |
| HC3CRh vs. Z31CSO | 0.1957 | 0.3213 | 0.3995 | **0.003** |
| HC3CRh vs. HC3CRt | 0.6373 | 0.2187 | 0.6028 | **0.008** |
| HC3CRt vs. HC3CSO | 0.6573 | 0.2149 | 0.6269 | **0.003** |
| HC3CRt vs. Z31CSO | 0.6646 | 0.2093 | 0.6239 | **0.004** |
| Z31CRh vs. HC3CSO | 0.1634 | 0.3048 | 0.3643 | **0.004** |
| Z31CRh vs. HC3CRt | 0.6759 | 0.1933 | 0.5964 | **0.006** |
| Z31CRh vs. Z31CSO | 0.1717 | 0.3002 | 0.3624 | **0.003** |
| Z31CRh vs. Z31CRt | 0.6563 | 0.2045 | 0.5950 | **0.005** |
| Z31CRt vs. HC3CSO | 0.6406 | 0.2260 | 0.6288 | **0.005** |
| Z31CRt vs. HC3CRh | 0.6182 | 0.2298 | 0.6021 | **0.004** |
| Z31CRt vs. Z31CSO | 0.6475 | 0.2205 | 0.6254 | **0.004** |

CSO, surrounding soil at flowering stage; CRh, rhizosphere soil at flowering stage; CRt, roots at flowering stage.
