# Supplementary material for: Effects of an EPSPS-transgenic soybean line ZUTS31 on root-associated bacterial communities during field growth
Source: PLoS One. 2018 Feb 6;13(2):e0192008. doi: 10.1371/journal.pone.0192008 (PMC5800644; doi:10.1371/journal.pone.0192008)
Supplement: S19 Table — (DOC) [file pone.0192008.s032.doc]

**S19 Table. Analysis of Similarities (ANOSIM) of surrounding, rhizosphere soil and root endosphere of Z31 and HC3 based on Bray-Curtis distance at flowering stage.**

| Group vs. Group | *R*-value | *P*-value |
| --- | --- | --- |
| Z31CSO vs. HC3CSO | 0.0204 | 0.392 |
| **Z31CRh vs. HC3CRh** | 0.1333 | 0.133 |
| **Z31CRt vs. HC3CRt** | 0.1823 | 0.109 |
| HC3CRh vs. HC3CSO | 0.9667 | **0.003** |
| HC3CRh vs. Z31CSO | 0.9889 | **0.001** |
| HC3CRh vs. HC3CRt | 1.0000 | **0.006** |
| HC3CRt vs. HC3CSO | 1.0000 | **0.004** |
| HC3CRt vs. Z31CSO | 1.0000 | **0.003** |
| Z31CRh vs. HC3CSO | 0.9537 | **0.004** |
| Z31CRh vs. HC3CRt | 1.0000 | **0.006** |
| Z31CRh vs. Z31CSO | 0.9574 | **0.002** |
| Z31CRh vs. Z31CRt | 1.0000 | **0.005** |
| Z31CRt vs. HC3CSO | 1.0000 | **0.004** |
| Z31CRt vs. HC3CRh | 1.0000 | **0.005** |
| Z31CRt vs. Z31CSO | 1.0000 | **0.007** |

CSO, surrounding soil at flowering stage; CRh, rhizosphere soil at flowering stage; CRt, root endosphere at flowering stage.
