# Supplementary material for: Effects of an EPSPS-transgenic soybean line ZUTS31 on root-associated bacterial communities during field growth
Source: PLoS One. 2018 Feb 6;13(2):e0192008. doi: 10.1371/journal.pone.0192008 (PMC5800644; doi:10.1371/journal.pone.0192008)
Supplement: S20 Table — (DOC) [file pone.0192008.s033.doc]

**S20 Table. ADONIS analysis of surrounding soil, rhizosphere soil and roots bacterial communities between Z31 and HC3 based on Bray-Curtis distance at the flowering stage**.

| **Group vs. Group** | **Df** | **Sums Of Sqs** | **Mean Sqs** | **F. Model** | **R^2^** | **Pr(>F)** |
| --- | --- | --- | --- | --- | --- | --- |
| **Z31CSO vs. HC3CSO** | 1(10) | 0.05571(0.52141) | 0.055710(0.052141) | 1.0684 | 0.09653(0.90347) | 0.335 |
| **Z31CRh vs. HC3CRh** | 1(10) | 0.07048(0.49464) | 0.070483(0.049464) | 1.4249 | 0.12472(0.87528) | 0.101 |
| **Z31CRt vs. HC3CRt** | 1(6) | 0.0043681(0.0153474) | 0.0043681(0.0025579) | 1.7077 | 0.22156(0.77844) | 0.081 |
| HC3CRh vs. HC3CSO | 1(10) | 0.37119(0.54827) | 0.37119(0.05483) | 6.7703 | 0.40371(0.59629) | **0.002** |
| HC3CRh vs. Z31CSO | 1(10) | 0.39183(0.53448) | 0.39183(0.05345) | 7.3311 | 0.423(0.577) | **0.001** |
| HC3CRh vs. HC3CRt | 1(8) | 1.88078(0.28528) | 1.88078(0.03566) | 52.743 | 0.8683(0.1317) | **0.001** |
| HC3CRt vs. HC3CSO | 1(8) | 2.10554(0.27221) | 2.10554(0.03403) | 61.881 | 0.88552(0.11448) | **0.001** |
| HC3CRt vs. Z31CSO | 1(8) | 2.11154(0.25842) | 2.1115(0.0323) | 65.368 | 0.89096(0.10904) | **0.009** |
| Z31CRh vs. HC3CSO | 1(10) | 0.28263(0.48157) | 0.282627(0.048157) | 5.8688 | 0.36983(0.63017) | **0.003** |
| Z31CRh vs. HC3CRt | 1(8) | 1.96992(0.21858) | 1.96992(0.02732) | 72.097 | 0.90012(0.09988) | **0.006** |
| Z31CRh vs. Z31CSO | 1(10) | 0.29073(0.46779) | 0.290734(0.046779) | 6.2151 | 0.38329(0.61671) | **0.004** |
| Z31CRh vs. Z31CRt | 1(8) | 1.92303(0.22472) | 1.92303(0.02809) | 68.461 | 0.89537(0.10463) | **0.001** |
| Z31CRt vs. HC3CSO | 1(8) | 2.08585(0.27834) | 2.08585(0.03479) | 59.952 | 0.88227(0.11773) | **0.003** |
| Z31CRt vs. HC3CRh | 1(8) | 1.84012(0.29141) | 1.84012(0.03643) | 50.517 | 0.86329(0.13671) | **0.003** |
| Z31CRt vs. Z31CSO | 1(8) | 2.08851(0.26455) | 2.08851(0.03307) | 63.156 | 0.88757(0.11243) | **0.008** |

An ADONIS difference, which was calculated based on Bray–Curtis distance, is a non-parametric method to measure statistical significance of sample grouping. **, Pr < 0.01. Residuals were presented in parentheses.

CSO, surrounding soil at the flowering stage; CRh, rhizosphere soil at the flowering stage; CRt, roots at the flowering stage.
