# Supplementary material for: Effects of an EPSPS-transgenic soybean line ZUTS31 on root-associated bacterial communities during field growth
Source: PLoS One. 2018 Feb 6;13(2):e0192008. doi: 10.1371/journal.pone.0192008 (PMC5800644; doi:10.1371/journal.pone.0192008)
Supplement: S21 Table — (DOC) [file pone.0192008.s034.doc]

**S21 Table. Multiple response permutation procedure (MRPP) analysis of surrounding soil, rhizosphere soil and roots of Z31 and HC3 based on Bray-Curtis distance at seed-filling stage.**

| Group vs. Group | A | Observed-delta | Expected-delta | *P*-value |
| --- | --- | --- | --- | --- |
| Z31DSO vs. HC3DSO | 0.0221 | 0.3284 | 0.3358 | 0.120 |
| **Z31DRh vs. HC3DRh** | 0.0317 | 0.3751 | 0.3874 | 0.051 |
| **Z31DRt vs. HC3DRt** | -0.0010 | 0.2644 | 0.2642 | 0.478 |
| HC3DRh vs. HC3DSO | 0.0978 | 0.3409 | 0.3779 | **0.003** |
| HC3DRh vs. Z31DSO | 0.1243 | 0.3455 | 0.3946 | **0.002** |
| HC3DRh vs. HC3DRt | 0.5063 | 0.3097 | 0.6272 | **0.003** |
| HC3DRt vs. HC3DSO | 0.5318 | 0.2925 | 0.6248 | **0.001** |
| HC3DRt vs. Z31DSO | 0.5263 | 0.2972 | 0.6274 | **0.003** |
| Z31DRh vs. HC3DSO | 0.1500 | 0.3580 | 0.4212 | **0.004** |
| Z31DRh vs. HC3DRt | 0.4775 | 0.3268 | 0.6254 | **0.003** |
| Z31DRh vs. Z31DSO | 0.1229 | 0.3626 | 0.4134 | **0.006** |
| Z31DRh vs. Z31DRt | 0.4653 | 0.3299 | 0.6170 | **0.004** |
| Z31DRt vs. HC3DSO | 0.5238 | 0.2957 | 0.6208 | **0.003** |
| Z31DRt vs. HC3DRh | 0.4976 | 0.3128 | 0.6227 | **0.002** |
| Z31DRt vs. Z31DSO | 0.5178 | 0.3003 | 0.6228 | **0.002** |

DSO, surrounding soil at seed-filling stage; DRh, rhizosphere soil at seed-filling stage; DRt, root endosphere at seed-filling stage.
