# Supplementary material for: Effects of an EPSPS-transgenic soybean line ZUTS31 on root-associated bacterial communities during field growth
Source: PLoS One. 2018 Feb 6;13(2):e0192008. doi: 10.1371/journal.pone.0192008 (PMC5800644; doi:10.1371/journal.pone.0192008)
Supplement: S22 Table — (DOC) [file pone.0192008.s035.doc]

**S22 Table. Analysis of Similarities (ANOSIM) of surrounding soil, rhizosphere soil and roots bacterial communities of Z31 and HC3 based on Bray-Curtis distance at seed-filling stage.**

| Group vs. Group | *R*-value | *P*-value |
| --- | --- | --- |
| Z31DSO vs. HC3DSO | 0.1167 | 0.159 |
| **Z31DRh vs. HC3DRh** | 0.2139 | 0.056 |
| **Z31DRt vs. HC3DRt** | -0.0574 | 0.626 |
| HC3DRh vs. HC3DSO | 0.5750 | **0.001** |
| HC3DRh vs. Z31DSO | 0.8111 | **0.005** |
| HC3DRh vs. HC3DRt | 1.0000 | **0.005** |
| HC3DRt vs. HC3DSO | 1.0000 | **0.003** |
| HC3DRt vs. Z31DSO | 1.0000 | **0.002** |
| Z31DRh vs. HC3DSO | 0.8574 | **0.003** |
| Z31DRh vs. HC3DRt | 1.0000 | **0.002** |
| Z31DRh vs. Z31DSO | 0.7519 | **0.003** |
| Z31DRh vs. Z31DRt | 1.0000 | **0.005** |
| Z31DRt vs. HC3DSO | 1.0000 | **0.003** |
| Z31DRt vs. HC3DRh | 1.0000 | **0.001** |
| Z31DRt vs. Z31DSO | 1.0000 | **0.006** |

DSO, surrounding soil at seed-filling stage; DRh, rhizosphere soil at seed-filling stage; DRt, roots at seed-filling stage.
