# Supplementary material for: Effects of an EPSPS-transgenic soybean line ZUTS31 on root-associated bacterial communities during field growth
Source: PLoS One. 2018 Feb 6;13(2):e0192008. doi: 10.1371/journal.pone.0192008 (PMC5800644; doi:10.1371/journal.pone.0192008)
Supplement: S23 Table — (DOC) [file pone.0192008.s036.doc]

**S23 Table. ADONIS analysis of surrounding soil, rhizosphere soil and roots bacterial communities between Z31 and HC3 based on Bray-Curtis distance at the seed-filling stage**.

| **Group vs. Group** | **Df** | **Sums Of Sqs** | **Mean Sqs** | **F. Model** | **R^2^** | **Pr(>F)** |
| --- | --- | --- | --- | --- | --- | --- |
| Z31DSO vs. HC3DSO | 1(10) | 0.08632(0.55455) | 0.086319(0.055455) | 1.5566 | 0.13469(0.86531) | 0.097 |
| **Z31DRh vs. HC3DRh** | 1(10) | 0.12156(0.71992) | 0.121561(0.071992) | 1.6885 | 0.14446(0.85554) | **0.041** |
| **Z31DRt vs. HC3DRt** | 1(10) | 0.03110(0.37994) | 0.031103(0.037994) | 0.81862 | 0.07567(0.92433) | 0.536 |
| HC3DRh vs. HC3DSO | 1(10) | 0.21394(0.60028) | 0.213943(0.060028) | 3.564 | 0.26276(0.73724) | **0.001** |
| HC3DRh vs. Z31DSO | 1(10) | 0.26865(0.61246) | 0.268646(0.061246) | 4.3864 | 0.3049(0.6951) | **0.001** |
| HC3DRh vs. HC3DRt | 1(10) | 2.12849(0.51757) | 2.12849(0.05176) | 41.125 | 0.8044(0.1956) | **0.001** |
| HC3DRt vs. HC3DSO | 1(10) | 2.21032(0.45966) | 2.21032(0.04597) | 48.086 | 0.82784(0.17216) | **0.001** |
| HC3DRt vs. Z31DSO | 1(10) | 2.20894(0.47183) | 2.20894(0.04718) | 46.816 | 0.82399(0.17601) | **0.004** |
| Z31DRh vs. HC3DSO | 1(10) | 0.35272(0.66201) | 0.35272(0.06620) | 5.328 | 0.3476(0.6524) | **0.001** |
| Z31DRh vs. HC3DRt | 1(10) | 2.0046(0.5793) | 2.00457(0.05793) | 34.603 | 0.7758(0.2242) | **0.001** |
| Z31DRh vs. Z31DSO | 1(10) | 0.29819(0.67419) | 0.298193(0.067419) | 4.423 | 0.30666(0.69334) | **0.006** |
| Z31DRh vs. Z31DRt | 1(10) | 1.90939(0.58229) | 1.90939(0.05823) | 32.791 | 0.76631(0.23369) | **0.001** |
| Z31DRt vs. HC3DSO | 1(10) | 2.15589(0.46266) | 2.15589(0.04627) | 46.598 | 0.82332(0.17668) | **0.005** |
| Z31DRt vs. HC3DRh | 1(10) | 2.06867(0.52056) | 2.06867(0.05206) | 39.739 | 0.79895(0.20105) | **0.005** |
| Z31DRt vs. Z31DSO | 1(10) | 2.14809(0.47483) | 2.14809(0.04748) | 45.239 | 0.81897(0.18103) | **0.005** |

An ADONIS difference, which was calculated based on Bray–Curtis distance, is a non-parametric method to measure statistical significance of sample grouping. Residuals were presented in parentheses.

DSO, surrounding soil at seed-filling stage; DRh, rhizosphere soil at seed-filling stage; DRt, roots at seed-filling stage.
